# Barriers and facilitators to the uptake of electronic collection and use of patient-reported measures in routine care of older adults: a systematic review with qualitative evidence synthesis

**DOI:** 10.1093/jamiaopen/ooae068

**Published:** 2024-08-02

**Authors:** Gayanika M Hettiarachchi Senarath, Pari Delir Haghighi, Lu Bai, Michelle M Shannon, Nadine E Andrew, Velandai Srikanth, David A Snowdon, Denise A O’Connor

**Affiliations:** Department of Human-Centred Computing, Faculty of Information Technology, Monash University, Clayton, VIC 3800, Australia; Department of Human-Centred Computing, Faculty of Information Technology, Monash University, Clayton, VIC 3800, Australia; Department of Human-Centred Computing, Faculty of Information Technology, Monash University, Clayton, VIC 3800, Australia; Academic Unit, Frankston Hospital, Peninsula Health, Frankston, VIC 3199, Australia; Academic Unit, Frankston Hospital, Peninsula Health, Frankston, VIC 3199, Australia; Department of Medicine, Peninsula Clinical School, Central Clinical School, Monash University, Frankston, VIC 3199, Australia; National Centre for Healthy Ageing, Frankston, VIC 3199, Australia; Academic Unit, Frankston Hospital, Peninsula Health, Frankston, VIC 3199, Australia; Department of Medicine, Peninsula Clinical School, Central Clinical School, Monash University, Frankston, VIC 3199, Australia; National Centre for Healthy Ageing, Frankston, VIC 3199, Australia; Academic Unit, Frankston Hospital, Peninsula Health, Frankston, VIC 3199, Australia; Department of Medicine, Peninsula Clinical School, Central Clinical School, Monash University, Frankston, VIC 3199, Australia; National Centre for Healthy Ageing, Frankston, VIC 3199, Australia; School of Public Health and Preventive Medicine, Monash University, Melbourne, VIC 3004, Australia

**Keywords:** electronic patient reported outcome measure, electronic patient reported experience measure, barriers, facilitators, implementation science

## Abstract

**Objective:**

The aims of this systematic review were to (1) synthesize the available qualitative evidence on the barriers and facilitators influencing implementation of the electronic collection and use of patient-reported measures (PRMs) in older adults’ care from various stakeholder perspectives and (2) map these factors to the digital technology implementation framework *Non-adoption, Abandonment, challenges to the Scale-up, Spread, Sustainability* (NASSS) and behavior change framework *Capability, Opportunity, Motivation, Behaviour* (COM-B).

**Materials and Methods:**

A search of MEDLINE, CINAHL Plus, and Web of Science databases from 1 January 2001 to 27 October 2021 was conducted and included English language qualitative studies exploring stakeholder perspectives on the electronic collection and use of PRMs in older adults’ care. Two authors independently screened studies, conducted data extraction, quality appraisal using the Critical Appraisal Skills Programme (CASP), data coding, assessed confidence in review findings using Grading of Recommendations Assessment, Development, and Evaluation Confidence in the Evidence from Reviews of Qualitative Research (GRADE CERQual), and mapped the findings to NASSS and COM-B. An inductive approach was used to synthesize findings describing the stakeholder perspectives of barriers and facilitators.

**Results:**

Twenty-two studies were included from the 3368 records identified. Studies explored older adult, caregiver, healthcare professional, and administrative staff perspectives. Twenty nine of 34 review findings (85%) were graded as having high or moderate confidence. Key factors salient to older adults related to clinical conditions and socio-cultural factors, digital literacy, access to digital technology, and user interface. Factors salient to healthcare professionals related to resource availability to collect and use PRMs, and value of PRMs collection and use.

**Conclusion:**

Future efforts to implement electronic collection and use of PRMs in older adults’ care should consider addressing the barriers, facilitators, and key theoretical domains identified in this review. Older adults are more likely to adopt electronic completion of PRMs when barriers associated with digital technology access, digital literacy, and user interface are addressed. Future research should explore the perspectives of other stakeholders, including those of organizational leaders, digital technology developers and implementation specialists, in various healthcare settings and explore factors influencing implementation of PREMs.

**PROSPERO registration number:**

CRD42022295894

## Introduction

Patient Reported Outcome Measures (PROMs) and Patient Reported Experience Measures (PREMs), collectively referred to as Patient Reported Measures (PRMs), capture patient perceptions of health (eg, symptoms, functional status) and experiences (eg, accessibility of services, patient-healthcare professional interaction) with healthcare services. PROMs are validated tools in the form of questionnaires used to report the treatment outcomes perceived by patients (also known as Patient Reported Outcomes (PROs)).^1^ Consideration of PRMs is integral to achieving value-based care for older adults given these individuals frequently experience multimorbidity and are more likely to have complex care needs.^2^

PRMs are used to monitor population health, assess the effects of clinical interventions in randomized trials, and more recently to inform the provision of routine clinical care.^3^ In routine care, they seek to complement clinical and health provider reported outcomes by providing data about patients’ perceptions of their own health, and their needs, preferences and values.^2^ Several systematic reviews have found that use of PRMs in routine clinical care leads to improved patient-provider communication and greater patient satisfaction, and may improve monitoring of treatment response and detection of unrecognized problems.^4–6^ In oncology settings, the use of PROMs has improved management of patient symptoms and reduced hospitalizations.^7^ A potential benefit of using PREMs in clinical practice is the quality improvement of care processes.^8^ Studies have reported the use of PRM tools in the routine care of older adults in community, palliative, outpatient, and emergency care settings.^9–11^

Despite the demonstrated benefits of using PRMs in clinical practice, their sustained implementation in routine care, including for older adults, remains suboptimal.^12^^,^^13^ Electronic collection and use of PRMs have been advocated as an alternative to traditional, paper-based PRM methods to increase uptake, and reduce administrative burden and costs in routine older adults’ care.^14^^,^^15^ Benefits of the electronic mode include streamlining of data collection processes, improving integration with existing information systems, and enhancing accessibility for healthcare providers.^14^^,^^16^^,^^17^ However, electronic completion of PRMs pose unique challenges in the context of older adults’ care, such as aging related visual, cognitive, and functional limitations.^10^ While a review of the factors influencing uptake of electronic administration of PRMs across health care more broadly is available,^18^ a systematic review of the barriers and facilitators to the uptake of electronic administration of PRMs specifically in the context of older adults’ care is needed to identify the unique issues faced in this context and key targets for change when designing implementation efforts. The synthesis of evidence from primary qualitative studies is ideally suited for establishing a greater understanding of the issues influencing implementation, through a rich interpretation of experiences.^19^

Therefore, the aims of this systematic review were to: (1) synthesize the available qualitative evidence on the barriers and facilitators influencing the uptake of electronic collection and use of PRMs in older adults’ care from the perspectives of older adults and/or their caregivers and healthcare service staff and (2) to map these factors to contemporary implementation science frameworks to inform future implementation efforts.

## Methods

### Protocol development and registration

A systematic review protocol^20^ was developed in accordance with the Preferred Reporting Items for Systematic Review and Meta-Analysis Protocols (PRISMA-P) 2015 statement^21^ and the methods described by the Cochrane Qualitative and Implementation Methods Groups and the Cochrane Handbook.^22^ The manuscript is reported in accordance with PRISMA reporting guidelines^23^^,^^24^ and the Enhancing Transparency in Reporting the Synthesis of Qualitative Research statement (ENTREQ).^25^

### Eligibility criteria

The study inclusion and exclusion criteria (Supplementary Appendix S1) were developed using the Perspective, Setting, Phenomenon of interest, Environment, Comparison, Time/Timing and Findings (PerSPecTIF) question framework for evaluating evidence relevant to complex interventions.^26^ Peer-reviewed, English language full-text qualitative or mixed-method studies were included if:

- qualitative data collection and analysis methods were used;
- participants included older adults aged 65 years and above, or stakeholders (eg, caregivers, healthcare service staff such as healthcare professionals and administrative staff) involved in the care of older adults;
- the setting was any healthcare setting and any environment except clinical trials;
- reported outcomes included experiences, attitudes, preferences, beliefs and perceptions of electronic collection and/or use of PRMs in older adults’ care from any of the stakeholders specified above; and
- the stage of digital change included planning, development, implementation or use.

### Search methods

MEDLINE (via OVID), Cumulative Index to Nursing and Allied Health Literature (CINAHL) Plus (via EBSCO Host), and Web of Science databases were searched from 1 January 2001 to 27 October 2021. The search strategy (Supplementary Appendix S2) included three main concepts: “patient-reported measures,” “electronic surveys or questionnaires,” and “qualitative research.” Additional potentially relevant studies were identified by searching the reference lists of the included studies.

### Selection of studies

All citations and potentially relevant full-text articles were independently screened by two authors (GHS or PDH). Any disagreements were resolved through discussion with a third review author (DOC). The search and screening results were summarized in a PRISMA flow diagram.^24^

### Quality assessment

The quality of the included studies was independently assessed by two of three authors (GHS, MMS, LB) using the Critical Appraisal Skills Programme (CASP) checklist.^27^ Any disagreements were resolved through discussion with a third review author (DAS or DOC). All eligible studies were included irrespective of quality.

### Data extraction

Two of three authors (GHS, MMS, LB) independently extracted qualitative data from included studies (ie, primary results and secondary analysis) using a standardized data collection form. This was imported into an excel spreadsheet and Nvivo 12 software^28^ for coding.

An inductive coding approach^29^ was used to code the extracted data “line-by-line” and derive codes as each study was reviewed (first step). Descriptive findings were then developed from these codes (second step). All studies were independently coded by two of three authors (GHS, MMS, LB). The findings, consisting of barriers and facilitators, were developed by the primary author and reviewed and refined by the wider review team. Findings were categorized as those unique to the electronic collection and use of PRMs, and those related to PRMs implementation in general.

The barriers and facilitators were then independently mapped to two implementation science theoretical frameworks by two authors (GHS and LB). Non-adoption, Abandonment, challenges to the Scale-up, Spread and Sustainability (NASSS), a healthcare digital technology change implementation framework^30^ and Capability, Opportunity, Motivation, Behaviour (COM-B), a behavior change framework^31^ were selected and used in combination in this review. NASSS was considered relevant for understanding multi-level factors (ie, organizational and wider context) influencing digital innovations in healthcare and COM-B was considered relevant for understanding factors influencing individual-level adopter behavior. Findings were first mapped to NASSS domains, and this mapping was then aligned with relevant COM-B domains. The mapping of the barriers and facilitators to the theoretical domains was discussed and refined until consensus was achieved amongst the review team.

### Confidence assessment

Two of three authors (GHS, MMS, LB) independently assessed the confidence in the review findings using the Grading of Recommendations Assessment, Development, and Evaluation Confidence in the Evidence from Reviews of Qualitative Research (GRADE CERQual) tool.^32^ The following four components were considered in assessing the confidence in each review finding:

- The extent to which there are *methodological limitations* of studies contributing to a review finding based on CASP assessments.
- The extent to which data from studies supporting a review finding are *relevant* to the review question (ie, population, phenomenon of interest).
- The extent to which the review finding is *coherent* (ie, well-supported) with data from studies.
- The determination that data from studies supporting review finding is *adequate* (ie, richness and quantity of data).

Each component was individually rated as *no or very minor concerns*, *minor concerns*, *moderate concerns*, or *serious concerns*. A judgement on the overall confidence of a review finding was made either as *high* (ie, highly likely that the review finding reasonably represents the phenomenon of interest); *moderate* (ie, likely that the review finding reasonably represents the phenomenon of interest); *low* (ie, possible that the review finding reasonably represents the phenomenon of interest); or *very low* (ie, unclear if the review finding reasonably represents the phenomenon of interest). The confidence assessment started as high by default and was downgraded depending on the severity of concerns and number of domains showing concerns. Discrepancies were resolved through discussion among the review team.

## Results

### Results of search and study selection

**Figure 1.**
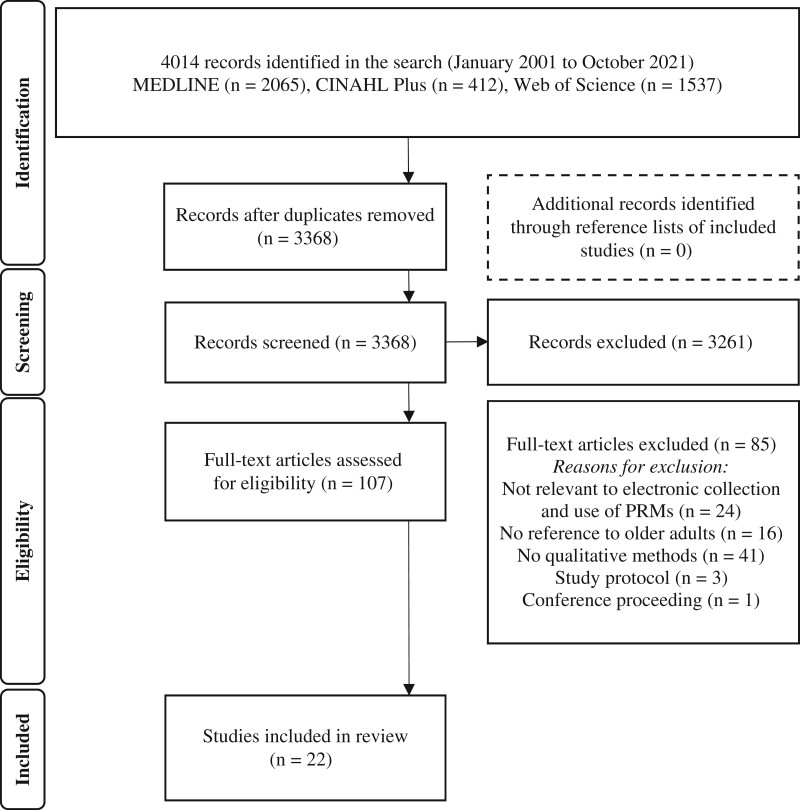
PRISMA Chart. Created by the authors in accordance with PRISMA guidelines. Abbreviations: CINAHL = Cumulative Index to Nursing and Allied Health Literature; PRISMA = Preferred Reporting Items of Systematic Reviews and Meta-Analysis; PRM = patient-reported measures.

As shown in the PRISMA flowchart (Figure 1), we identified 3368 titles and abstracts after removing duplicates from the electronic database searches, of which 107 full-text records were screened for inclusion. We excluded 85 studies due to: lack of relevance to electronic collection and use of PRMs (*n* = 24), no reference to older adults (*n* = 16), qualitative methods not utilized (*n* = 41), was a protocol (*n* = 3), or conference proceeding (*n* = 1). Twenty-two studies met our inclusion criteria and were included in this review.

### Description of included studies

The characteristics of the included studies are described in Table 1 (Supplementary Appendix S3 provides additional information). Studies were conducted in the United States (*n* = 9),^10^^,^^36–38^^,^^40^^,^^43^^,^^47^^,^^51^ United Kingdom (*n* = 1),^33^ Netherlands (*n* = 2),^34^^,^^50^ Canada (*n* = 5),^35^^,^^39^^,^^41^^,^^45^^,^^52^ Denmark (*n* = 4),^42^^,^^44^^,^^46^^,^^48^ and Austria (*n* = 1).^49^ The studies sampled participants from the outpatient setting (*n* = 19),^10^^,^^33–36^^,^^38^^,^^40–50^^,^^52^^,^^53^ inpatient setting (*n* = 1),^39^ and a combination of inpatient and outpatient settings (*n* = 1).^51^ The healthcare setting could not be determined for one study.^37^ Studies explored patients’ perspectives (n = 9),^33^^,^^37^^,^^40^^,^^42^^,^^45^^,^^47^^,^^48^^,^^49^^,^^52^ healthcare professionals’ perspectives (n = 6),^34^^,^^38^^,^^39^^,^^41^^,^^50^^,^^53^ patients’ and healthcare professionals’ perspectives (n = 4),^43^^,^^44^^,^^46^^,^^51^ patients’, healthcare professionals’ and administrative staff perspectives (*n* = 1),^35^ healthcare professionals’ and administrative staff perspectives (*n* = 1)^36^ and patients’, caregivers’, and administrative staff perspectives (*n* = 1).^10^ The studies included 374 patients, 183 healthcare professionals, 3 caregivers, and 5 administrative staff. Fifteen studies included qualitative interviews,^10^^,^^35–37^^,^^41^^,^^43^^,^^45–53^ three used interviews and focus groups,^39^^,^^42^^,^^44^ and single studies used either focus groups,^40^ surveys with open-ended questions,^34^ interviews and observations,^33^ or interviews and surveys with open-ended questions.^38^

**Table 1.** Characteristics of included studies (*N* = 22)

Reference and country	Healthcare setting	Study Type	Patients’ characteristics	Other stakeholders’ characteristics	Data collection method(s)	Data analysis method	Stage of digital change focused	**Methodology limitations** ^a^
Long et al^10^ *USA*	Outpatient—orthopaedic surgery	Qualitative	• Patients (*n* = 12) • Age range: 20-81 years (not limited to patients); Median age: 44.5 years	• Caregivers (*n* = 3) • Administrative staff (*n* = 2)	Semi-structured interviews	Framework analysis	Use (outpatient visit related)	None
Aiyegbusi et al^33^ *UK*	Outpatient—nephrology	Mixed-methods	• Patients (*n* = 8) • Age range: 36-87 years; Mean age: 64.3 years	–	Interviews and observations	Content analysis	Development (usability assessment)	Major (Research design, data collection, data analysis)
Amini et al^34^ *Netherlands*	Outpatient—multidisciplinary care	Mixed-methods	–	• Healthcare professionals (*n* = 61): • Physicians (*n* = 31) • Nursing (*n* = 15) • Allied health (*n* = 7) • Physician researchers (*n* = 2) • Physician assistant (*n* = 2) • Other (*n* = 4)	Survey containing open-ended questions	Content analysis	Planning, Development and Implementation	Major (Recruitment strategy, researcher-participant relationship, data analysis)
Kaur et al^35^ *Canada*	Outpatient—cosmetic surgery	Qualitative	• Patients (*n* = 11) • Age range: 18-83 years; Mean age: 43 years	• Healthcare professionals: Surgeons (*n* = 3) • Administrative staff (*n* = not reported)	Semi-structured interviews	Thematic analysis using inductive approach	Development	Major (Recruitment strategy, data collection, researcher-participant relationship, data analysis)
Spaulding et al^36^ *USA*	Outpatient—psychiatry	Qualitative	• Age range: 19-100 years; Mean age: 53.3 years	• Healthcare professionals (*n* = 6): • Physicians (*n* = 4) • Nursing (*n* = 2) • Administrative staff: Front desk staff (*n* = 3)	Semi-structured interviews	Thematic analysis using deductive approach, guided by RE-AIM framework	Implementation	Minor (Researcher-participant relationship)
Delgado-Herrera et al^37^ *USA*	Setting unclear—gastroenterology	Qualitative	• Patients (*n* = 25) • Age range: 26.1-79.2 years; Mean age: 52.7 years	–	Open-ended interviews	Content analysis	Development (usability assessment)	Minor (Recruitment strategy, researcher-participant relationship)
Mou et al^38^ *USA*	Outpatient—multidisciplinary care	Mixed-methods	–	• Healthcare professionals: Primary Care Providers (n = 28)	Semi-structured interviews Survey containing open-ended questions	Thematic analysis using a deductive approach Content analysis	Implementation	Minor (Recruitment strategy, researcher-participant relationship)
Krawczyk et al^39^ *Canada*	Inpatient (hospital-based acute care)—palliative care	Qualitative	–	• Healthcare professionals (*n* = 25): • Physicians (*n* = 2) • Nursing (*n* = 19) • Allied Health (*n* = 4)	Focus groups and interviews	Thematic analysis using inductive approach, guided by micro-meso-macro framework	Development (design), Implementation and Use	Minor (Recruitment strategy, researcher-participant relationship)
Navarro-Millán et al^40^ *USA*	Outpatient—rheumatology	Qualitative	• Patients (*n* = 31) • Age range: 25-84 years; Mean age: 51 years	–	Focus groups	Thematic analysis using deductive approach, guided by Andersen-Newman framework	Planning	Minor (Researcher-participant relationship)
Schick-Makaroff and Molzahn^41^ *Canada*	Outpatient—nephrology	Mixed-methods	• Age range: 34-88 years; Mean age: 67 years	• Healthcare professionals: Nurses (*n* = 11)	Semi-structured interviews	Interpretive description using inductive approach	Use (outpatient visit related)	None
Brochmann et al^42^ *Denmark*	Outpatient—oncology (hematology)	Mixed-methods	• Patients (*n* = 17): Above 60 years (*n* = 8) • Age range: 39-80 years; Mean age of patients 60 years and above: 72 years	–	Focus groups and interviews	Content analysis using deductive approach	Development (feasibility)	Major (Research design, researcher-participant relationship, data analysis)
Snyder et al^43^ *USA*	Outpatient—oncology	Mixed-methods	• Patients (*n* = 47) • Age range: 28-81 years; Median age: 58 years	• Healthcare professionals: Clinicians (*n* = 11)	Open-ended interviews	Content analysis	Development (pilot-testing)	Major (Researcher-participant relationship, data analysis, statement of findings)
Tolstrup et al^44^ *Denmark*	Outpatient—oncology	Mixed-methods	• Patients (*n* = 14) • Age range: 41-79 years; Median age: 67 years	• Healthcare professionals (*n* = 5): • Physicians (*n* = 3) • Nurses (*n* = 2)	Semi-structured interviews and Focus group	Content analysis using deductive approach	Implementation	None
Yamada et al^45^ *Canada*	Outpatient—respirology	Qualitative	• Patients (*n* = 12) • Age range: 25–60 years and above	–	Semi-structured interviews	Directed content analysis guided by TDF	Use (outpatient visit related)	None
Baeksted et al^46^ *Denmark*	Outpatient—oncology	Mixed-methods	• Patients (*n* = 4) • Age range: 56-79 years; Median age: 69 years	• Healthcare professionals: Oncologists (*n* = 5)	Semi-structured interviews	Thematic analysis using deductive approach	Development (feasibility)	Minor (Data analysis)
Samuel et al^47^ *USA*	Outpatient—oncology	Mixed-methods	• Patients (*n* = 40) • Mean age: Black—62.1; White—66.9	–	Semi-structured interviews	Thematic analysis using inductive approach	Use (outpatient visit related)	Minor (Researcher-participant relationship)
Nielsen et al^48^ *Denmark*	Outpatient—gastroenterology	Qualitative	• Patients (*n* = 16): 65 years and above (*n* = 2) • Age range: 29-73 years; Mean age: 49.3 years	–	Semi-structured interviews	Thematic analysis using abductive approach guided by the ReadyHy framework	Use (outpatient visit related)	None
Lehmann et al^49^ *Austria*	Outpatient—oncology	Mixed-methods	• Patients (*n* = 102) • Age range: 39-83 years; Mean age: 59.9 years	–	Semi-structured interviews	Content analysis	Use (outpatient visit related)	Minor (Data analysis, statement of findings)
Duman-Lubberding et al^50^ *Netherlands*	Outpatient—Oncology	Mixed-methods	• Mean age: 63.5 years	• Healthcare professionals: Surgeons (*n* = 6)	Semi-structured interviews	Thematic analysis using inductive approach	Use (outpatient visit related)	Major (Research design, recruitment strategy, data collection, researcher-participant relationship, data analysis)
Grossman et al^51^ *USA*	Inpatient and outpatient—cardiology	Mixed-methods	*Stages 1 and 3:* • Patients (*n* = 25) • Age range: 30-78 years; Median age: Stage 1-53 years; Stage 2-57 years	*Stage 1:* • Healthcare professionals (*n* = 11): • Physicians (*n* = 8) • Nurse practitioners (*n* = 3)	Semi-structured interviews	Thematic analysis	Development (usability)	None
Moradian et al^52^ *Canada*	Outpatient—oncology	Mixed-methods	• Patients (*n* = 10) • Age range: 18-78 years; Mean age: 68 years	–	Semi-structured interviews	Thematic analysis	Development (usability)	Minor (Researcher-participant relationship)
Sandhu et al^53^ *USA*	Outpatient—*oncology* (multiple clinical domains)	Qualitative	–	• Healthcare professionals: Oncologists (*n* = 16)	Semi-structured interviews	Thematic analysis using inductive approach	Implementation	None

Table created by the authors.

a Study quality assessment: “None”: all “yes” answers on CASP assessment; “minor”: ≤2 “no” answers on CASP assessment; “major”: >2 “no” answers on CASP assessment.

Abbreviations: RE-AIM = Reach, Effectiveness, Adoption, Implementation, Maintenance; TDF = Theoretical Domains Framework.

### Methodological limitations of studies

The results of the CASP assessment (Supplementary Appendix S4) indicated that all studies had a clear statement of aims, used appropriate qualitative methodology and considered ethical issues. Most studies had appropriate research designs (*n* = 19), adequately justified data collection (*n* = 19), and provided a clear statement of findings (*n* = 19). Methodological limitations included inadequate explanation and/or consideration of the recruitment strategy (*n* = 7), inadequate explanation and/or consideration of the relationship between participants and researchers (*n* = 12), and insufficient rigor in data analysis (*n* = 7). Sixteen studies had minor or no methodological limitations and six studies were judged to have major methodological limitations.

### Synthesis of findings

Thirty-four findings consisting of barriers and facilitators from the perspectives of different stakeholders were derived (Supplementary Appendix S5 provides detail on these findings). Supportive quotes for all findings are presented in Supplementary Appendix S6. The review findings were grouped into 11 thematic categories: (1) older adult’s characteristics; (2) digital technology; (3) support from social circle; (4) knowledge and skills; (5) motivation and incentives for capture and use of PRMs; (6) emotional experience; (7) older adults’ autonomy; (8) patient—healthcare professional communication; (9) workflow; (10) organizational factors; and (11) PRMs questionnaire selection and design. Findings were graded as high confidence (*n* = 3), moderate confidence (*n* = 26), and low confidence (*n* = 5). Findings that were graded as high confidence included: (1) user interface for healthcare professionals; (2) regular exposure enhancing health knowledge; and (3) questionnaire length and complexity of questions. The confidence of a review finding was typically downgraded due to methodological limitations, data adequacy limitations (ie, few studies contributing to the finding) and relevance (ie, low number of older adults in contributing studies). The confidence assessments are reported in the CERQual evidence profiles, in Supplementary Appendix S7.

Table 2 illustrates the barriers and facilitators from different stakeholder perspectives mapped to the theoretical domains of NASSS and COM-B frameworks. Barriers and facilitators unique to the electronic collection and use of PRMs are outlined in Table 3. Findings relevant to both electronic and general implementation of PRMs, such as *support from social circle to complete PRMs*, have been elaborated in supplemental appendix 5. A brief summary of the findings, organized by the NASSS framework with reference to relevant COM-B domains, is described below.

**Table 2.** Barriers and facilitators to uptake of electronic collection and use of PRMs mapped to the theoretical domains of NASSS and COM-B frameworks.

NASSS	COM-B	Older adults	Healthcare Professionals	Administrative staff (front desk)	Caregivers
Patient condition^a^: clinical condition, comorbidities, sociocultural and socio-economic aspects that determine individual’s ability to use digital technology	*Capability* (Memory, attention and decision processes)	Clinical conditions and socio-cultural factors—F1 (B)	Clinical conditions and socio-cultural factors—F1 (B)	Clinical conditions and socio-cultural factors—F1 (B)	Clinical conditions and socio-cultural factors—F1 (B)
	*Capability* (Knowledge; Skills)	Digital knowledge and skills—F10 (B)(F) Health knowledge and literacy in general among older adults—F11 (B)	Digital knowledge and skills—F10 (B) Health knowledge and literacy in general among older adults—F11 (B)	Digital knowledge and skills—F10 (B) Health knowledge and literacy in general among older adults—F11 (B)	Health knowledge and literacy in general among older adults—F11 (B)
	*Opportunity* (Environmental context and resources)	Access to digital technology—F2 (B)(F)	Access to digital technology—F2 (B)(F)	–	–
Technology: digital technology equipment, features and functionality, knowledge generated knowledge and support needed, and supply model	*Opportunity* (Environmental context and resources)	User interface for older adults—F3, F4 (B)(F) Electronic device for PRMs completion—F6 (B)(F) Technical challenges—F7 (B)	User interface for older adults—F3, F4 (B)(F) User interface for healthcare professionals—F5 (B)(F) Technical challenges—F7 (B)	User interface for older adults—F3 (F)	User interface for older adults—F4 (B)
	*Motivation* (Beliefs about consequences)	Privacy and security of personal data—F8 (B)(F)	Privacy and security of personal data—F8 (B)	–	–
Value proposition: whether or not the digital technology is worth developing and implementing for older adults, healthcare professionals, healthcare providers and suppliers	*Motivation* (Beliefs about consequences)	PRMs utilization—F15, F16, F17, F18 (B)(F) Location of PRMs collection—F20 (B)(F) Timing and frequency of PRMs completion—F21 (B)(F) Access to additional resources based on PRMs responses—F22 (B)(F) Patient—healthcare professional communication—F25 (B)(F)	PRMs utilization—F15, F16, F17, F18 (B)(F) PRMs data completeness—F17, F18 (B)(F) Enhanced clinical documentation—F17 (F) Access to PRMs responses—F17, F18 (B)(F) Discrepancies in patient’s assessment of health—F18 (B) Location of PRMs collection—F20 (B)(F) Timing and frequency of PRMs completion—F21 (B)(F) Access to additional resources based on PRMs responses—F22 (B)(F) Patient—healthcare professional communication—F25 (B)(F) Automated administration of PRMs—F26 (F)	–	–
Adopter system: changes in role, identity, and acceptance of digital technology by different stakeholders	*Capability* (Knowledge; Skills)	Rationale for PRMs collection and use—F14 (B)	PRMs interpretation knowledge and skills—F12 (B) Rationale for PRMs collection and use—F14 (B)	–	–
	*Capability* (Memory, attention and decision processes)	Questionnaire length and complexity of questions—F32 (B)(F) Questionnaire relevance to patient’s health—F33 (B)(F) PRMs response capture option—F34 (B)(F)	Questionnaire length and complexity of questions—F32 (B)(F) Questionnaire relevance to patient’s health—F33 (B)(F) PRMs response capture option—F34 (B)(F)	–	–
	*Motivation* (Beliefs about consequences)	Regular exposure enhancing older adults’ health knowledge—F13 (F) Efficiencies, time constraints and changes to work routines—F26 (F) Additional work with PRMs completion—F27 (B)	Regular exposure enhancing older adults’ health knowledge—F13 (F) Efficiencies, time constraints and changes to work routines—F26 (B) (F) Additional work with PRMs completion—F27 (B)	Acceptance of intervention by older adults—F19 (F) Efficiencies, time constraints and changes to work routines—F26 (F)	–
	*Motivation* (Emotions)	Emotional experiences—F23 (B)(F)	Emotional experiences—F23 (B)(F)	–	–
	*Motivation* (Intentions)	Older adults’ owning responsibility to improve own health—F15 (F)	–	–	–
	*Motivation* (Optimism)	–	Healthcare professional’s age and length of service—F18 (B)	–	–
	*Opportunity* (Social influences)	Support from social circle—F9 (F) Trust on healthcare service—F15 (F) Older adults’ autonomy—F24 (B)	Support from social circle—F9 (F) Older adults’ autonomy—F24 (B)	Support from social circle—F9 (F)	Support from social circle—F9 (F)
Organization: readiness, capacity, and work needed to implement digital technology in organization	*Opportunity* (Environmental context and resources)	Change management—F30 (F) Resources—F31: - Education (F) -Technical support (F)	Team culture and collaboration—F28 (B)(F) Leadership and champions—F29 (F) Change management—F30 (B)(F) Resources—F31: - Education (B)(F) - IT infrastructure (B)(F) - Support staff (B)(F) - Funding (B)(F)	Change management—F30 (F) Resources—F31: - Education (F) - Technical support (F)	–
Wider context: policy context, regulatory or professional body influence, public perceptions and inter-organizational networking	*Opportunity* (Environmental context and resources)	–	Regulatory directives—F17, F18 (B)(F) Resources—F31: - Funding (F) *Government funding to cover electronic system costs*	–	–

Barrier—(B); Facilitator—(F). F1 to F34—Findings describing stakeholder perspectives found in Supplemental Appendix S5. Theoretical Domains Framework (TDF) domains relevant to COM-B are also listed.

a Factors reported in the patient condition domain also apply to the adopter system domain.

**Table 3.** Barriers and facilitators unique to the digital aspects of electronic collection and use of PRMs with illustrative quotes and interpretations.

Barrier (B)/Facilitator (F)	Example quotes/interpretations
Clinical conditions and socio-cultural factors—F1 (B) *Pt*	*Dexterity impairment:* Patients had varying levels of dexterity issues controlling the mouse. Some had difficulty controlling the cursor—*Nothing complicated … it’s controlling the mouse*^33^ ***Pt*** *Visual impairment:* One patient reported his poor eyesight made using the iPad difficult^51^***Pt***
Access to digital technology—F2 (B)(F) *Pt HCP*	*A lot of my patients don’t use MyChart, they don’t have email, older lung cancer patients that live out in the country, they just don’t have that technology available.* ^53^ ***HCP*** Participants that owned either a portable (laptop or tablet) or desktop computer or smartphone, or a combination of the 3, were comfortable accessing the internet and checking electronic mail (e-mail)^35^ ***Pt***
User interface for older adults—F3, F4 (B)(F) *Pt C Adm HCP*	*it’s frustrating when you have to go in and out, in and out, you think you’re finished and then you get an email saying you’re not finished* ^10^ ***C*** *I do not like seeing how many more pages I have. It is just like oh, I have five more pages to go* ^35^ ***Pt*** *For elderly patients or people that are visually impaired and [PROs] can be stressful for them to click through all of the screens because it’s a lot of questions* ^38^ ***HCP*** Participants noted that the psychological aspect of not having to view all the questions (as with the paper format) can be less overwhelming for patients. In the ePSRM tool, questions are presented one at a time^36^ ***HCP Adm***
User interface for healthcare professionals—F5 (B)(F) *HCP*	*The frustration of even finding the PROs is pretty high. [Once I find the data] I can't even tell which [PRO question] is which because they're just all like shoved down there into one big blob of* text. I *wish there were a way to just visually tell like a like a good dashboard in your car if it’s a red light that means there’s something wrong*^38^ ***HCP***
Electronic device of PRMs completion—F6 (B)(F) *Pt*	*I’m pretty competent on the internet and maybe on an iPad like on a larger device it might have been more free flowing* ^45^ ***Pt***
Technical challenges—F7 (B) *Pt HCP*	*There was some sort of technical difficulty at… their end of it. Because it just went blank on me… where the phone call is made, and the beginning of the survey is just—it was like there was something wrong technically at the other end, because the phone just went dead* ^47^ ***Pt*** *[Patients] get frustrated with [the PRO tablets] if they log themselves out. They have to enter the encounter ID again… [which] they don’t remember* ^38^ ***HCP***
Privacy and security of personal data—F8 (B)(F) *Pt HCP*	*I would want to know who had access to it (PROM data) and what it was being used for. Like I would want to know those things before I decided whether I was going to complete it. And I think like I would only want the surgeon and key staff in his office to have access to it, and I think it should only be used for improving your surgical care, like your results…* ^35^ ***Pt*** The surgeons were also concerned regarding the ownership and privacy of the data when utilizing the TickiT platform^34^ ***HCP***
Support from social circle—F9 (F) *Pt HCP*	*Once my older patients get some help from the front desk and realize that it's going to be a tap they, they seem to do well* ^36^ ***HCP*** *I’m all about apps and stuff like that. But for people who aren’t, have someone in the office to show them, walk them through it step by step and make sure that they’re okay with it before they leave* ^40^ ***Pt***
Digital knowledge and skills—F10 (B)(F) *Pt Adm HCP*	*For older adults:* *When I first got the tablet in my hand, a little anxiety came over me because I’m not much into computers […] when you’re doing something you’re not familiar with, I get overwhelmed a little* ^10^ ***Pt*** Staff reported that elderly patients more often had difficulty completing PROMs due to low computer and/or technology literacy^10^ ***Adm*** *.. they just don’t have that technology available. And even if they do, like a smartphone or a computer, they don’t feel comfortable. Just filling out a little survey with a mouse is hard* ^53^ ***HCP*** *For healthcare professionals:* *I came to the department after the project had started. I had some questions about how to use the system* ^46^ ***HCP***
Trust on healthcare service—F15 (F) *Pt*	Most participants reported that their primary motivation for using the digital PRO system was that the hospital had asked them to. Participants reported a high degree of trust in, and a good relationship with, the clinic—*You got to trust it. I think I do that, and it is only because I hope… this department has always been good…*^48^ ***Pt***
Healthcare professionals’ motivation: - Enhanced clinical documentation—F17 (F) *HCP* - Access to PRMs results—F17, F18 (B)(F) *HCP* - Age and length of service—F18 (B) *HCP*	*Enhanced documentation:* They found that the new ePSRM creates uniformity in how notes are generated^36^ ***HCP*** *Access to PRMs results:* *I tried to look but when I looked in EPR only her older scores were there. I didn’t have time to then go to try to look on the website before meeting with her* ^43^ ***HCP*** *Age and length of service:* *My guess is most younger physicians are probably going to be comfortable, but people who are towards the, end of their career probably won't be very open to making a whole lot of changes* ^36^ ***HCP***
Acceptance of digital system by older adults—F19 (F) *Adm*	*Really just encouraging them that if the patient is satisfied with this program then, patient satisfaction equals our satisfaction I believe* ^36^ ***Adm***
Location of PRMs completion—F20 (B)(F) *Pt*	Utilizing the computer at the clinic was susceptible to infection or hygiene risks and unprofessional behavior by other patients [..]^35^ ***Pt*** *It was probably more convenient at home to do it on your time, plus you could stop, come back, finish if somethin’ came up or somethin’ you had to do* ^47^ ***Pt***
Emotional experiences—F23 (F) *HCP*	The nurses believed that it may have been easier for the patients to initially complete the ePROs rather than talk about the items, especially for challenging topics such as depression, anxiety, sex, or pain—*maybe they're embarrassed a little bit or it's easier to put it on a screen maybe than talk about it?*^41^ ***HCP***
Patient—healthcare professional communication—F25: - Interactive conversations with electronic system (F) *HCP Pt* - Feels impersonal (B) *Pt*	*Interactive conversations with electronic system:* [..] the electronic PRM format lends itself to more interactive conversational sessions with patients. For example, physicians are able to view and review patients' responses before meeting with patients that enhances their interaction, spend less time on interview, and spend more time for comprehensive discussions on treatment plans with patients. It also improved feedback to patients. – ..*I used to draw pictures for the patient that there's like graphs and diagrams; I don't have to do that anymore*^36^ ***HCP*** Most participants identified that using digital PROs informed and enhanced their face-to-face consultations—*I guess it is to get the information, to be prepared and to get a holistic view—also backwards to see, if there is anything to see, when you get enough questionnaires filled out*^48^ ***Pt*** *Feels impersonal:* Some participants had concerns about being lost in the system after being assigned to the digital PRO system, therefore perceiving the digital PROs as a barrier to interaction with their healthcare providers—*It took a while before I got one [a PRO questionnaire]. It is almost… I think it is a year after we discussed it, that I got one. Why, I don’t know. But then again, they had not promised that it would be fast. But it took a long time. I did come to think I was forgotten*^48^ ***Pt***
Efficiencies, time constraints and changes to work routines for healthcare professionals—F26 (B)(F) *HCP Adm*	*It is a barrier that you have to log in to another system [AmbuFlex] if it does not substitute other tasks* ^46^ ***HCP*** *We type our notes and it used to take me for a complicated patient sometimes up to 40 minutes or so to write that lengthy report. Now it takes me about 18 minutes … all of that time that is saved is then used to actually talk to the patient* ^36^ ***HCP*** *the providers are able to do what they need to do quicker and get the patient back, versus waiting on them to fill out the paper, going over it, and then calling the patient back* ^36^ ***Adm***
Time consuming for older adults to complete PRMs electronically—F27 (B) HCP	Using OncoQuest takes too much time according to their patients^50^ ***HCP***
Change management—F30: - Flexibility in developing electronic system (F) *HCP* - Operational workflows (B)(F) *HCP Adm*	*Flexibility in developing electronic system:* *The other thing was the inherent flexibility … we could go through various iterations very quickly and that allowed us to get to a point where we were not afraid of experimenting* ^36^ ***HCP*** *Operational workflows:* *No, I didn’t log onto the website. It is too much trouble. I forgot that I could find the results in EPR* ^43^ ***HCP*** One continued maintenance aspect expressed by multiple participants focused on continued training. - *Consistent training on it so that we [are] always kept up to speed on what we have to do. Training new staff if they come in would maintain consistency across the board I believe*^36^ ***Adm***
Resources to manage collection and use of PRMs—F31: - Education to use electronic system (F) *Pt Adm* - Technical support (B)(F) *HCP Adm Pt* - IT infrastructure (B)(F) *HCP* - Funding (B)(F) *HCP*	*Education and technical support:* Patients that expressed difficulty with technology indicated that having formal instruction or someone to assist or engage them in the electronic communication could empower them to consider this avenue^40^ ***Pt*** *Technical support:* Insufficient staff for support and insufficient staff for coordination were reported as barriers^34^***HCP*** There were only minor technical challenges, and the patients were very compliant and contacted the department in case of any technical problems^44^ ***Pt*** *We have great help if we don't know what to do or how to do it. Someone's there to help us, so we know where to, who to reach out to* ^36^ ***Adm*** *IT infrastructure:* The availability of OncoQuest outcomes during consultation is hampered due to difficulties to trace OncoQuest results at their computer screen^50^ ***HCP*** *Funding:* Financial support extended to cover the cost of the e-PROM platform by the Ministry of Health was proposed as a facilitator^35^ ***HCP***

Abbreviations: EHR = electronic health record; EPR = electronic patient record; PRM = patient-reported measures.

Stakeholder perspectives indicated as ***Pt—***Older adults; ***HCP—***Healthcare professional; ***Adm—***Administrative staff and ***C*—**Caregivers.

#### Patient condition

The lack of access to digital technology, digital knowledge and skill gaps, and dexterity and visual impairment to using electronic devices were key *Opportunity* and *Capability* barriers specific to the electronic completion of PRMs among older adults. More broadly, visual and cognitive impairment (eg, memory loss), language difficulties, low literacy-associated reading difficulty and health knowledge gaps hindered older adults to complete PRMs in general.

#### Technology

Barriers and facilitators in this domain were specific to the electronic collection and use of PRMs. All factors except one (privacy and security of personal data) were related to *Opportunity*. Optimal design of the user interface was key to having older adults and healthcare professionals engage with digital technology to complete and use PRMs. Large font and screen size, and less clicks and scrolls were some of the features older adults preferred in the user interface. Unnecessary pop-up alerts, cumbersome drop-down menus and certain display graphics (eg, multiple colors, smiley faces) were some features disliked by older adults. Some features that both older adults and healthcare professionals considered important were clear labeling of options and responses, appropriate display graphics, intuitive interface allowing for easy progression of either the questionnaire or viewing of results and an optimal number of clicks to navigate the electronic system. Technical barriers, such as slow response times and loss of internet connectivity, were experienced by both older adults and healthcare professionals. A larger electronic device like a tablet or computer was preferred by most older adults over a smartphone. Privacy and security concerns with electronic completion of PRMs among older adults and healthcare professionals related to *Motivation*.

#### Value proposition

Older adults having the ability to remotely complete PRMs using the electronic system, typically from home was cited as a facilitator. However, some older adults feared that electronic completion of PRMs may replace face-to-face interactions with their healthcare professional. Healthcare professionals having easy and immediate access to PRMs data in electronic health records, and graphical visualization of patient problems was a key facilitator. Improved clinic efficiency through automated administration of PRMs and enhanced electronic health record documentation through the electronic capture of PRMs was a notable benefit. More broadly, patient—healthcare professional communication, appropriate timing and frequency for PRMs completion were facilitators for both older adults and healthcare professionals. Additionally, completion of PRMs was dependent on whether or not PRMs were utilized and informed care planning, provision of additional resources to address problem areas (eg, education or social services) and advanced quality of care and research. All factors in the value proposition domain were related to *Motivation*.

#### Adopter system

The knowledge and skills to use electronic systems for completion and analysis of PRMs was identified as both a barrier and facilitator among older adults and a barrier among healthcare professionals. Efficiencies and time constraints associated with using electronic systems to capture and analyze PRMs were identified by healthcare professionals and administrative staff as a barrier. Change resistance to engage with digital technology among healthcare professionals who were older and had longer service emerged as an additional barrier. Older adults receiving support from their social circle to use electronic systems to complete PRMs was a facilitator. More broadly, healthcare professionals lacking knowledge and skills to interpret PRMs, discrepancies in health assessment between older adults and healthcare professionals (e.g., symptoms not judged as severe as reported by older adults), and changes to existing work routines were identified as barriers. Social influences on older adults (ie, support to complete PRMs and support leading to suppression of autonomy), trust in healthcare provider, and emotional experiences (eg, distressed when answering questions about health, personal or sensitive topics) were other key factors identified in this review. Additionally, understanding the rationale for electronic completion and use of PRMs, with clear messaging of the benefits was salient for older adults and healthcare professionals. Questionnaire relevance, optimal design of questionnaire (eg, number of questions and simple language), and response capture options allowing for numerical ratings and free text fields were facilitators cited by older adults and healthcare professionals. Factors in the adopter system domain were related to *Capability*, *Motivation,* and *Opportunity.*

#### Organization

Resources to implement digital technology for PRMs administration highlighted the need for education on use of electronic system among users, technical support to troubleshoot technical errors, digital technology infrastructure enabling seamless data integration (eg, with electronic health records) and adequate funding to acquire and maintain the digital technology. Change management that supported clear workflows in the electronic system (eg, finding PRMs results in the electronic system and sending of PRMs information to healthcare professionals) and processes that allowed for multiple iterations of the electronic system facilitated the adoption of electronic collection and use of PRMs among healthcare professionals. More broadly, access to resources such as education to guide PRMs collection and use, adequate support (eg, support staff and reminders to support PRMs administration) and adequate staff to act on PRMs responses (eg, increasing workload with PRMs administration for healthcare professionals) were identified. Appropriate change management (eg, clear operational workflows for acting PRMs results, support and training), involvement of leadership and champions, and positive team culture were identified as facilitators to PRMs collection and use. All factors were linked to *Opportunity*.

#### Wider context

Access to government funding to acquire digital technology for PRMs collection was reported as a facilitator, and was unique to the electronic collection and use of PRMs in older adults’ care. More broadly, regulatory directives mandating PRMs completion was considered a barrier when clear messaging of purpose was absent. However, being mandated to collect PRMs under a regulatory requirement was considered a motivator by healthcare professionals to encourage their engagement in PRMs collection. Both factors were linked to *Opportunity*.

## Discussion

### Summary of the main findings

We synthesized 22 studies reporting the stakeholder perspectives of 374 older adults, 183 healthcare professionals, 3 caregivers, and 5 administrative staff on the barriers and facilitators influencing uptake of electronic collection and use of PRMs in older adults’ care. Thirty-four findings describing barriers and facilitators were derived and grouped into 11 thematic categories that included: (1) older adult’s characteristics; (2) digital technology; (3) support from social circle; (4) knowledge and skills; (5) motivation and incentives for capture and use of PRMs; (6) emotional experience; (7) older adults’ autonomy; (8) patient—healthcare professional communication; (9) workflow; (10) organizational factors; and (11) questionnaire selection and design. Most findings were graded as high (*n* = 3) or moderate (*n* = 26) confidence indicating end users such as patients, healthcare professionals, healthcare service administrators, and researchers can be generally confident in our findings. Furthermore, we mapped the findings to the theoretical domains of NASSS and COM-B frameworks to identify possible determinants of change which can inform the design of implementation interventions.

### Comparison with other reviews

A number of findings synthesized in this review are novel and not previously reported in reviews of factors influencing PRMs implementation more generally^54^^,^^55^ or electronic delivery of PRMs in various healthcare settings.^18^ These include older adults’ positive emotional experiences when using a digital interface to respond to questions on personal and sensitive topics, older adults’ and healthcare professionals’ privacy and security concerns with electronic completion of PRMs, easy to use electronic devices for PRMs completion among older adults, benefits of electronic systems for automated administration of PRMs and documentation in electronic patient records, and gaps in older adults’ health knowledge. Bi-directional factors (ie, serving as barriers or facilitators) include the location, timing and frequency of PRMs collection as well as the length, complexity and capture options for PRMs.

Our review highlights that aging-related and socio-economic challenges influence older adults’ ability to engage with digital technology and electronically complete PRMs. Our findings suggest that suboptimal design of digital technology for PRMs completion by older adults is likely to reflect a failure to adequately consider the specific characteristics and needs of older adults. Additionally, older adults’ access to digital technology (ie, electronic devices for questionnaire completion and internet access) is likely to influence their ability to complete the questionnaire independently and remotely. Older adults having access to digital technology appears to be associated with greater familiarity and skills to navigate through the digital devices to complete the questionnaire. This means that older adults are inherently likely to be adopters so long as issues of access, digital literacy and user interface are addressed.

Some of our findings are consistent with previous reviews. Namely, providing support for patients to complete PRMs, user-friendly digital technology, provision of services to address issues identified in PRMs responses, improving patient—healthcare professional communication, and change management that supports clear operational workflows and trialability of systems prior to implementation served as facilitators.^54^^,^^55^ Barriers identified in this review, consistent with prior reviews, include lack of appropriate IT infrastructure, lack of PRMs questionnaire relevance to patient health and clinical assessment, gaps in patients’ digital and language literacy to complete PRMs, and cognitive or physical impairments hindering PRMs completion.^18^^,^^54^^,^^55^

### Strengths and limitations

The strengths of this study include a comprehensive search strategy, grading of review findings and independent synthesis of findings by two authors. The use of the NASSS, a digital technology implementation framework, and COM-B, a behavior change framework, in this review produced a comprehensive set of explanations that could be important determinants of change in implementing electronic collection and use of PRMs in older adults’ care.

As a limitation of this study, no grey literature was searched which may have prevented additional relevant issues being considered. However, the comprehensive search strategy enabled the identification of a good proportion of studies to include in this review for an in-depth analysis of relevant factors. The search date for this review is a limitation, however, potentially eligible studies report no perspectives of stakeholders other than those already in this review. Most studies included in this review reported experiences of older adults and healthcare professionals. Other stakeholder perspectives including that of the caregivers and administrative staff appear to be under-reported. This suggests the need for primary qualitative studies to consider wider stakeholder views such as those of administrative staff and caregivers, in addition to those such as organization leaders, implementation specialists and digital technology developers. The generalizability of the findings in contexts other than those reported in this review may be limited to countries (eg, United States, United Kingdom, Canada, and Netherlands), healthcare settings (eg, oncology) and type of PRM (ie, PROMs). This calls for further research in various healthcare settings, countries and PREMs implementations. Additionally, no factors were mapped to the NASSS domain *embedding and adaptation over time*, indicating a need a for future research on the digital technology adaptability in changing context.

## Conclusion

We synthesized 22 studies that identified barriers and facilitators to the implementation of electronic collection and use of PRMs in older adults’ care from the perspective of older adults, caregivers, healthcare professionals and administrative staff. Through the mapping of these findings to NASSS and COM-B frameworks, a comprehensive set of explanations are provided to inform the design of future implementation efforts. It is important to address barriers associated with digital technology access, digital literacy and user interface, to increase the likelihood of older adults’ adopting digital technology to complete PRMs electronically. This review highlighted a lack of evidence on, and need for future research to explore, the perspectives of other stakeholders, such as organizational leaders, digital technology developers and implementation specialists; a knowledge gap on the adaptability of electronic collection and use of PRMs in changing healthcare contexts; and the need for more research exploring the factors influencing implementation of PREMs.

## Supplementary Material

ooae068_Supplementary_Data

## Data Availability

The data underlying this article are available in the article and in its online supplementary material.
