# Supplementary material for: Barriers and facilitators to the uptake of electronic collection and use of patient-reported measures in routine care of older adults: a systematic review with qualitative evidence synthesis
Source: JAMIA Open. 2024 Aug 2;7(3):ooae068. doi: 10.1093/jamiaopen/ooae068 (PMC11296862; doi:10.1093/jamiaopen/ooae068)
Supplement: ooae068_Supplementary_Data [file ooae068_supplementary_data.zip › ooae068_Supplementary_Data/Appendix 1 - Eligibility criteria.docx]

**Supplemental appendix 1 – PerSPecTIF question framework and eligibility criteria**

**PerSPecTIF question framework**

| **Per** | **S** | **P** | **E** | **(C)** | **Ti** | **F** |
| --- | --- | --- | --- | --- | --- | --- |
| **Perspective** | **Setting** | **Phenomenon of Interest/**  **Problem** | **Environment** | **Comparison (optional)** | **Time/Timing** | **Findings** |
| *From perspective of:*  Older adults aged 65 years and above  Caregivers of older adults  Healthcare professionals  Administrators  Researchers  Information Technology Professionals  Policy Makers | *In setting of:*  Healthcare including hospital, primary care, long term care, community care and residential aged care | How does the phenomenon of electronic collection of patient-reported measures in older adult care | Within environments, not limited to hospital, primary, long term, community care, home and work environments | - | During planning  During development (i.e. electronic collection system development, data integration, consumer/clinician interface development)  During implementation  During use: on admission, discharge, post discharge | Relate to perceptions (barriers and enablers) of:  Older adults aged 65 years and above  Caregivers of older adults  Healthcare professionals  Administrators  Researchers  Information Technology Professionals  Policy Makers |

**Study Inclusion/Exclusion Criteria:**

|  | ***Phenomenon of Interest*** | ***Target population receiving intervention*** | ***Setting and Environment*** | ***Type of Studies*** |
| --- | --- | --- | --- | --- |
| **Inclusion criteria** | - Intervention must include electronic collection of patient-reported measures in the routine care of older adults - Perspectives, views and experiences of patients and/or other related stakeholders (Caregivers of Patients, Healthcare professionals, Administrators, Researchers, Information Technology Professionals, Policy Makers) about barriers and enablers must be included - Stage of evaluation to include planning, development, implementation or use | - Older adults aged 65 years and above - When considering patient views, recruited sample should report participants aged 65 years and above - When considering other stakeholder views, study should report factors relating to older adults (even if age is not specified) in the results and discussion sections - Studies recruiting adults aged 18 years and above will be considered | - In healthcare setting(s) including hospital, primary care, long term care, community care and residential aged care, in any clinical specialty - Environment of electronic collection of patient-reported measures to include all environments not limited to hospital, home or work | - Primary studies that include qualitative or mixed method study designs, that report use of qualitative methods for both data collection and data analysis |
| **Exclusion criteria** | - Electronic questionnaires intended to capture clinician-reported outcomes and experiences, or other measures not related to patient-reported measures from age groups below 65 years - Studies that focus on intervention effectiveness only | - Paediatric patients receiving the intervention - No reporting of older adults in the results or discussion in studies reporting on perspectives of other stakeholders | - In clinical trial research setting | - Primary studies with quantitative study designs - Systematic reviews - Descriptive studies - Opinion papers, commentaries, editorials, dissertations - Unpublished studies - Policies - Guidelines - Study protocols |
