# Supplementary material for: Barriers and facilitators to the uptake of electronic collection and use of patient-reported measures in routine care of older adults: a systematic review with qualitative evidence synthesis
Source: JAMIA Open. 2024 Aug 2;7(3):ooae068. doi: 10.1093/jamiaopen/ooae068 (PMC11296862; doi:10.1093/jamiaopen/ooae068)
Supplement: ooae068_Supplementary_Data [file ooae068_supplementary_data.zip › ooae068_Supplementary_Data/Appendix 2 - Search Strategy.docx]

**Supplemental appendix 2 – Search string**

**MEDLINE** *(Ovid MEDLINE(R) and Epub Ahead of Print, In-Process, In-Data-Review & Other Non-Indexed Citations, Daily and Versions(R) <1946 to October 27, 2021>)*

1. Patient Reported Outcome Measures/
2. "Patient Reported Outcome* Measure*".ti,ab.
3. "Patient reported measure*".ti,ab.
4. PROM$1.ti,ab.
5. PREM$1.ti,ab.
6. "Patient reported outcome assessment".ti,ab.
7. Patient reported outcome*.ti,ab.
8. "Patient outcome assessment".ti,ab.
9. ePRO.ti,ab.
10. ePROM.ti,ab.
11. ePREM.ti,ab.
12. "electronic patient reported outcome measure*".ab,ti.
13. "electronic patient reported outcome*".ti,ab.
14. "patient facing electronic questionnaire*".ti,ab.
15. ((electronic or internet or digital or web or online) adj3 (survey or questionnaire)).ti,ab.
16. "Surveys and Questionnaires"/
17. "health care survey*".ti,ab.
18. "electronic reporting".mp.
19. barrier*.ti,ab.
20. Facilitator*.ti,ab.
21. enabler*.ti,ab.
22. belief*.ti,ab.
23. perception*.ti,ab.
24. perspective*.ti,ab.
25. view*.ti,ab.
26. preference*.ti,ab.
27. insight*.ti,ab.
28. experience*.ti,ab.
29. attitude*.ti,ab.
30. 1 or 2 or 3 or 4 or 5 or 6 or 7 or 8 or 9 or 10 or 11 or 12 or 13 or 14
31. 15 or 16 or 17 or 18
32. 19 or 20 or 21 or 22 or 23 or 24 or 25 or 26 or 27 or 28 or 29
33. 30 and 31 and 32
34. limit 33 to (english language and yr="2001 - 2021")

**Web of Science**

#1 - TS=("Patient Reported Outcome Measure*" OR "Patient Reported Measure*" OR "PROM" OR "PREM" OR "Patient reported outcome assessment" OR "Patient reported outcome*" OR "Patient outcome assessment" OR "ePRO" OR "ePROM" OR "ePREM" OR "electronic patient reported outcome measure*" OR "electronic patient reported outcome*" OR "patient facing electronic questionnaire")

#2 - TS=("Surveys" OR "Questionnaires" OR "Electronic reporting" OR "(electronic or internet or digital or web or online) adj3 (survey or questionnaire)" )

#3 - TS=("barrier*" OR "facilitator*" OR "enabler*" OR "belief*" OR "perception*" OR "perception*" OR "perspective*" OR "view*" OR "experience*" OR "insight*" OR "attitude*")

#4 - #1 AND #2 AND #3

***Filters applied:*** English Language, Years: 1 Jan 2001 – 27 Oct 2021

**CINAHL (EBSCO Host)**

1. (MH "Outcome Assessment") OR (MH "Patient-Reported Outcomes") OR (MH "Outcomes (Health Care)")
2. TI ( "patient-reported outcome* measures" or "prom" ) OR AB ("patient-reported outcome* measures" or "prom" )
3. TI ( "patient-reported experience measures" or "prem" ) OR AB ( "patient-reported experience measures" or "prem" )
4. TI "Patient-reported measure*" OR AB "Patient-reported measure*"
5. TI "Patient-reported outcome* assessment" OR AB "Patient-reported outcome* assessment"
6. TI "Patient outcome* assessment" OR AB "Patient outcome* assessment"
7. TI "electronic patientreported outcome* measure*" OR AB "electronic patientreported outcome* measure*"
8. TI ( "ePROM" or "ePRO" or "ePREM" ) OR AB ( "ePROM" or "ePRO" or "ePREM" )
9. TI "electronic patientreported outcome*" OR AB "electronic patientreported outcome*"
10. TI "patient facing electronic questionnaire*" OR AB "patient facing electronic questionnaire*"
11. TI ( (electronic or internet or digital or web or online) n3 (survey or questionnaire) ) OR AB ( (electronic or internet or digital or web or online) n3 (survey or questionnaire) )
12. TI ( "barrier*" OR "facilitator*" OR "enabler*" OR "belief*" OR "perception*" OR "perspective*" OR "view*" OR "preference*" OR "insight*" OR "experience*" OR "attitude*" ) OR AB ( "barrier*" OR "facilitator*" OR "enabler*" OR "belief*" OR "perception*" OR "perspective*" OR "view*" OR "preference*" OR "insight*" OR "experience*" OR "attitude*" )
13. TI "patient-reported outcome*" OR AB "patient-reported outcome*"
14. S1 OR S2 OR S3 OR S4 OR S5 OR S6 OR S7 OR S8 OR S9 OR S10 OR S13
15. S11 AND S12 AND S14

***Filters applied:*** English Language, Years: 1 Jan 2001 – 27 Oct 2021
