# Supplementary material for: Barriers and facilitators to the uptake of electronic collection and use of patient-reported measures in routine care of older adults: a systematic review with qualitative evidence synthesis
Source: JAMIA Open. 2024 Aug 2;7(3):ooae068. doi: 10.1093/jamiaopen/ooae068 (PMC11296862; doi:10.1093/jamiaopen/ooae068)
Supplement: ooae068_Supplementary_Data [file ooae068_supplementary_data.zip › ooae068_Supplementary_Data/Appendix 3_Additional study details_revised.pdf]

Supplemental appendix 3 – Additional details of included studies

Author and year	Brief description of electronic system features	Procedures for electronically collecting and reviewing patient-reported measures	Questionnaire(s) and no. of items administered electronically	Time taken to complete questionnaires electronically	Device used	Timing and location of	Who administered questionnaire electronically?	Patient's previous exposure to paper-based questionnaire completion	Patient characteristics associated with disease condition and other factors
Long et al (2021) [10]	-	▪ A link to PROM questionnaire emailed asking patient to complete prior to clinic visit<br>▪ Patients who do not complete them in advance are asked to complete them in the waiting area on their personal smartphone or on a clinic-owned tablet<br>▪ Patients facing difficulty are assisted by clinic staff by administering the PROM, reading loud and recording responses on patients' behalf<br>▪ For non-English-speaking patients, the staff used a live phone interpreter	-	-	▪ Smartphones<br>▪ Tablet computer<br>▪ Laptop computer<br>▪ Desktop computer	*Timing:*<br>Before clinic visit<br><br>*Location:*<br>▪ Clinic (waiting area and check-in area)<br>▪ Home	Self-administered by patient<br><br>Administered and completed by carers or clinic staff on behalf of patient	Patients had prior exposure to paper-based questionnaires<br><br>Patients complete paper- and electronic-based questionnaires in study	▪ Patients have upper extremity conditions with varying degrees of disability<br>▪ Physical challenges seen with completing PROM questionnaire (whether to write or use device)<br>▪ Vision and cognitive impairment assumed in some patients
Aiyegbusi et al (2018) [33]	▪ Paper-based questionnaire adapted for electronic collection<br>▪ Web-based questionnaire accessed through secure electronic patient portal<br>▪ User interface kept similar to original paper version<br>▪ No pull-down menus and double clicking<br>▪ Pages kept to a minimum<br>▪ Colour palette restricted<br>▪ Text on neutral background in black Arial font<br>▪ Demonstration version used to test system	▪ Minimal assistance provided to patients completing questionnaire<br>▪ Allowed family members to be present to simulate home environment<br>▪ Told to recall and report their health over the past 4 weeks for KDQOL-36 completion and within the last week for IPOS-Renal completion<br>11 tasks followed in test session (see Appendix)	Validated PROM questionnaires administered:<br>▪ Kidney disease quality of life-36 (KDQOL-36): 36 items<br>▪ Integrated Palliative Care Outcome Scale-Renal (IPOS-Renal): 11 items	Overall:<br>▪ Mean time: 15.9 minutes<br>▪ Range: 8 – 34 minutes<br><br>KDQOL-36:<br>▪ Mean time: 10 minutes<br>▪ Range: 5 – 20 minutes<br><br>IPOS-Renal: 11 items<br>▪ Mean time: 5.9 minutes<br>▪ Range: 3 – 14 minutes	Desktop computers	*Timing:*<br>Between clinic visits *(a priori scenario assumed)*<br><br>*Location:*<br>Home<br>*(Simulated in outpatient clinic)*	Self-administered by patient	Assumed to have prior exposure to completing paper-based PROM questionnaires	▪ Tend to be older adults<br>▪ Age-related physical and cognitive limitations assumed<br>▪ Fatigue and cognitive impairment assumed<br>▪ Patients with less experience on use of electronic devices and internet had highest number of errors in using system<br>▪ No patients with debilitating co-morbidities

									▪ 75% retired and of Caucasian origin (n = 6)<br>▪ All owned electronic devices and had access to internet
Amini et al (2021) [34]	▪ Data collection tool administering questionnaire was either linked to the EHR or accessed through web-based platform	▪ Treating healthcare professionals review PROMs and feedback in consultation room	-	-	-	*Timing:*<br>Before clinic visit	-	Unknown about whether patients have prior exposure to completing paper-based PROM questionnaires	▪ Language barriers experienced with patients with primary language other than Dutch
Kaur et al (2019) [35]	▪ Questionnaires accessed through web applications 'REDCap' and 'TickiT'<br>▪ REDCap interface was dual tone and listed all questions on one page<br>▪ TickiT platform had flexible design options. Included one question per screen, with different response options (emoticons, checkered boxes or circles), and varied colour palette	▪ Patients were shown 3 formats of a PROM scale relevant to the patient's procedure<br>▪ Formats included a paper version and 2 different web applications (REDCap and TickiT) for PROMs collection	Validated PROM (4 response options on satisfaction) questionnaire relevant to procedure:<br>▪ BODY-Q Satisfaction with Body scale: 10 items<br>▪ BREAST-Q Augmentation Module Satisfaction with Breast scale: 16 items<br>▪ FACE-Q Satisfaction with Facial Appearance scale: 10 items	-	▪ Tablet computer (iPad)<br>▪ Laptop computer (Macbook)<br>▪ Smartphone<br>▪ Desktop computer	*Location:*<br>▪ Clinic *(consultation room)*<br>▪ Home	-	Assumed to have prior exposure to completing paper-based PROM questionnaires before study.<br><br>Patients complete paper- and electronic-based questionnaires in study	▪ Fluent in English<br>▪ No cognitive impairment<br>▪ Patients recruited may have been pre- or post-treatment<br>▪ 82% have university education (n = 9)<br>▪ All owned one or combination of electronic device(s) and had access to internet
Spaulding et al (2019) [36]	▪ Flexible technology platform through external vendor used for electronic data capture<br>▪ Computer adaptive testing and branching logic was used to individualise the questionnaire set<br>▪ Questions presented one at a time<br>▪ Visual display of trends available for physicians	▪ System explained to patient by front desk staff (if necessary)<br>▪ Treating healthcare professionals review responses before and during face-to-face visit<br>▪ Healthcare professionals use visual display of responses on iPad screens to discuss symptoms with patients	Validated PROMs questionnaire set included 8 questionnaires<br><br>**All patients must complete:**<br>▪ Patient health questionnaire (PHQ-9)<br>▪ Generalized anxiety disorder (GAD-7)<br>▪ World Health Organization Disability Assessment 2.0 (WHODAS 2.0)<br><br>**Those screened positive must complete:**	-	Tablet computer (iPad)	*Timing:*<br>Before clinic visit<br><br>*Location:*<br>Clinic *(waiting room)*	Front desk staff	Patients have prior exposure to completing paper-based PROM questionnaires	▪ Patient populations from care areas of transplant, cancer, cardiovascular issues, chronic pain, complex neuropsychiatric disorders, complex care from primary care, and other specialty and subspecialty areas presented

			▪ Obsessive-compulsive inventory (OCI-R)<br>▪ Panic disorder severity scale (PDSS-SR)<br>▪ Social phobia inventory (SPIN)<br>▪ Mobility inventory for agoraphobia (MIA)<br><br>**Optional:**<br>▪ Impact of event scale revised for post-traumatic stress disorder (IES-R)						▪ 26.5% have high school education or less and 22% retired<br>▪ 76% of Caucasian origin<br>▪ Likely to have cognitive, intellectual, visual impairment, developmental disability, etc.
Delgado-Herrera et al (2017) [37]	▪ Mobile application accessed on mobile platforms (Android and iOS), able to translate questionnaire into 10 languages<br>▪ Mobile application sends daily notifications via a pop-up screen to users to encourage entering of data in a timely manner<br>▪ Instructions displayed at start of both questionnaires<br>▪ Reminder generated by mobile application if data is not entered in the IBS-D Symptom Event Log within 24 hours<br>▪ Pop-up messages appeared to guide through questionnaire completion<br>▪ Report history section aggregate data for time periods of 1 day, 1 week, 1 month and 3 months (displayed in graphic and summary format)<br>▪ Data programmed into device for study purposes. However, option available for results to be shared with clinician via email	▪ Participants asked to download the mobile application onto their electronic device. Assistance provided from the researcher if required<br>▪ In case of difficulties experienced in downloading and installing mobile application, a backup device (Anroid or iOS) was provided by researchers<br>▪ Participants asked to interpret graphic representations and summary results of sample data (not their own) in report history	Validated Irritable bowel syndrome with diarrhea predominant symptoms (IBS-D) PROM questionnaire set consists of:<br>▪ IBS-D Daily Symptom Diary: 6 items<br>▪ IBS-D Symptom Event Log: 4 items	-	▪ Mobile device (Android)<br>▪ Mobile device (iOS)<br>▪ Laptop computer (Macbook)<br>▪ Smartphone	*Timing:*<br>Daily<br><br>*Location:*<br>Unclear	Self-administered by patient (assistance provided by researcher if required)	Patients have prior exposure to completing paper-based PROM questionnaires before study.<br><br>Patients complete paper- and electronic-based questionnaires in study	▪ Patients recruited considered themselves in either 'very good' or 'good' health<br>▪ More than half reported no co-morbidities<br>▪ 64% of Caucasian origin<br>▪ 48% have some college/high school education or less<br>▪ 12% retired
Mou et al (2021) [38]	▪ Questionnaires administered through EHR and accessible through both	▪ Questionnaire available at the time of visit and within online portal a week before	Primary Care Screening Bundle (PCSB) is a standardised 70-item	-	▪ Tablet computer (iPad)	*Timing:*<br>▪ Before clinic visit	Self-administered by patient	Patients had prior exposure to paper-based	▪ Patients presented from

	a digital patient portal and tablets in clinic<br>▪ Results are available real-time for review within the patient chart	the visit for completion by patient<br>▪ Immediately on completion, results are available for the clinician to use in the EHR<br>▪ Physicians prompt patients to complete questionnaire	questionnaire and consists of validated questionnaires:<br>▪ PHQ-2 for depression screening<br>▪ General Anxiety Disorder (GAD) for anxiety screening<br>▪ Drug use screening test<br>▪ Social Determinants of Health (SDOH) screening [Not PRO]<br>▪ Medicare Health Risk Assessment (HRA) questionnaire (for assessment of daily living activities, home safety and cognition) [Not PRO]			(Intended for annual and new patient visits)<br><br>*Location:*<br>▪ Clinic *(waiting room)*<br>▪ Home<br>▪ Other remote location	Unclear if clinic staff administers questionnaire at the time of visit	questionnaires completed in the waiting room prior to a visit.<br><br>Professionals follow manual workflow followed to scan and transcribe data into EHR, impeding ability to perform data analysis	multiple clinical domains<br>▪ 74% patients successfully completed PCSB electronically
Krawczyk et al (2019) [39]	▪ Electronic tablet-based tool (Quality of Life Assessment and Practice Support System) used for questionnaire administration<br>▪ Implemented in both Outpatient (community-based) and Inpatient (hospital-based) units<br>▪ Responses are immediately summarized and presented back to health care professionals	▪ All clinicians (including those in administrative or managerial positions) were eligible to participate and were offered the opportunity to use the QPSS with consenting patients and family members	Validated PROM questionnaires administered:<br>▪ Edmonton Symptom Assessment System–Revised Version (ESAS-r): *mandated use established*<br>▪ McGill Quality of Life–Revised Version<br>▪ Canadian Health Care Evaluation Project Lite Questionnaire	-	▪ Tablet computer	Timing:<br>▪ During admission<br><br>*Location:*<br>▪ Inpatient palliative unit	Healthcare professionals	Assumed that patients may have prior knowledge about paper-based PROM questionnaires, and that healthcare professionals administer these questionnaires and complete on behalf of patients	▪ Patients presented from multiple clinical domains
Navarro-Millán et al (2019) [40]	▪ No system implemented<br>▪ Preferences for system features recorded in supplementary information – Table 5	-	-	-	-	*Timing:* Between clinic visits<br><br>*Location:* Intended for Home	Intended for self-administration by patient	Patients had prior exposure to paper-based questionnaires completed every 2 – 3 months during follow-up visits	▪ Arthritis-related symptoms, functional impairment, conditions such as fibromyalgia and depression assumed<br>▪ Disease conditions impact physical function, social/work-related duties and fatigue

									▪ 52% of African American origin<br>▪ Disease duration was approximately 10 years<br>▪ 58% were 'very' or 'extremely' likely to use electronic/online tools for tracking and reporting about their health
Schick-Makaroff and Molzahn, (2017) [41]	▪ Two questionnaires (ESAS-r:Renal and satisfaction questionnaire) administered on mobile application using Filemaker Go iPad app (iOS7)<br>▪ KDQOL Complete (provided by Medical Education Institute) administered using web application	▪ Participants were asked to arrive 10–15 min early to take part in study<br>▪ Research assistant showed patient how to use the tablet computer (iPad) to complete questionnaire electronically. No other assistance was provided in completing the measures<br>▪ After completing all three questionnaires, results were printed that displayed graphically depicted scores<br>▪ Nurses were instructed to review results (scores) with patients. They had received education on use of electronically collected patient-reported measures<br>▪ Patients were provided with their own results and patient education material from KDQOL-Complete platform if requested<br>▪ Patients completed the Client Questionnaire on the tablet computer at the end of their appointment	Validated patient-reported measures questionnaires administered:<br>▪ Edmonton Symptom Assessment System for renal patients (ESAS-r:Renal)<br>▪ Kidney Disease Quality of Life-36 (KDQOL-36): 36 items<br>▪ Comox Valley Nursing Centre Client questionnaire (satisfaction questionnaire): 14 items	-	▪ Tablet computer (iPad)	*Timing:*<br>(typically every once in 3 months)<br>▪ Before clinic visit (PROMs)<br>▪ At end of consultation (satisfaction questionnaire)<br><br>*Location:*<br>▪ Clinic (waiting area)	Self-administered by patient	Patients had prior exposure to paper-based questionnaires completed every 3 months during clinic visits	▪ Experiencing of multiple symptoms, and impairments assumed<br>▪ Patients experiencing lack of English proficiency, inability to read, moderate to severe cognitive impairments or medical crisis were excluded<br>▪ 83% of Caucasian origin<br>▪ 68% retired<br>▪ 42% reported previous tablet computer use
Brochmann et al (2016) [42]	▪ Questionnaire accessed through internet-based tool, through an encrypted link to a secure server. This tool enables the capture and assessment of PROMs data	*Patient:*<br>▪ A paper tutorial is handed out to each patient before the questionnaire collection starts, to inform how the tool works	Validated PROM questionnaires administered:<br>▪ European Organisation for Research and Treatment of Cancer Quality of Life	▪ Majority of participants completed questionnaires in 10 – 20 minutes	-	*Timing:*<br>Once a month<br><br>*Location:*<br>▪ Home	Self-administered by patient	Patients had prior exposure to paper-based questionnaires	▪ Chronic conditions associated with heterogeneous haematological diseases

	▪ Questionnaires are completed and submitted one at a time<br>▪ Pop-up window to inform patient to complete all questions<br>▪ Final page contains text box with thank you note from healthcare professional, and providing additional relevant information<br>▪ Patient unable to review answers submitted<br>▪ Healthcare professionals are able to view the results through a log-in to the tool<br>▪ Flexibility in options for dispatch of questionnaire:<br>• Multiple formats (e.g. responses to predetermined combination of questionnaires or responses to one questionnaire at a time)<br>• Ability to select interval of questionnaire dispatch (e.g. once, once a week, once a month and every 3 months)<br>• Notifications on status of questionnaire dispatch and completion sent	▪ Depending on the patient's preference, an SMS and/or email request with a password and an encrypted link to a secure server is dispatched when it is time to complete the questionnaires<br>▪ Reminders are sent on day 2 if questionnaire is not submitted on day 1<br><br>*Healthcare professional:*<br>▪ A tutorial is available to the healthcare professional on the login page and as a paper tutorial handout, to inform about how to use the tool<br>▪ A username and password are required to login	Questionnaire-Core 30 (EORTC QLQ-C30)<br>▪ Myeloproliferative Neoplasm Symptom Assessment Form (MPN-SAF)<br>▪ Brief Fatigue Inventory (BFI)<br>▪ Short Form 36 Health Survey (SF-36)	(for all 4 questionnaires)				Patients complete paper- (9%) and electronic- (91%) based questionnaires in study	▪ Associated with a decreased lifespan<br>▪ Experience fatigue, fever/sweats and weight loss. Many suffer from cardiovascular and thromboembolic complications
Snyder et al (2013) [43]	▪ Questionnaire accessed through a web link<br>▪ Results from patient's current and previous questionnaires are displayed graphically, and data is imported into the EHR<br>▪ Free text box available for patients to report the issue of most concern that	▪ 3 days prior to target completion date, an email reminder is sent to patients with a link to the website to complete the questionnaire<br>▪ Clinicians received individual training on the system and accessing results via the system and via EHR. One page summary of questionnaires, their content	Validated PROM questionnaires administered:<br><br>*All patients (breast and prostate cancer) completed:*<br>▪ Version 1 short forms for six Patient Reported Outcomes Measurement Information System (PROMIS) domains: physical function, pain interference, satisfaction	PROMIS questionnaire:<br>▪ Median: 6 minutes (breast cancer patients) and 5 minutes (prostate cancer patients)<br>▪ Range: 2 – 12 minutes<br>▪ PROMIS questionnaires	▪ Laptop computer (in clinic)	*Timing:*<br>Every 2 weeks regardless of visit frequency<br><br>*Location:*<br>▪ Clinic<br>▪ Home or other remote location *(87%)*	Self-administered by patient at home or outside the clinic setting<br><br>Unclear on who administered questionnaire in clinic	Unknown whether patients had prior exposure to completing paper-based PROM questionnaires	▪ Patients in any stage of cancer, currently receiving oncology treatment were enrolled<br>▪ Required patients to be proficient in English, and physically and cognitively be

	requires discussion with clinician<br>▪ System reminds patients not to report on issues requiring immediate attention, although the capability to generate an automatic page or email to clinicians if such issues are reported	and interpretation of scores was provided<br>▪ Research coordinator trained patients on website and assigned username and a password to access questionnaire<br>▪ 3 days after target completion date, window for questionnaire completion was not accessible<br>▪ In case of timely completion by patient, research coordinator offered them option to complete questionnaire in clinic<br>▪ Clinicians were alerted by research coordinator on results availability<br>▪ Clinicians reviewed results in EHR for 51% of patients. Reviewed hard copy of results for 17% of patients, and remaining 32% was either reviewed in the system or not at all	with social roles, fatigue, anxiety, and depression<br><br>*Breast cancer patients completed*:<br>▪ European Organisation for Research and Treatment of Cancer Quality of Life Questionnaire (BR23)<br><br>*Prostate cancer patients completed:*<br>▪ Expanded Prostate Cancer Index Composite (EPIC) short form	completed at home were more likely to have no missing items (84%) compared to PROMIS questionnaires completed in the clinic (67%)<br><br>BR23 questionnaire:<br>▪ Median: 3 minutes<br>▪ Range: 1 – 11 minutes<br><br>EPIC questionnaire:<br>▪ Median: 4 minutes<br>▪ Range: 2 – 6 minutes					able to complete questionnaires<br>▪ Patients were not required to have a computer with internet access and had the option of completing their surveys using a laptop computer in the clinic prior to their visits<br>▪ Majority were white and 30% had some college or less education<br>▪ 79% had regular computer use
Tolstrup et al (2020) [44]	▪ Questionnaire accessed using software platform AmbuFlex to self-report symptoms and side-effects to immunotherapy<br>▪ Alert mechanism enabled for contacting Department for symptoms/side effects likely to worsen<br>▪ Real-time results availability for clinicians to access (No alert to clinician to notify of result availability)	▪ Tablet provided by clinic to take home to complete questionnaire<br>▪ Patients asked to choose fixed weekday for completing questionnaire (no reminders sent)<br>▪ Clinicians only viewed results upon patient presentation in clinic	Validated PROM questionnaires administered:<br>▪ Patient Reported-Outcomes version of the Common Terminology Criteria for Adverse Events (PRO-CTCAE)	-	Tablet computer with SIM card that required internet access	*Timing:*<br>Every week (continued for 24 weeks)<br>▪ Before clinic visit<br>▪ Patients had reported between 6 – 24 times (weeks)<br>▪ Majority reported more than 15 times<br><br>*Location:*<br>Home	Self-administered by patient at home or outside the clinic setting	Unknown whether patients had prior exposure to completing paper-based PROM questionnaires	▪ Patients undergoing immunotherapy and side effects associated with this form of cancer therapy can be severe and unpredictable<br>▪ Untreated toxicities may become life threatening in patients receiving immunotherapy
Yamada et al (2020) [45]	▪ Questionnaire accessed using app on mobile device	▪ Patient asked to bring own smartphone<br>▪ Link to questionnaire emailed at start of session	Point of care clinic decision support system patient-reported questionnaire administered:	Questionnaire takes 5 – 10 minutes to complete	▪ Smartphone (blackberry)<br>▪ Tablet	*Timing:*<br>▪ Before clinic visit *(intended for*	Self-administered by patient in clinic	Unknown whether patients had prior exposure	▪ Asthma is a common chronic disease that report gaps

	▪ Results transmitted to clinician clinical decision support system	▪ Functions, role and workflow of questionnaire including information transfer to clinician explained to patient<br>▪ Patients asked to download app on smartphone or tablet<br>▪ Patient asked to complete questionnaire independently on their device	▪ Electronic Asthma Management System questionnaire (intends to capture asthma-related parameters, using Canadian guideline-recommended symptom-based criteria)			*one week prior to doctor's appointment)*<br><br>*Location:*<br>▪ Home or other remote location<br>▪ Clinic (waiting area)		to completing paper-based PROM questionnaires	between guideline-recommended and real-world care<br>▪ Majority (83%) of patients participating had college education or higher<br>▪ All participants used their smartphone for daily tasks
Baeksted et al (2017) [46]	▪ Questionnaire accessed on tablet computer using software platform AmbuFlex<br>▪ Free text data capture fields available in questionnaire to report symptoms not covered by PROM questionnaire items<br>▪ Results made available as graphic real-time presentation (bar chart showing scores with assorted colour, lengths and numbers) to oncologists via AmbuFlex dashboard<br>▪ Graphics presentation showing change over time was possible<br>▪ AmbuFlex software integrated into clinician cancer therapy ordering system	▪ Patients given 5 – 10 minutes tutorial on completing questionnaire on tablet computer<br>▪ Unique personal identifier used as log-in to access questionnaire on the tablet computer<br>▪ Support was provided by on-site researcher in clinic as needed<br>▪ Oncologists received 5 – 10 tutorial about navigating the AmbuFlex software, and access/interpret results<br>▪ Oncologists encouraged to view results prior to or during patient appointment<br>▪ During weekly clinic meetings, study progress and logistical issues associated with workflow addressed	Validated PROM questionnaires administered:<br>▪ Patient Reported-Outcomes version of the Common Terminology Criteria for Adverse Events (PRO-CTCAE): 41 items	▪ Mean time: 6 minutes, 48 seconds<br>▪ Range: 3 minutes, 3 seconds – 46 minutes, 24 seconds	Tablet computer	*Timing:*<br>Every third week<br>▪ At each clinic visit, prior to consultation with oncologist<br><br>*Location:*<br>▪ Clinic	Self-administered by patient in clinic (Help received from relatives or researcher as needed)	Unknown whether patients had prior exposure to completing paper-based PROM questionnaires	▪ Patients undergoing cancer therapy and side effects associated with this form of cancer therapy need to be monitored<br>▪ Untreated toxicities may become life threatening in patients receiving cancer therapy<br>▪ Male patients being treated for castration-resistant metastatic prostate cancer, who were able to read, write, and speak Danish, and who were receiving chemotherapy participated in study<br>▪ 52% of questionnaire completions

									reviewed by oncologist<br>▪ From patients completing questionnaire, 66% had access to mobile device at home and 37% had high school or vocational education<br>▪ 23% needed help using the tablet computer
Samuel et al (2020) [47]	▪ Questionnaires accessed through web-based platform and automated telephone interface, administered using data collection system (PRO Core)<br>▪ Symptom summary report received by patient and clinician during clinic visit	▪ Questionnaires administered pre-treatment<br>▪ Training on questionnaire completion provided by clinical research assistant (at defined intervals of 1- and 3-months following treatment initiation)<br>▪ Participant could choose between completing questionnaire from home or clinic	Validated PROM questionnaires administered (approximately 45 symptom items in total):<br>▪ Patient Reported Outcomes Measurement Information System (PROMIS) short forms<br>▪ Bladder Cancer Index (BCI)<br>▪ Expanded Prostate Cancer Index Composite (EPIC)	-	-	Loc*ation:*<br>▪ Clinic (waiting area)<br>▪ Home	Unclear	Patients had prior exposure to paper-based questionnaires	▪ Patients undergoing cancer therapy experience wide range of symptoms<br>▪ Racial disparities (black patients in comparison to white patients) exist, which impact inequalities in health-related quality of life, treatment adherence and survival<br>▪ Participants had to be able to read, speak English and were cognitively able<br>▪ 38% of were Black participants, and majority had high school education and 60% preferred web-based system for questionnaire completion

									▪ 62% were White participants, and 61% had some college or higher education and 86% preferred a web-based system for questionnaire completion
Nielsen et al (2021) [48]	▪ Questionnaire accessed using software platform AmbuFlex	-	-	-	▪ Smartphone<br>▪ Computer	*Location:*<br>▪ Clinic<br>▪ Home	Unclear	Unknown whether patients had prior exposure to completing paper-based PROM questionnaires	▪ Patients with Inflammatory Bowel Disease (IBD) experience lifelong gastrointestinal disorders<br>▪ Disease patterns and severity vary in patients experiencing symptoms, with stress and depression symptoms worsening IBD<br>▪ Self-management is thought to improve IBD symptoms<br>▪ 12.5% of participants in age category of 65 years and above
Lehmann et al (2021) [49]	▪ Questionnaire administered using electronic system 'Computer-based Health Evaluation System (CHES)<br>▪ Patients are able to complete questionnaires in hospital (through CHES nurse survey interface) or home (through CHES patient portal interface)<br>▪ Healthcare professionals able to view patient	▪ Patients introduced to the portal by facilitator responsible for motivating patients and healthcare professionals about reporting and using PROM data at hospital<br>▪ Patients are given instructions for use and their personal log-in data for accessing the portal at home by the facilitator	Validated PROM questionnaires administered:<br><br>*All patients (multiple myeloma and chronic lymphocytic leukemia) completed:*<br>▪ European Organisation for Research and Treatment of Cancer Quality of Life Questionnaire-Core 30 (EORTC QLQ-C30)	-	Computer	*Timing:*<br>A week before clinic visit (visits range between 1 week and 12 months)<br>▪ Before clinic visit<br><br>*Location:*<br>▪ Clinic	Self-administered by patient in clinic (Help received from facilitator)	Unknown whether patients had prior exposure to completing paper-based PROM questionnaires	▪ Patients included in study experience multiple myeloma and chronic lymphocytic leukemia

	reported data (through CHES main interface)<br>▪ Results displayed graphically, and colour-coded for prioritisation of clinical evaluation<br>▪ Data reported within 7 days of clinic appointment are linked to the clinical data in the Austrian Myeloma Registry<br>▪ If patient reports clinically important result, patients directed to self-management tools<br>▪ Reminders sent to patients asking to contact clinical team in case of severe impairment	▪ Patients are advised to complete questionnaires electronically in the week before their hospital visits<br>▪ Patients are encouraged to complete questionnaires as often as they like, even daily if needed	*Multiple myeloma patients completed:*<br>▪ European Organisation for Research and Treatment of Cancer Quality of Life Questionnaire-Multiple Myeloma Module (EORTC QLQ-C30)<br><br>*Chronic lymphocytic leukemia patients completed:*<br>▪ European Organisation for Research and Treatment of Cancer Quality of Life Questionnaire - Chronic lymphocytic leukemia Module (EORTC QLQ-CLL 17)			▪ Home			
Duman-Lubberding et al (2017) [50]	▪ Questionnaire administered using computer-assisted system (OncoQuest) through dedicated touch screen computers<br>▪ Results are available real-time in graphical format for clinician use through a stand-alone application (OncoQuest viewer), viewed on a computer screen<br>▪ Results are linked to the hospital information system	-	Validated PROM questionnaires administered (79 items in total):<br>▪ European Organisation for Research and Treatment of Cancer Quality of Life Questionnaire-Core 30 (EORTC QLQ-C30)<br>▪ European Organisation for Research and Treatment of Cancer Quality of Life Questionnaire – Head and Neck Cancer (QLQ-H&N35)<br>▪ Hospital Anxiety and Depression Scale (HADS)	-	Touch screen computers	*Timing:*<br>▪ After follow-up visit with surgeon<br><br>*Location:*<br>▪ Clinic (separate consultation room)	Unclear	Unknown whether patients had prior exposure to completing paper-based PROM questionnaires	▪ Patients of varying tumor stage present, including those of advanced stage<br>▪ Patients undergo various treatments including surgeries and chemotherapy<br>▪ Usage rate of OncoQuest in the first year after implementation was 67% among head and neck cancer patients<br>▪ Use of system was associated with disease severity (e.g. patients treated for stage 1 and above used the system more often)

Grossman et al (2018) [51]	▪ Questionnaire administered on external system (mi.Symptoms), that links to the EHR directly<br>▪ Mock-up displayed sample PRO questions and results page (no visualisations)	▪ Research coordinator guided patient and healthcare professionals through mock-up of system<br>▪ Expected for clinic receptionist enters patient's medical record number into the PROMs system installed on iPad, that enables accessing of questionnaire	Validated PROM questionnaires administered:<br>▪ Patient Reported Outcomes Measurement Information System (PROMIS)<br>▪ Heart Failure Somatic Perception Scale (HFSPS)	-	Tablet computer (iPad)	*Timing:*<br>▪ Unclear<br><br>*Location:*<br>▪ Clinic (waiting room)	Administered by clinic receptionist	Unknown whether patients had prior exposure to completing paper-based PROM questionnaires	▪ Patients with confirmed heart failure diagnosis included<br><br>*Stage 1 (Value and challenges assessment):*<br>▪ 69% of patients look up health information online<br>▪ 46% Black or African American<br>▪ 31% have high school education<br><br>*Stage 3 (usability assessment):*<br>▪ 83% of patients look up health information online<br>▪ 58% White<br>▪ 42% have some school education
Moradian et al (2018) [52]	▪ Questionnaire administered using mobile phone-based program (ASyMs)<br>▪ Patients immediately receive evidence-based self-care advice upon the completion of questionnaire<br>▪ Results (meeting threshold criteria) alert clinicians to view results on webpage to initiate clinical intervention	▪ During usability testing procedure, participants were requested to follow tasks provided on mobile device	PROM questionnaires administered:<br>▪ e-symptoms PROM questionnaire to assess occurrence, severity and distress associated with each symptom	-	Mobile phone (Android)	*Timing:*<br>▪ Unclear<br><br>*Location:*<br>▪ Clinic (waiting room)	Self-administered by patient	Unknown whether patients had prior exposure to completing paper-based PROM questionnaires	▪ Patients undergoing cancer therapy and side effects associated with therapy need to be monitored<br>▪ Patients experiencing colorectal or lymphoma cancer types included<br>▪ 80% had higher education<br>▪ 70% had access to smartphone and 60% were comfortable

									using these devices<br>▪ 80% were comfortable using the internet
Sandhu et al (2020) [53]	▪ Questionnaire administered through patient portal (MyChart)<br>▪ Results available for oncologists view in the EHR. Various visualisation options exist	-	-	-	▪ Smartphone<br>▪ Computer	▪ Clinic		Patients had prior exposure to paper-based questionnaires	▪ Patients with different forms of cancer are treated by the oncologists interviewed for this study

Table created by the authors. EHR, Electronic Health Record; PRO, patient-reported outcome; PROM, patient-reported outcome measure, PROM
