# Supplementary material for: Barriers and facilitators to the uptake of electronic collection and use of patient-reported measures in routine care of older adults: a systematic review with qualitative evidence synthesis
Source: JAMIA Open. 2024 Aug 2;7(3):ooae068. doi: 10.1093/jamiaopen/ooae068 (PMC11296862; doi:10.1093/jamiaopen/ooae068)
Supplement: ooae068_Supplementary_Data [file ooae068_supplementary_data.zip › ooae068_Supplementary_Data/Appendix 4 - CASP assessment_references revised.pdf]

Supplemental appendix 4 – Quality appraisal of included studies

**Reference**	Was there a clear statement of the aims of the research?	Is a qualitative methodology appropriate?	Was the research design appropriate to address the aims of the research?	Was the recruitment strategy appropriate to the aims of the research?	Was the data collected in a way that addressed the research issue?	Has the relationship between researcher and participants been adequately considered?	Have ethical issues been taken into consideration?	Was the data analysis sufficiently rigorous?	Is there a clear statement of findings?	Overall assessment of methodology limitations*
**Long et al (2021)** [10]	Yes The aim of the study is clearly reported in the abstract and introduction of the paper. Researchers provided rationale as to why the study was important to undertake	Yes The qualitative approach was appropriate to explore patients views on barriers and facilitators to reporting PROMs electronically	Yes Appropriate detail on study design and justification for the choice of the interviews discussed	Yes Adequate detail provided on participant selection, appropriateness of study sample and invitation procedures. No detail on participants declining invitation	Yes Detail provided on choice of method and process of collecting data. Partial discussion of data saturation	Yes The researcher's role during data collection, sample recruitment and choice of location was discussed	Yes The study was approved by local ethics bodies. Detail on obtaining informed consent provided	Yes Appropriate detail on deriving themes reported. Partial description of the role of researcher, potential bias and data selection for presentation provided	Yes The findings were presented clearly in relation to the research aim. Data analysis rigour discussed and thorough. Study limitations have been considered	None
**Aiyegbusi et al (2018)** [33]	Yes The aim of the study is clearly reported. Researchers provided rationale as to why the study was important to undertake. The relevance of the study within PROMs research at an individual and broader level was explained	Yes The qualitative approach considered appropriate to explore participant views from usability testing	No The researchers employed interviews and observations as qualitative methods to address study's aim. Although a qualitative approach seemed appropriate, no justification for the choice of the methods was provided	Yes Participant selection was clearly explained. Explanation provided on purposeful sampling strategy and appropriateness of sample involved in study. No discussion on participants declining invitation	No Setting clearly described. Minimal explanation provided on how interviews were conducted. No detail on the use of interview topic guide and data saturation was not discussed	Yes The researcher's role during data collection, sample recruitment and choice of location was discussed	Yes The study was approved by the regional and local research ethics committees. Informed consent was obtained by all participants. Information on providing explanation to potential participants provided. Study data was anonymised	No In-depth description of the analysis process was lacking. Unclear about the considerations of credibility, transferability and reliability in study. The researcher's role, potential bias and influence in data analysis were not reported	Yes Clear reporting of findings described in relation to research aim. No explanation about the credibility of the findings provided, such as the inclusion of two or multiple researchers in the synthesis of findings. Study limitations have been considered	Major
**Amini et al (2021)**	Yes	Yes	Yes	No	Yes	No	Yes	No	Yes	Major

[34]	Study aim stated in the abstract and introduction is the same. Research aim phrased slightly different in the discussion, limiting to views of healthcare professionals, while abstract and introduction stated views of healthcare professionals and researchers. Significance of the study was discussed	The qualitative approach considered appropriate, given the aim to understand views of the healthcare professionals and researchers on barriers and enablers to implementing electronically integrated PROMs in the organisation	Appropriate detail on study design and justification for the choice of the open-ended survey was discussed	Partial explanation about study setting, participant selection and invitation provided. No explanation on recruitment process was provided and any participants declining invitation	Detail provided for choice of method and the process of data collection. Detail on pilot testing of the questionnaire and carrying out minor textual amendments had been discussed. Saturation of data was not discussed	No adequate detail about researcher's role, potential bias and influence during data collection, recruitment or choice of research sites provided	Ethical approval deemed unnecessary as this had been considered part of a quality improvement project. Informed consent was obtained by all participants and stated confidentiality was ensured	In-depth description of the analysis process was lacking. Unclear about the considerations of credibility, transferability and reliability in study. The researcher's role, potential bias and influence in data analysis were not reported	Clear reporting of findings described in relation to research aim. Data analysis rigour discussed. Study limitations have been considered	
**Kaur et al (2019)** [35]	Yes<br>The aim of the study is clearly reported in the abstract and introduction. Researchers provided rationale as to why the study was important to undertake	Yes<br>The qualitative methodology is deemed appropriate, given the study aim to identify barriers and facilitators to collecting PROMs from patient’s perspective	Yes<br>No adequate explanation on reasons for employing semi-structured interviews	No<br>No discussion provided on selection of participants. The recruitment strategy was not elaborated for all participants stakeholder groups (e.g. no description for administrative staff). Suggestive of recruitment bias due to recruitment of older surgeons and lack views	No<br>Brief mention of themes reaching saturation. No detail on sample of administrative staff	No<br>No adequate detail about researcher's role, potential bias and influence during data collection, recruitment or relationship with participants was provided	Yes<br>The study had been approved by the relevant local institution. Partial information about obtaining informed consent (e.g. patients providing consent is clear. Information lacking about participants receiving sufficient explanation about study	Yes<br>Information on deriving themes from the data through the extraction of key words, key concepts resulting in preliminary categories was reported. The comparison and reporting of themes across stakeholder groups has been reported. The role of researcher, potential bias	No<br>The findings were presented clearly for the perceptions of patients and surgeons. However, perceptions of the administrative staff are unclear. The findings are not clearly discussed in relation to the third aim of the study, in particular to articulating clinic administrative	Major

				of younger surgeons			and data anonymity	and data selection for presentation is not presented	staff perceptions. Data analysis rigour discussed	
**Spaulding et al (2019)** 36]	Yes The aim of the study is clearly reported in abstract and introduction. Researchers provided rationale as to why the study was important to undertake	Yes The qualitative methodology is deemed appropriate, given the study sought to identify views of staff on implementation evaluation of electronic platform for PROMs administration	Yes Appropriate detail on study design and justification for the choice of interviews was discussed	Yes Participant selection was clearly explained. Discussion provided about any participants declining invitation	Yes Adequate detail provided explaining choice of method and the process of collecting data. Detail on conduct of interviews and data saturation discussed	No No adequate detail about researcher's role during data collection provided. No information on sample recruitment and choice of location discussed. Potential bias and influence during data collection was not discussed	Yes The study was approved by the local research ethics committees. Detail on obtaining informed consent, providing information to participants provided and data anonymity provided	Yes Appropriate detail on deriving themes using a framework approach was reported. The role of researcher, potential bias and data selection for presentation is provided	Yes The findings were presented clearly in relation to research aim. Data analysis rigour discussed and thorough	Minor
**Delgado-Herrera et al (2017)** [37]	Yes The aim of the study is clearly reported in abstract and introduction. Researchers provided rationale as to why the study was important to undertake	Yes The qualitative approach was deemed appropriate to explore participant views on testing of mobile application to capture PROs electronically and embed iterative feedback through three waves of interviews	Yes Appropriate detail on study design and justification for the choice of the interviews was discussed	No Participant selection was clearly explained. Adequate detail provided on appropriateness of study sample. No detail was provided about any participants declining invitation	Yes Detail provided on choice of method and the process of collecting data. Detail on conduct of mock interviews to identify issues with interview guide discussed. Saturation of data was not discussed	No No adequate detail about researcher’s examination of own role, potential bias and influence during sample recruitment and data collection provided. No detail provided on relationship with participants	Yes Study approved by Independent Review Board. All study subjects provided written informed consent for study participation	Yes Information on organising and categorisation of participant interpretation was reported. The role of researcher, potential bias and data selection for presentation was not presented	Yes The findings were presented clearly in relation to the research aim. Data analysis rigour not discussed	Minor
**Mou et al (2021)** [38]	Yes The aim of the study is clearly reported in the abstract and introduction of the paper. Researchers	Yes The qualitative approach was appropriate for exploring healthcare professional views on the barriers and	Yes Appropriate detail on study design and justification for the choice of the	No No adequate explanation about how participants were invited and about participants	Yes Detail provided on choice of method and process of collecting data. Detail on development of	No No adequate information provided on researcher’s examination of their own role, potential bias	Yes The study was approved by organisation’s Institutional Review Board. Detail on obtaining	Yes Appropriate detail on deriving themes reported. Partial description of	Yes The findings were presented clearly in relation to the research aim. Study limitations	Minor

	provided rationale as to why the study was important to undertake	facilitators of electronically capturing PROs	interviews discussed	declining invitation	interview guide and conducting interviews described. Discussed achieving data saturation	and influence during sample recruitment and data collection, and relationship with participants	informed consent provided	the role of researcher, potential bias and data selection for presentation provided	have been considered	
**Krawczyk et al (2019)** [39]	Yes The aim of the study is clearly reported in the abstract and introduction of the paper. Researchers provided rationale as to why the study was important to undertake	Yes The qualitative approach was appropriate for exploring healthcare professional views of using electronically captured patient-reported measures	Yes Appropriate detail on study design and justification for the choice of the focus groups and interviews discussed	No No adequate explanation about how participants were invited and about participants declining invitation	Yes Detail provided on choice of method and process of collecting data. Saturation of data was not discussed	No No adequate information provided on researcher's examination of their own role, potential bias and influence during sample recruitment and data collection, and relationship with participants	Yes The study was approved by relevant local ethics bodies. Detail on obtaining informed consent provided	Yes Appropriate detail on deriving themes reported. Partial description of the role of researcher, potential bias and data selection for presentation provided	Yes The findings were presented clearly in relation to the research aim. Data analysis rigour discussed and thorough. Study limitations have been considered	Minor
**Navarro-Millán et al (2019)** [40]	Yes The aim of the study is clearly reported in the abstract and introduction of the paper. Researchers provided rationale as to why the study was important to undertake	Yes The qualitative approach was appropriate to explore patients views on barriers and facilitators to reporting data electronically to monitor disease activity and to assess patients' willingness to share data with others	Yes Appropriate detail on study design and justification for the choice of the focus groups discussed	Yes Adequate detail provided on participant selection, appropriateness of study sample and invitation procedures. No detail on participants declining invitation	Yes Detail provided on choice of method and process of collecting data. Discussed use of theory to inform interview guide development and data saturation	No No adequate information provided on researcher's examination of their own role, potential bias and influence during sample recruitment and data collection, and relationship with participants	Yes The study was approved by organisation's Institutional Review Board. Detail on obtaining informed consent provided	Yes Appropriate detail on deriving themes reported. Partial description of the role of researcher, potential bias and data selection for presentation provided	Yes The findings were presented clearly in relation to the research aim. Data analysis rigour discussed and thorough. Study limitations have been considered	Minor
**Schick-Makaroff and Molzahn, (2017)** [41]	Yes The aim of the study is clearly reported in the abstract and introduction of the paper. Researchers	Yes The qualitative approach was appropriate to explore healthcare professional views on real-time use of	Yes Appropriate detail on study design and justification for the choice of the	Yes Adequate detail provided on participant selection, appropriateness of study sample and invitation	Yes Detail provided on choice of method and process of collecting data. Discussed data saturation	Yes The researcher's role during data collection, sample recruitment	Yes The study was approved by local ethics bodies. Detail on obtaining informed	Yes Appropriate detail on deriving themes reported. Description of the role of	Yes The findings were presented clearly in relation to the research aim. Data analysis rigour	None

	provided rationale as to why the study was important to undertake	PROMs collected electronically	interviews discussed	procedures. No detail on participants declining invitation		and choice of location was discussed	consent provided	researcher, potential bias and data selection for presentation provided	discussed and thorough. Study limitations have been considered	
**Brochmann et al (2016)** [42]	Yes The aim of the study is clearly reported in the abstract and introduction of the paper. Researchers provided rationale as to why the study was important to undertake	Yes The qualitative approach was appropriate to explore patient views of willingness to electronically complete PROMs	No No adequate detail on study design and justification for the choice of the focus groups and interviews discussed	Yes Adequate detail provided on participant selection, appropriateness of study sample and invitation procedures. No detail on participants declining invitation (qualitative study)	Yes Detail provided on choice of method and process of collecting data. Detail provided on the use of interview topic guide. Data saturation was not discussed	No No adequate information provided on researcher's examination of their own role, potential bias and influence during sample recruitment and data collection, and relationship with participants	Yes The study was approved by local ethics bodies. No adequate detail on obtaining informed consent (unclear whether informed consent from quantitative study applied to qualitative study)	No In-depth description of the analysis process was lacking. Unclear about the considerations of credibility, transferability and reliability in study. The researcher's role, potential bias and influence in data analysis were not reported	Yes The findings were presented clearly in relation to the research aim. Data analysis rigour not discussed. Study limitations have not been discussed in-depth	Major
**Snyder et al (2013)** [43]	Yes The aim of the study is clearly reported in the abstract and introduction of the paper. Researchers provided rationale as to why the study was important to undertake	Yes The qualitative approach was appropriate to explore patient and healthcare professional views from usability testing of a web system for PROMs collection	Yes Appropriate detail on study design and justification for the choice of the interviews discussed	Yes Adequate detail provided on participant selection, appropriateness of study sample and invitation procedures. Detail on participants declining invitation was not provided (qualitative study)	Yes Detail provided on choice of method and process of collecting data. Detail provided on the use of interview topic guide. Data saturation was not discussed	No No adequate information provided on researcher's examination of their own role, potential bias and influence during sample recruitment and data collection, and relationship with participants	Yes The study was approved by local ethics bodies. Detail on obtaining informed consent provided	No In-depth description of the analysis process was lacking. Unclear about the considerations of credibility, transferability and reliability in study. The researcher's role, potential bias and influence in data analysis were not reported	No The findings lacked in-depth clarity to address the research aim. Data analysis rigour not discussed. Study limitations have not been discussed in-depth	Major
**Tolstrup et al (2020)** [44]	Yes	Yes	Yes	Yes	Yes	Yes	Yes	Yes	Yes	None

	The aim of the study is clearly reported in the abstract and introduction of the paper. Researchers provided rationale as to why the study was important to undertake	The qualitative approach was appropriate, given the aim to understand patient and healthcare professional views of electronically reporting PROMs during cancer immunotherapy	Appropriate detail on study design and justification for the choice of the focus groups and interviews discussed	Adequate detail provided on participant selection, appropriateness of study sample and invitation procedures. Detail on participants declining invitation was provided	Detail provided on choice of method and process of collecting data. Detail provided on the use of interview topic guide. Data saturation was discussed	The researcher's role during data collection, sample recruitment and choice of location was discussed	The study was approved by local ethics bodies. Detail on obtaining informed consent and providing information to participants provided	Appropriate detail on deriving themes reported. Description of the role of researcher, potential bias and data selection for presentation provided	The findings were presented clearly in relation to the research aim. Data analysis rigour discussed and thorough. Study limitations have been considered	
**Yamada et al (2020)** [45]	Yes<br>The aim of the study is clearly reported in the abstract and introduction of the paper. Researchers provided rationale as to why the study was important to undertake	Yes<br>The qualitative approach was appropriate, given the aim to understand patient and healthcare professional views of electronically reporting PROMs during cancer immunotherapy	Yes<br>Appropriate detail on study design and justification for the choice of the interviews discussed	Yes<br>Adequate detail provided on participant selection, appropriateness of study sample and invitation procedures. Detail on participants declining invitation was not provided	Yes<br>Detail provided on choice of method and process of collecting data. Detail provided on the use of interview topic guide. Data saturation was discussed	Yes<br>The researcher's role during data collection, sample recruitment and choice of location was discussed	Yes<br>The study was approved by local ethics bodies. Detail on obtaining informed consent and providing information to participants provided	Yes<br>Appropriate detail on deriving themes reported. Description of the role of researcher, potential bias and data selection for presentation provided	Yes<br>The findings were presented clearly in relation to the research aim. Data analysis rigour discussed and thorough. Study limitations have been considered	None
**Baeksted et al (2017)** [46]	Yes<br>The aim of the study is clearly reported in the abstract and introduction of the paper. Researchers provided rationale as to why the study was important to undertake	Yes<br>The qualitative approach was appropriate, given the aim to understand patient and healthcare professional views of feasibility and acceptability of electronic collection of PROMs	Yes<br>Appropriate detail on study design and justification for the choice of the interviews discussed	Yes<br>Adequate detail provided on participant selection, appropriateness of study sample and invitation procedures. Detail on participants declining invitation was provided	Yes<br>Detail provided on choice of method and process of collecting data. Detail provided on the use of interview topic guide. Data saturation was discussed	Yes<br>The researcher's role during data collection, sample recruitment and choice of location was discussed	Yes<br>The study was approved by local ethics bodies. Detail on obtaining informed consent and providing information to participants provided	No<br>In-depth description of the analysis process was lacking. Unclear about the considerations of credibility, transferability and reliability in study. The researcher's role, potential bias and influence in data analysis were not reported	Yes<br>The findings were presented clearly in relation to the research aim. Data analysis rigour not discussed in-depth. Study limitations have been considered	Minor
**Samuel et al (2020)**	Yes	Yes	Yes	No	Yes	No	Yes	Yes	Yes	Minor

[47]	The aim of the study is clearly reported in the abstract and introduction of the paper. Researchers provided rationale as to why the study was important to undertake	The qualitative approach was appropriate, given the aim to understand patient views of electronic reporting of PROMs	Appropriate detail on study design and justification for the choice of the interviews discussed	No adequate explanation about how participants were invited and about participants declining invitation	Detail provided on choice of method and process of collecting data. Detail provided on the use of interview topic guide. Data saturation was not discussed	No adequate information provided on researcher's examination of their own role, potential bias and influence during sample recruitment and data collection, and relationship with participants	The study was approved by local ethics bodies. Detail on obtaining informed consent and providing information to participants provided	Appropriate detail on deriving themes reported. Description of the role of researcher, potential bias and data selection for presentation provided	The findings were presented clearly in relation to the research aim. Data analysis rigour not discussed in-depth. Study limitations have been considered	
**Nielsen et al (2021)** [48]	Yes<br>The aim of the study is clearly reported in the abstract and introduction of the paper. Researchers provided rationale as to why the study was important to undertake	Yes<br>The qualitative approach was appropriate, given the aim to understand patient views of electronic reporting of PROMs	Yes<br>Appropriate detail on study design and justification for the choice of the interviews discussed	Yes<br>Adequate detail provided on participant selection, appropriateness of study sample and invitation procedures. Detail on participants declining invitation was not provided	Yes<br>Detail provided on choice of method and process of collecting data. Data saturation was not discussed	Yes<br>The researcher's role during data collection, sample recruitment and choice of location was discussed	Yes<br>The study followed local ethics committee guidelines. Detail on obtaining informed consent and providing information to participants provided	Yes<br>Appropriate detail on deriving themes reported. Description of the role of researcher, potential bias and data selection for presentation provided	Yes<br>The findings were presented clearly in relation to the research aim. Data analysis rigour discussed partially. Study limitations have been considered	None
**Lehmann et al (2021)** [49]	Yes<br>The aim of the study is clearly reported in the abstract and introduction of the paper. Researchers provided rationale as to why the study was important to undertake	Yes<br>The qualitative approach was appropriate, given the aim to understand patient views of using a web-based patient portal to report PROMs electronically	Yes<br>Appropriate detail on study design and justification for the choice of the interviews discussed	Yes<br>Adequate detail provided on participant selection, appropriateness of study sample and invitation procedures. Detail on participants declining invitation was provided	Yes<br>Detail provided on choice of method and process of collecting data. Data saturation was not discussed	Yes<br>The researcher's role during data collection, sample recruitment and choice of location was discussed	Yes<br>The study was approved by local ethics bodies	No<br>In-depth description of the analysis process was lacking. Unclear about the considerations of credibility, transferability and reliability in study. The researcher's role, potential bias and influence in data analysis	No<br>No clear reporting of findings from the 3 waves of interviews in relation to research aim. No explanation about the credibility of the findings provided, such as the inclusion of two or multiple researchers in the synthesis of findings.	Minor

								were not reported	Limited supporting quotes. Study limitations have been considered	
**Duman-Lubberding et al (2017)** [50]	Yes The aim of the study is clearly reported in the abstract and introduction of the paper. Researchers provided rationale as to why the study was important to undertake	Yes The qualitative approach was appropriate, given the aim to understand healthcare professional views of use of electronic platform to review PROMs	No No adequate detail on study design and justification for the choice of the interviews discussed (for study aim D)	No No adequate explanation about how participants were invited and about participants declining invitation	No Setting clearly described. Minimal explanation provided on how interviews were conducted. No detail on the use of interview topic guide and data saturation was not discussed	No No adequate information provided on researcher's examination of their own role, potential bias and influence during sample recruitment and data collection, and relationship with participants	Yes Ethical approval deemed unnecessary as this had been considered part of regular patient care	No In-depth description of the analysis process was lacking. Unclear about the considerations of credibility, transferability and reliability in study. The researcher's role, potential bias and influence in data analysis were not reported	Yes The findings were presented clearly in relation to the research aim. Data analysis rigour not discussed	Major
**Grossman et al (2018)** [51]	Yes The aim of the study is clearly reported in the abstract and introduction of the paper. Researchers provided rationale as to why the study was important to undertake	Yes The qualitative approach was appropriate, given the aim to understand patient and healthcare professional views of value, challenges and usability of electronic reporting/collecting of PROMs	Yes Appropriate detail on study design and justification for the choice of the interviews discussed	Yes Adequate detail provided on participant selection, appropriateness of study sample and invitation procedures. Detail on participants declining invitation was not provided	Yes Detail provided on choice of method and process of collecting data. Detail on using an interview guide and data saturation discussed	Yes The researcher's role during data collection, sample recruitment and choice of location was discussed	Yes The study followed local ethics committee guidelines. Detail on obtaining informed consent and providing information to participants provided	Yes Appropriate detail on deriving themes reported. Description of the role of researcher, potential bias and data selection for presentation partially provided	Yes The findings were presented clearly in relation to the research aim. Data analysis rigour discussed. Study limitation have been considered	None
**Moradian et al (2018)** [52]	Yes The aim of the study is clearly reported in the abstract and introduction of the paper.	Yes The qualitative approach was appropriate, given the aim to understand patient views of the	Yes Appropriate detail on study design and justification for the choice of the	Yes Adequate detail provided on participant selection, appropriateness of study	Yes Detail provided on choice of method and process of collecting data. Detail on data	No No adequate information provided on researcher's examination of their own role,	Yes The study was approved by local ethics bodies	Yes Appropriate detail on deriving themes reported. Description of	Yes The findings were presented clearly in relation to the research aim. Data analysis	Minor

	Researchers provided rationale as to why the study was important to undertake	usability of mobile phone-based electronic reporting of PROMs	interviews discussed	sample. Partial information about invitation procedures. Detail on participants declining invitation was not provided	saturation discussed	potential bias and influence during sample recruitment and data collection, and relationship with participants		the role of researcher, potential bias and data selection for presentation not provided	rigour briefly discussed. Study limitation have not been discussed in-depth	
**Sandhu et al (2020)** [53]	Yes The aim of the study is clearly reported in the abstract and introduction of the paper. Researchers provided rationale as to why the study was important to undertake	Yes The qualitative approach was appropriate, given the aim to understand healthcare professional views of integrating electronically collected PROMs in routine care	Yes Appropriate detail on study design and justification for the choice of the interviews discussed	Yes Adequate detail provided on participant selection, appropriateness of study sample. Detail on participants declining invitation was not provided	Yes Detail provided on choice of method and process of collecting data. Detail on development of an interview guide and data saturation discussed	Yes The researcher's role during data collection, sample recruitment and choice of location was partially discussed	Yes The study followed local ethics committee guidelines. Detail on obtaining informed consent and providing information to participants provided	Yes Appropriate detail on deriving themes reported. Description of the role of researcher, potential bias and data selection for presentation were partially discussed	Yes The findings were presented clearly in relation to the research aim. Data analysis rigour discussed. Study limitation have been discussed	None

*'None': all ‘yes’ answers on CASP assessment; ‘minor’: ≤2 ‘no’ answers on CASP assessment; ‘major’: >2 ‘no’ answers on CASP assessment.
