# Supplementary material for: Barriers and facilitators to the uptake of electronic collection and use of patient-reported measures in routine care of older adults: a systematic review with qualitative evidence synthesis
Source: JAMIA Open. 2024 Aug 2;7(3):ooae068. doi: 10.1093/jamiaopen/ooae068 (PMC11296862; doi:10.1093/jamiaopen/ooae068)
Supplement: ooae068_Supplementary_Data [file ooae068_supplementary_data.zip › ooae068_Supplementary_Data/Appendix 5 - Findings, NASSS and COM-B defined_refs.docx]

**Supplemental appendix 5:**

- Findings describing stakeholder perspectives of barriers and facilitators – Pages 1 to 5
- Definitions of theoretical domains – Pages 6 to 7
- Mapping of barriers and facilitators to NASSS and TDF domains – Pages 7 to 9

**Findings describing stakeholder perspectives of barriers and facilitators to the electronic collection and use of PRMs in older adults’ care**

| **Findings describing stakeholder perspectives of barriers and facilitators to implementation of the electronic collection and use of PRMs in older adults’ care** | | | |
| --- | --- | --- | --- |
| **#** | **Description of finding** | **Studies contributing to finding** | **Confidence in the evidence** |
| **Thematic category: Older adults’ characteristics** | | |  |
| ***Clinical conditions and socio-cultural factors*** | | | |
| 1 | Older adults with physical disability, visual and cognitive impairment, and communication difficulties (e.g., PRMs administered in non-native language) reported that electronic completion of PRMs could be challenging.  Caregivers perceived that older adults experienced problems in concentrating for a long period of time when completing PRMs.  Healthcare professionals cited that older adults completing PRMs in a non-native language and those with cognitive disabilities experienced difficulty in comprehending questions, while visually impaired older adults found switching between multiple screens on an electronic device difficult.  Administrative staff also cited that older adults completing PRMs administered in a non-native language were less likely to understand questions (*) | [10, 33, 34, 36, 38, 40, 42, 46, 48, 51, 52] | **Moderate confidence** |
| ***Access to digital technology*** | | | |
| 2 | As cited by some older adults and healthcare professionals, the lack of access to necessary digital technology (e.g., electronic devices and e-mail) hindered older adults’ capability to electronically complete PRMs. Similarly, older adults and healthcare professionals cited that having access to necessary digital technology (e.g., electronic devices and internet) facilitated older adults’ completion of PRMs electronically | [10, 35, 40, 50, 53] | **Moderate confidence** |
| **Thematic category: Digital Technology** | | |  |
| ***User interface for older adults*** | | | |
| 3 | Older adults reported that having an easy-to-use, intuitive interface with the following features facilitated electronic completion of PRMs:   - large font and screen size - visually appealing with colour, images and graphs - clear labelling of response options and data - indication of questionnaire progress (e.g., progress bar) - ability to download or bookmark link to PROMs software on various electronic devices - multiple questions per page   Healthcare professionals believed that an easy-to-use and visually appealing interface enables older adults to complete PRMs electronically.  Older adults not having to view all the questions at once when completing PRMs electronically was cited as a facilitator by healthcare professionals and administrative staff.  Some older adults and healthcare professionals believed that a touchscreen option facilitated older adults’ completion of PRMs electronically | [10, 33, 35, 36, 37, 43, 44, 45, 47, 49, 52] | **Moderate confidence** |
| 4 | Older adults reported that the following interface features hindered their ability to complete PRMs electronically:   - small font and screen size - many scrolls to navigate through questionnaire - use of certain display graphics (e.g., use of multiple colours, checkered boxes, circle options and smiley faces) - unresponsive option and progress buttons - unlabelled response options - unnecessary pop-up of alerts - mobile application downloading features - drop downs were cumbersome   Some healthcare professionals cited that it was stressful for visually impaired older adults to click through multiple screens to complete PRMs electronically.  Caregivers and older adults experienced frustration when they could not progress through the questionnaire. Some healthcare professionals also cited that older adults experienced frustration with challenges in questionnaire progression.  Some older adults had difficulty using a touchscreen in absence of a touchscreen pen, due to the need to firmly press or for long periods with their fingers | [10, 33, 35, 36, 37, 38, 44, 45, 47, 52] | **Moderate confidence** |
| ***User interface for healthcare professionals*** | | | |
| 5 | Some healthcare professionals valued an easy-to-use interface that helped them access questionnaires on various devices and browsers, with a visually appealing layout that used colour to draw attention to areas of concern and/or changes over time, to engage in the electronic collection and use of PRMs. Some perceived that difficulties in accessing the electronic system, lack of clear labelling of questions, the need for many clicks to access key information and limited visual presentation of data were barriers to electronic collection and use of PRMs | [34, 35, 36, 38, 41, 43, 46, 53] | **High confidence** |
| ***Electronic device for PRMs completion*** | | | |
| 6 | Older adults generally were comfortable completing PRMs on portable electronic devices such as a laptop, tablet or smartphone, or on a desktop computer. Some older adults preferred using smartphones due to its screen colours and convenience to use the device, while some preferred a computer or tablet due to the larger screen size and reduced need for scrolling. A few older adults experienced difficulty in using a smartphone due to the side-to-side scrolls required | [10, 33, 35, 44, 45, 46, 48, 52] | **Moderate confidence** |
| ***Technical challenges*** | | | |
| 7 | Most older adults and healthcare professionals reported that technical challenges such as connectivity problems, device outages (e.g., going dark, logging out or system crashes), slow response time and device incompatibilities were barriers to electronic completion and use of PRMs. | [10, 34, 35, 36, 37, 38, 46, 47] | **Moderate confidence** |
| ***Privacy and security of personal data*** | | | |
| 8 | Not knowing who could access personal and health data and what the data is used for, and exposure of data to malware viruses and hackers were cited as barriers to electronic completion of PRMs by most older adults. However, some older adults had no concerns about privacy risks when using their own device.  Healthcare professionals were concerned about data privacy and governance (e.g., who owns the data) when using certain software programs for patient data collection | [35, 45] | **Low confidence** |
| **Thematic category: Support from social circle** | | |  |
| 9 | Older adults cited that support from family, caregivers, peers, healthcare professionals and healthcare services received in the form of help to understand and complete PRMs, support to use digital technology to complete PRMs, encouragement and reminders facilitated electronic completion of PRMs.  Healthcare professionals believed support from family and peers, while administrative staff believed helping patients (e.g., translation services for patients with language barriers) during completion of PRMs facilitated older adults’ completion of PRMs electronically.  One caregiver reported helping the older adult complete PROMs due to the cognitive challenges experienced by the older adult. (*) | [10, 35, 36, 40, 41, 42, 44, 45, 47, 49] | **Moderate confidence** |
| **Thematic category: Knowledge and skills** | | |  |
| ***Digital knowledge and skills*** | | | |
| 10 | Some older adults cited that having adequate knowledge and skills in using digital technology facilitated completion of PRMs electronically.  As cited by most older adults, administrative staff and healthcare professionals, older adults lacking adequate digital technology knowledge and skills made older adults feel uncomfortable and less confident when completing PRMs electronically. | [10, 36, 40, 45, 46, 48, 51, 52, 53] | **Moderate confidence** |
| ***Health knowledge and literacy in general*** | | | |
| 11 | Older adults and healthcare professionals cited that older adults lacking adequate health knowledge found it difficult to understand medical terminology and health information in questionnaires, that made it challenging to electronically complete PRMs. Healthcare professionals cited that some of these older adults found difficulty in discerning between different health conditions they experienced.  Caregivers experienced difficulty in understanding medical terminology, which acted as a barrier to electronic completion of PRMs on behalf of older adults.  Administrative staff cited that low literacy levels in older adults limited their reading, hindering electronic completion of PRMs. (*) | [10, 36, 46, 48, 51, 53] | **Moderate confidence** |
| ***PRMs interpretation knowledge and skills*** | | | |
| 12 | A few healthcare professionals reported that receiving a guideline for interpretation of PRMs responses facilitated electronic collection and use of PRMs. Most healthcare professionals reported the lack of knowledge and skills to interpret PRMs responses and address problem areas hindered electronic collection and use of PRMs. (*) | [35, 38, 41] | **Low confidence** |
| ***Regular exposure to PRMs enhancing health knowledge*** | | | |
| 13 | Older adults and healthcare professionals perceived that completing PRMs electronically helped improve older adults’ digital knowledge and skills, and health knowledge, and increased awareness of their own health condition in the long term. | [41, 44, 46, 47, 48, 51] | **High confidence** |
| ***Rationale for PRMs collection and use*** | | | |
| 14 | Older adults and healthcare professionals reported that older adults feeling obliged to complete PRMs for healthcare professionals without understanding the rationale for PRMs collection was a barrier to electronic completion of PRMs. (*) | [35, 38, 45, 50] | **Low confidence** |
| **Thematic category: Motivation and incentives for capture and use of PRMs** | | |  |
| ***Older adults’ motivation and incentives*** | | | |
| 15 | Older adults reported the following factors as motivators for electronic completion of PRMs:   - owning responsibility to improve one’s own health and awareness of their health (*) - providing adequate information about their health to healthcare professionals for care planning (*) - helping advance health research, and help other patients improve health outcomes through research advancements (*) - healthcare professionals reviewing, discussing and using PRMs responses during consultations to improve care (*) - trust in healthcare provider (e.g., hospital asked to use digital PRM system) - self-monitoring of condition based on PRMs responses (*) | [35, 40, 43, 44, 45, 46, 47, 48, 51] | **Moderate confidence** |
| 16 | As cited by some older adults and healthcare professionals, older adults were demotivated to complete PRMs electronically if they believed it did not influence their health and the care they received, and when their healthcare professional did not review, discuss and use PRMs responses during their consultations.  Some older adults reported that a negative reaction to the PRMs responses by their healthcare professional discouraged them from completing PRMs electronically. (*) | [35, 40, 43, 44, 45, 47, 48, 49, 50, 51] | **Moderate confidence** |
| ***Healthcare professionals’ motivation and incentives*** | | | |
| 17 | Healthcare professionals reported the following factors as motivators to electronic collection and use of PRMs:   - involvement of patients to voice their perception of health through PRMs completion (*) - PRMs response data completeness (*) - PRMs responses informing quality improvements and research advancements (*) - enhanced documentation based on PRMs responses - collaboration with multi-disciplinary care teams to improve care based on PRMs responses (*) - regulatory directives (e.g., PRMs data as part of data registries) mandating PRMs data collection (*) - shared decision making and tailored care as a result of PRMs use (*) - immediate access to PRMs responses and meaningful presentation of PRMs responses for clinical decision-making | [34, 35, 36, 38, 39, 41, 43, 46, 50, 53] | **Moderate confidence** |
| 18 | Healthcare professionals reported that the following factors demotivated them to electronically collect and use PRMs:   - believing that PRMs did not influence the care they provided (*) - discrepancies in health assessment between the patient and themselves (*) - PRMs responses providing insufficient information (*) - organisational or regulatory directive to collect and use PRMs, in absence of clear messaging of rationale and benefits for collection and use of PRMs (*) - believing PRMs collection and use disrupts patient care duties (*) - non-completion of PRMs questions and low response rates (*) - non-discipline specific PRMs collection and use (*) - healthcare professional characteristics associated with older age, higher length of service and not reviewing PRMs regularly (*) - lack of immediate access to and meaningful presentation of PRMs responses for clinical decision-making | [34, 35, 36, 38, 43, 44, 46, 50, 53] | **Moderate confidence** |
| ***Acceptance of intervention by older adults*** | | |  |
| 19 | Administrative staff cited that acceptance of electronic completion of PRMs by older adults motivated them to support the process of collecting PRMs electronically | [36] | **Low confidence** |
| ***Location of PRMs administration*** | | |  |
| 20 | Older adults found completing PRMs questions and reviewing responses from home or remote location (outside the clinic) was convenient and facilitated electronic completion of PRMs.  The need to spend extra time in the clinic to complete PRMs, incurring transport and parking costs to be physically present in the clinic, hygiene and data privacy risks associated with using shared devices and feeling rushed were barriers to completing PRMs electronically in the clinic, as reported by most older adults.  Incomplete questionnaires due to limited time for older adults to complete PRMs in the clinic was reported as a barrier to electronic collection and use of PRMs by some healthcare professionals. Some healthcare professionals perceived having an in-clinic option to complete PRMs may facilitate older adults’ PRMs completion rates | [10, 33, 35, 38, 42, 43, 45, 47, 48, 51, 53] | **Moderate confidence** |
| ***Timing and frequency of PRMs collection*** | | |  |
| 21 | Some older adults reported that completing PRMs at a regular frequency (e.g., once a week or once a month) and close to the day of consultation facilitated electronic completion of PRMs. Infrequent completion of PRMs that led to no changes in health, and that reduced familiarity with the electronic system for PRMs completion were cited as barriers to electronic completion of PRMs by some older adults.  Healthcare professionals cited that the lack of PRMs completion closer to the day of consultation was a barrier to electronic collection and use of PRMs. (*) | [42, 44, 45, 49, 50, 53] | **Moderate confidence** |
| ***Access to additional resources based on PRMs responses*** | | |  |
| 22 | Most older adults and healthcare professionals reported that access to additional medical or non-medical resources (e.g., self-management guidance or social support) based on PRMs responses encouraged older adults to electronically complete PRMs.  Some healthcare professionals reporting lack of resources to offer (e.g., education, social services), and some older adults reporting inability to access resources (e.g., getting questions answered from healthcare professional or personalised information) based on PRMs responses were barriers to electronic collection and use of PRMs. (*) | [35, 38, 40, 44, 47, 48, 49, 50, 51, 52] | **Moderate confidence** |
| **Thematic category: Emotional experience** | | | |
| 23 | Some older adults reported that answering questions about their health conditions made them emotionally elevated, encouraging them to complete PRMs electronically. Some healthcare professionals believed that older adults may feel less embarrassed to electronically complete PRMs especially related to personal and sensitive topics (e.g., related to mental health, sex life and social determinants) rather than talk about them initially, which may facilitate older adults’ completion of PRMs electronically.  Some older adults and healthcare professionals cited that emotional distress caused by having to respond to questions on health conditions, and personal and sensitive topics were barriers to older adults’ completion of PRMs electronically. Anxiety caused by the limited time available to complete PRMs in the clinic was reported as a barrier to electronically completing PRMs by some older adults. (*) | [10, 33, 38, 41, 43, 44, 48, 50, 51] | **Moderate confidence** |
| **Thematic category: Older adults’ autonomy** | | | |
| 24 | As cited by some older adults and healthcare professionals, the influence from family, caregivers, administrative staff or healthcare professionals sometimes led to the suppression of the older adults’ voice, discouraging older adults to complete PRMs electronically. (*) | [33, 36, 39, 427, 45] | **Moderate confidence** |
| **Thematic category: Patient – healthcare professional communication** | | |  |
| 25 | Structured communication during consultations (e.g., discussion of results focusing on problem areas and changes over time), enhanced patient – healthcare professional interactions and patient involvement in voicing their perception of health were reported as facilitators to electronic collection and use of PRMs by some older adults and healthcare professionals.  Some older adults and healthcare professionals reported that PRMs completion made the patient – healthcare professional interactions impersonal, discouraging the electronic completion of PRMs. Some older adults feared that relying on electronic systems to communicate about health may lead to the replacement of face-to-face consultations, and lack of support from healthcare professionals and the healthcare service. (*) | [34, 35, 36, 38, 39, 40, 41, 43, 44, 45, 46, 47, 48, 50, 51] | **Moderate confidence** |
| **Thematic category: Workflow** | | | |
| ***Efficiencies, time constraints and changes to work routines*** | | |  |
| 26 | Facilitators to electronic collection and use of PRMs reported by some healthcare professionals were improved clinic efficiency through the use of electronic systems to screen PRMs responses and determine patient needs prior to the consultation, reduced clerical time due to patients independently completing PRMs electronically, enhanced EHR documentation based on PRMs responses and automatic distribution of questionnaire, and time saved through focussed patient assessments based on PRMs responses.  Some older adults and administrative staff reported that knowing healthcare professionals save time using the electronic system to access and use PRMs encouraged engagement with electronic completion of PRMs.  Barriers to electronic collection and use of PRMs cited by many healthcare professionals were time constraints and new routines associated with incorporation of PRMs in care and use of electronic systems to access PRMs responses, inundation of PRMs response data resulting in having too many things to discuss with the patient, delays to clinic workflows associated with challenges in PRMs administration (e.g., questionnaires not working or patients not arriving early to complete PRMs in time before the appointment), challenges with seamless data access and additional work created by having to cooperate with other multi-disciplinary healthcare professionals. (*) | [34, 35, 36, 38, 39, 41, 43, 44, 45, 46, 51, 53] | **Moderate confidence** |
| ***Additional work with PRMs completion*** | | |  |
| 27 | Some older adults and healthcare professionals cited that older adults perceived PRMs completion was additional work and use of electronic systems to complete PRMs was time consuming, hindering older adults from electronically completing PRMs. (*) | [43, 45, 47, 50] | **Low confidence** |
| **Thematic category: Organisational factors** | | |  |
| ***Team culture and collaboration*** | | |  |
| 28 | Multi-disciplinary team communication, positive team culture and peer to peer support were cited as facilitators to electronic collection and use of PRMs by some healthcare professionals, while some reported that challenges in multi-disciplinary collaboration and different levels of motivation within the team to collect and use PRMs were barriers electronic collection and use of PRMs. (*) | [34, 35, 36, 39, 41] | **Moderate confidence** |
| ***Leadership and champions*** | | |  |
| 29 | Healthcare professionals reported that supportive leadership and having champions facilitated electronic collection and use of PRMs. They valued the mentorship they received and buy-in from their leadership. (*) | [34, 36, 39] | **Moderate confidence** |
| ***Change management*** | | |  |
| 30 | Some healthcare professionals cited that the flexibility provided to rapidly iterate the electronic system for better acceptance and having a clearly communicated operational process facilitate the electronic collection and use of PRMs. Lack of uniformity in the implementation process and lack of established operational workflows (e.g., training and accessing PRMs electronically) were reported as barriers to electronic collection and use of PRMs by some healthcare professionals.  Older adults and administrative staff believed training and support workflows (e.g., trouble shoot technical problems) facilitated the electronic collection of PRMs. (*) | [34, 36, 38, 43, 44, 46, 47, 53] | **Moderate confidence** |
| ***Resources to manage collection and use of PRMs*** | | |  |
| 31 | Some older adults reported that receiving adequate technical support and education would facilitate completion of PRMs electronically. The lack of technical support and necessary education to help with completing PRMs electronically were cited as barriers by older adults. The technical support available to resolve any technical problems was cited as a facilitator to electronic completion of PRMs by some older adults.  Healthcare professionals reported that adequate education, support staff, support in the form of reminders during consultation hours prompting them to review PRMs responses, digital technology infrastructure that support seamless data integration, and government funding to cover digital technology infrastructure costs were facilitators to the electronic collection and use of PRMs. Barriers to electronic collection and use of PRMs cited by healthcare professionals were lack of digital technology infrastructure to support integration of PRMs in EHRs or other platforms clinicians use, education (e.g., to use electronic systems and interpret PRMs responses), high costs and lack of support staff. Healthcare professionals believed adequate education, digital technology infrastructure that allowed for integration of PRMs with EHRs, funding and support staff facilitated electronic collection and use of PRMs.  Administrative staff particularly felt that access to interpreter services, technical support staff and adequate education facilitated electronic collection of PRMs. (*) | [34, 35, 36, 38, 39, 40, 43, 44, 45, 46, 50, 52] | **Moderate confidence** |
| **Thematic category: PRMs Questionnaire selection and design** | | |  |
| ***Questionnaire length and complexity of questions*** | | |  |
| 32 | Some older adults cited that having simple, easy-to-understand questions facilitated electronic completion of PRMs. Some older adults and healthcare professionals cited that lengthy questionnaires containing hard to comprehend questions and those that took a long period of time to complete were barriers to electronic completion of PRMs.  Some healthcare professionals believed that questions should be asked in a way that would highlight the patient’s problems and that the overlapping number of questions asked repeatedly during each clinic visit may be reduced by sharing PRMs responses between clinicians. (*) | [33, 36, 38, 41, 42, 44, 45, 46, 47, 48, 51, 53] | **High confidence** |
| ***Questions relevant to patient’s health*** | | |  |
| 33 | Some older adults and healthcare professionals reported that having questions specific to the patient’s health (e.g., behavioural activation, mental health and sexual function) facilitated electronic collection and use of PRMs. Some older adults and healthcare professionals cited that the lack of specific questions addressing patient’s health (e.g., co-morbid conditions and specific disease) hindered electronic collection and use of PRMs. (*) | [36, 38, 43, 47, 48, 51, 53] | **Moderate confidence** |
| ***PRMs response capture option*** | | |  |
| 34 | Some older adults found a free-text field to add comments to qualify the numerical rating of their health facilitated electronic completion of PRMs. The lack of a free text field to capture patient’s health was cited as a barrier by some older adults and healthcare professionals. Some older adults reported they could not use the free text field due to difficulties in information articulation or if they experienced dyslexia, hindering electronic completion of PRMs.  Most healthcare professionals found the numerical rating of patient health as more useful, while some healthcare professionals found free text description of patient health as more useful. (*) | [38, 39, 41, 42, 43, 44, 48, 51] | **Moderate confidence** |

Abbreviations: PRM, Patient-reported Measures; EHR, Electronic Health Record

Findings that are not unique to electronic collection and use of PRMs, and overlap with findings from existing literature on PRMs implementation in general are marked with an asterisk (*)

**Definitions of theoretical domains for Capability, Opportunity, Motivation, Behaviour model (COM-B) expanded by Theoretical Domains Framework (TDF), and Non-adoption, Abandonment, challenges to the Scale-up, Spread, Sustainability (NASSS) framework**

**COM-B mapped to TDF, and their definitions**

| **COM-B component** | **TDF domain** |
| --- | --- |
| **Capability** |  |
| Psychological - Knowledge or psychological skills, strength or stamina to engage in necessary thought processes | **Knowledge** - An awareness of the existence of something |
|  | **Memory, attention and decision processes** - The ability to retain information, focus selectively on aspects of the environment and choose between two or more alternatives |
|  | **Behavioural regulation** - Anything aimed at managing or changing objectively observed or measured actions *(Ability to maintain one’s own behaviour)* |
| Physical - Physical skills to engage in necessary physical processes | **Skills** - An ability or proficiency acquired through practice |
| **Opportunity** |  |
| Social - Opportunity afforded by interpersonal influences, social cues and cultural norms that impact the way we think about things | **Social influences** - Those interpersonal processes that can cause an individual to change their thoughts, feelings or behaviours |
| Physical - Opportunity afforded by the environment (resources, locations, physical affordance) | **Environmental context and resources** - Any circumstance of a person’s situation or environment that discourages or encourages the development of skills and abilities, independence, social competence and adaptive behaviour |
| **Motivation** |  |
| Reflective - Reflective processes involving evaluations/beliefs about good/bad and plans (self-conscious intentions) | **Social/professional role and identity** - A coherent set of behaviours and displayed personal qualities of an individual in a social or work setting. |
|  | **Beliefs about capabilities** - Acceptance of the truth, reality or validity about an ability, talent or facility that a person can put to constructive use |
|  | **Optimism** - The confidence that things will happen for the best, or that desired goals will be attained. |
|  | **Intentions** - A conscious decision to perform a behaviour or a resolve to act in a certain way. |
|  | **Goals** - Mental representation of outcomes or end states that an individual wants to achieve. |
|  | **Belief about consequences** - Acceptance of the truth, reality or validity about outcomes of a behaviour in a given situation. |
| Automatic - Automatic processes involving emotions, impulses, desires, inhibitions arising from associative learning and/or innate dispositions | **Reinforcement** - Increasing the probability of a response by arranging a dependent relationship or contingency, between the response and a given stimulus. |
|  | **Emotion** – A complex reaction pattern, involving experiential, behavioural, and psychological elements, by which the individual attempts to deal with a personally significant matter or event |

**NASSS Domains and their definitions**

| **NASSS Domain** | **Construct(s)** |
| --- | --- |
| **Patient condition:**  clinical condition, comorbidities, sociocultural and socio-economic aspects that determine individual’s ability to use digital technology | Clinical conditions – Understanding conditions such as existing clinical conditions, physical disabilities and cognitive or visual impairment |
|  | Comorbidities – Existing complex care needs for multiple conditions |
|  | Socio-economic aspects – Access to digital technology, general literacy, and health and digital literacy |
|  | Socio-cultural aspects – Social influence, and cultural traditions and norms impacting |
| **Technology:**  digital technology equipment, features and functionality, knowledge generated knowledge and support needed, and supply model | Technology features - Material and technical features, and functionality of the digital technology |
|  | Knowledge generated from use of digital technology - Data accuracy and the extent to which data generated is accepted, trusted and considered sufficient for decision-making are included |
|  | Knowledge and support needed to use the technology – The extent to which knowledge and support is provided to use digital technology |
|  | Technology supply model – Addresses procurement of the digital technology, nature of the client-supplier relationship, and the level of potential substitutability via the marketplace. For example, the extent to which digital technology is interoperable, and whether it is an off the shelf or bespoke solution, and risks of supplier withdrawal from market |
| **Value proposition:**  whether or not the digital technology is worth developing and implementing for older adults, healthcare professionals, healthcare providers and suppliers | Upstream value – Addresses value to the supply-side, that apply to the sponsor of the digital technology (e.g., supplier, developer or healthcare system as the sponsor) |
|  | Downstream value – Addresses value to the demand-side, that apply to the patients, healthcare professional and/or payers |
| **Adopter system:**  changes in role, identity, and acceptance of digital technology by different stakeholders | **Adoption by staff –** Addresses acceptance, and changes in roles and practices with use of digital technology |
|  | **Adoption by patients -** Addresses acceptance, and changes in demands to use digital technology |
|  | **Impact of caregivers on adoption by patients –** Addresses availability and behaviour of the lay caregivers on acceptance and adoption of digital technology by patients |
| **The organisation:**  readiness, capacity, and work needed to implement digital technology in organisation | **Organisation’s innovation capacity –** The extent to which an organisation has the capacity (i.e., resources, leadership, professional relationships and absorptive capacity for new knowledge) to embrace service-level innovation |
|  | **Organisation’s readiness** – The extent to which an organisation is ready (i.e., tension for change and balance of supporters and opponents) to implement specific digital technology |
|  | **Adoption and funding decision** – The extent to which an organisation can make funding decisions (i.e., inter-organisational funding vs. system level funding), and has funding available to support digital technology implementation |
|  | **Disruption to existing work routines -** The extent to which implementation of digital technology will disrupt existing work routines (e.g., ensure staff buy-in and change management) |
|  | **Work involved in implementation –** The extent of work required to implement digital technology |
| **Wider context:**  policy context, regulatory or professional body influence, public perceptions and inter-organisational networking | The extent to which social/professional, political, regulatory, technological and economic context impacts implementation of digital technology. This domain also addresses any existing networks between organisations and how they impact implementation of digital technology |
| **Continuous embedding and adaptation over time:**  scope for adaptation over time and organisation resilience | **Adaptation feasibility of digital technology –** The scope to adapting and coevolving the digital technology and the service over time |
|  | **Organisational resilience –** The extent to which the organisation is resilient to handling critical events and adapting to unforeseen eventualities: The ability to sustain digital technology and adjust with adaptations that arise through the interactions of other domains, and in the presence of continued stress |
