# Supplementary material for: Barriers and facilitators to the uptake of electronic collection and use of patient-reported measures in routine care of older adults: a systematic review with qualitative evidence synthesis
Source: JAMIA Open. 2024 Aug 2;7(3):ooae068. doi: 10.1093/jamiaopen/ooae068 (PMC11296862; doi:10.1093/jamiaopen/ooae068)
Supplement: ooae068_Supplementary_Data [file ooae068_supplementary_data.zip › ooae068_Supplementary_Data/Appendix 6 - Extracted data relevant to findings.docx]

**Supplemental appendix 6 – Extracted data relevant to findings (Supportive quotes)**

**Notes:** Black text – author’s interpretation; *Blue text* – participant quotes

**Thematic category: OLDER ADULTS’ CHARACTERISTICS**

**Finding #1:** Older adults with physical disability, visual and cognitive impairment, and communication difficulties (e.g., PRMs administered in non-native language) reported that electronic completion of PRMs could be challenging.

Caregivers perceived that older adults experienced problems in concentrating for a long period of time when completing PRMs.

Healthcare professionals cited that older adults completing PRMs in a non-native language and those with cognitive disabilities experienced difficulty in comprehending questions, while visually impaired older adults found switching between multiple screens on an electronic device difficult.

Administrative staff also cited that older adults completing PRMs administered in a non-native language were less likely to understand questions

| **#** | **Reference** | **Perspective of:** | **Extracted data relevant to finding** |
| --- | --- | --- | --- |
| **BARRIERS** | | | |
| 1 | Aiyegbusi et al (2018) | Patients | Patients had varying levels of dexterity issues controlling the mouse. Two of them were able to scroll up and down the pages without assistance but with some difficulty while the third (rare user) had more difficulty controlling the cursor and needed the interviewer to locate the cursor on two occasions in order to continue with the tasks.  *Nothing complicated … it’s controlling the mouse* |
| 2 | Amini et al (2021) | HCP | Language barriers experienced by patients with a primary language other than Dutch |
| 3 | Spaulding et al (2019) | HCP | HCPs discussed patients' disease characteristics, cognitive, intellectual, and visual impairment also influenced patients' interest in using the ePSRM tool  *So, people that [have] developmental disabilities of course … they are not fully comprehending what the questions are trying to ask … so patients with dementia, patients with developmental disabilities …* |
| 4 | Mou et al (2021) | HCP | *Need to have this in other languages - this is discriminatory to our immigrant/English as their second language (ESL) patients*  *We need Arabic, Portuguese, Vietnamese, French Creole etc. to match the population we see* |
| 5 | Navarro-Millán et al (2019) | Patients | Many participants expressed their inability to type on a computer keyboard or phone keypad during symptoms of fatigue or hand pain |
| 6 | Long et al (2021) | Administrative staff | Staff highlighted that non-English speaking patients frequently struggle with completing questionnaires in English  *let’s not forget [the patients] who don’t speak English. If you can’t speak it, you’re sure not reading it.* |
| 7 | Long et al (2021) | Patients | Population included patients with UE conditions of varying etiologies causing varying degrees of disability. This introduced unique physical challenges to completing PROMs.  Another patient with chronic UE disability noted that the tablet in his home is mounted on a stand because he cannot hold the device  Poor vision was a barrier to PROM completion  *Vision troubles couldn’t see them, the words, very well the words were too small*  One patient cited that individuals with dementia may have difficulty completing PROMs, while another felt similarly for individuals with learning disabilities  Related to physical functioning, this patient also reported needing more time because her dominant hand was injured, so she had to use her non-dominant hand: *“I’m not used to using my left hand so it just takes me longer to do.”* |
| 8 | Long et al (2021) | Caregiver | Patients with cognitive challenges had difficulty completing PROMs. One caregiver cited this as one of the reasons that he accompanies the patient to appointments and helps him complete PROMs  This patient noted difficulty concentrating while completing PROMs with his caregiver, stating that *“it was a lot”* and that it was *“too long to concentrate”* |
| 9 | Brochmann et al (2016) | Patients | One participant mentioned her visual impairment and explained that her son read the questions aloud from the questionnaires. |
| 10 | Baeksted et al (2017) | Patients | Patients reported problems related to sensitivity of the touch screen |
| 11 | Nielsen et al (2021) | Patients | One participant reported **cognitive difficulties** affecting her recall ability  *Then they ask ‘how have you been the last year?’, and then I have to sit there and say I do not remember. I only remember… I’m very in the moment… and maybe last week and then not much longer. I do not remember how I felt a month ago or how I was*  Two participants reported they were **dyslexic**, which meant that they found it more difficult to use a free text field  *I’m not good at… I’m dyslexic, I’m not good at formulating thing in writing. I just like to check boxes, and that’s really fine, that you are supposed to do*  *that in the questionnaire* |
| 12 | Grossman et al (2018) | Patients | One patient reported his poor eyesight made using the iPad difficult |
| 13 | Moradian et al (2018) | Patients | *A bigger screen for people who need glasses...someone like me* ***whose vision is affected*** *needs a bigger screen* |

**Finding #2:** As cited by some older adults and healthcare professionals, the lack of access to necessary digital technology (e.g., electronic devices and e-mail) hindered older adults’ capability to electronically complete PRMs. Similarly, older adults and healthcare professionals cited that having access to necessary digital technology (e.g., electronic devices and internet) facilitated older adults’ completion of PRMs electronically

| **#** | **Reference** | **Perspective of:** | **Extracted data relevant to finding** |
| --- | --- | --- | --- |
| **BARRIERS** | | | |
| 1 | Navarro-Millán et al (2019) | Patients | Not having access to a computer was cited |
| 2 | Long et al (2021) | Patients | Some patients did not have an email address, limiting access since PROMs in this clinic are sent via email |
| 3 | Sandhu et al (2020) | HCP | *A lot of my patients don’t use MyChart, they don’t have email, older lung cancer patients that live out in the country, they just don’t have that technology available. And even if they do, like a smartphone or a computer, they don’t feel comfortable. Just filling out a little survey with a mouse is hard* |
| **FACILITATORS** | | | |
| 1 | Kaur et al (2019) | Patients | Participants that owned either a portable (laptop or tablet) or desktop computer or smartphone, or a combination of the 3, were comfortable accessing the internet and checking electronic mail (e-mail) |
| 2 | Navarro-Millán et al (2019) | Patients | Patient cited access to a computer and internet service as a facilitator |
| 3 | Duman-Lubberding et al (2017) | HCP | Enhanced accessibility of OncoQuest for patients (e.g., ability for patients to access OncoQuest online from home) |

**Thematic category: DIGITAL TECHNOLOGY**

**User interface for older adults**

**Finding #3:** Older adults reported that having an easy-to-use, intuitive interface with the following features facilitated electronic completion of PRMs:

- large font and screen size
- visually appealing with colour, images and graphs
- clear labelling of response options and data
- indication of questionnaire progress (e.g., progress bar)
- ability to download or bookmark link to PROMs software on various electronic devices
- multiple questions per page

Healthcare professionals believed that an easy-to-use and visually appealing interface enables older adults to complete PRMs electronically.

Older adults not having to view all the questions at once when completing PRMs electronically was cited as a facilitator by healthcare professionals and administrative staff.

Some older adults and healthcare professionals believed that a touchscreen option facilitated older adults’ completion of PRMs electronically

| **#** | **Reference** | **Perspective of:** | **Extracted data relevant to finding** |
| --- | --- | --- | --- |
| **FACILITATORS** | | | |
| 1 | Aiyegbusi et al (2018) | Patients | *Simple, straightforward and easy to use*  *It is quite good really. It is easy enough*  *Clear and easy*  *Easy to use* |
| 2 | Kaur et al (2019) | HCP | The surgeons found the TickiT platform to be visually appealing, suitable for patients of all age groups, easy to use, and hence, ideal for routine use |
| 3 | Kaur et al (2019) | Patients | The ability to modify the font size was important to participants as was the ability to reorient the screen of the tablet or smartphone from landscape to profile view  The patients in this study preferred multiple questions per page, the presence of a completion bar, and minimalistic design (large black font against a light background and no distracting graphics). |
| 4 | Spaulding et al (2019) | HCP and front desk staff | Participants noted that the psychological aspect of not having to view all the questions (as with the paper format) can be less overwhelming for patients. In the ePSRM tool, questions are presented one at a time. |
| 5 | Spaulding et al (2019) | Front desk staff | *Before we would make packets for them and they'd be like oh my gosh I have to fill all this out? Which it's essentially the same thing on the iPad. But it doesn't stress them out as much because they can't see physically how much it is* |
| 6 | Spaulding et al (2019) | HCP | *There was an elderly guy who had mild cognitive impairment; he used to come with a case manager and caretaker. And they were convinced that he would have trouble [with the iPad], and he said, “Are you kidding me. Writing is so hard for me and here I just have to touch. I cannot even read my own handwriting … this way, I just have to touch.* |
| 7 | Delgado-Herrera et al (2017) | Patients | *Add descriptors “all of the time”; “most of the time”; “some of the time”; “a little of the time”; “none of the time” to numbers on the vertical axis*  *Include date of immediacy of need at the top of the table after “today” and delete the date column for the 24-hour report history (include date column for other report histories, eg, past week, past month)*  *Bold and underline “today”*  Overall, subjects found the mobile application easy to use |
| 8 | Delgado-Herrera et al (2017) | Patients | *Enable landscape view*  *Enable landscape view in order to improve readability (ie, font size and graphs)* |
| 9 | Long et al (2021) | Patients | Several patients noted a preference for tablets or desktop computers over smartphones owing to larger screens that make it easier to see. |
| 10 | Long et al (2021) | Patients | Several patients noted a preference for tablets over other devices owing to the ease of navigating PROMs via **tablet touchscreen** even in light of UE limitations or with their non-dominant hand |
| 11 | Snyder et al (2013) | Patients | Patients reported that the site was well-organized and laid-out  *I thought it was well organized and laid out*  Providing more explanation about the score meaning |
| 12 | Tolstrup et al (2020) | Patients | Patients reported that accessing and filling out the eHealth questionnaire was easy  *“I’m pleasantly surprised. I think it is really easy to deal with”* |
| 13 | Yamada et al (2020) | Patients | Majority of patients indicated that the user interface was intuitive and that the eAMS questionnaire was easy and straightforward to complete, with no training required |
| 14 | Samuel et al (2020) | Patients | Participants described positive experiences using the ePRO survey, including satisfaction with the ease of use  Participants generally reported that the symptom summary report was easy to understand and helped them better understand their symptoms  *It [understanding the Symptom Summary] was pretty easy for me* |
| 15 | Lehmann et al (2021) | Patients | Patients reported high satisfaction with the presentation of the results as bar charts  *bar charts are a good way of presenting the results.*  *I like that I can compare my results to those of other*  *patients with cancer.*  *I like that I can see my results after having completed*  *the questionnaire.*  *I like the design. The arrow in the results [direct link from the results to the self-management information] was very helpful.*  *...liked the color coding [red/green] as it was simple and easy to understand.* |
| 16 | Moradian et al (2018) | Patients | Some older participants (age>65) commented that they would prefer to use ASyMS on a device with a larger screen (with larger font size)  *bigger screen for people who need glasses...someone like me whose vision is affected needs a bigger screen*  *It is always nice for someone in my age if got a bigger text...Of course, if you could make it bigger, would be great.* |

**Finding #4:** Older adults reported that the following interface features hindered their ability to complete PRMs electronically:

- small font and screen size
- many scrolls to navigate through questionnaire
- use of certain display graphics (e.g., use of multiple colours, checkered boxes, circle options and smiley faces)
- unresponsive option and progress buttons
- unlabelled response options
- unnecessary pop-up of alerts
- mobile application downloading features
- drop downs were cumbersome

Some healthcare professionals cited that it was stressful for visually impaired older adults to click through multiple screens to complete PRMs electronically.

Caregivers and older adults experienced frustration when they could not progress through the questionnaire. Some healthcare professionals also cited that older adults experienced frustration with challenges in questionnaire progression.

Some older adults had difficulty using a touchscreen in absence of a touchscreen pen, due to the need to firmly press or for long periods with their fingers

| **#** | **Reference** | **Perspective of:** | **Extracted data relevant to finding** |
| --- | --- | --- | --- |
| **BARRIERS** | | | |
| 1 | Aiyegbusi et al (2018) | Patients | Beyond a certain point, the descriptions for the response options do not remain visible at the top for the group of KDQOL-36 questions that were set in a matrix format. The participants needed to scroll up to see the descriptions. This was an issue for those who struggled to use the mouse  *Can't see the options after a while*  Patients unintentionally omitted questions and assumed the progress buttons were not functioning when they could not proceed. The interviewer had to tell them to scroll up and check for omissions |
| 2 | Aiyegbusi et al (2018) | Patients | *The print is a bit small. That thing (mouse) is a bit fiddly to use*  *It (the fonts) could have been a bit bigger because you have got plenty of room on it* |
| 3 | Kaur et al (2019) | Patients | *You know the other one (smiley faces - TickiT) is fine for how your flight was. Did you like it? But going into a doctor’s office, you think it should be serious. And this (REDCap) is a little bit more clinical*  *I find these (square or circles) harder to read than something like that (emoticons). This, the circles, these in my mind do not line up with… I am empty, or I am full over here. That does not make a lot of sense to me. Same with this one. These squares I find really confusing*  *I do not like seeing how many more pages I have. It is just like oh, I have five more pages to go.*  The ability to either download or bookmark the link to the PROM software on an electronic device(s) was important to patients and was available in both REDCap and Tickit. Bookmarking the website link to the e-PROM made it easy to access, save, and return to later |
| 4 | Spaulding et al (2019) | HCP | Beyond the technology, nurses noted a major challenge due to the inability to edit the tool to reflect what patients want to convey.  *There are instances when the patient is taking too long, and I'll go out there and say let me help you … they are saying “but I'm selecting, but it's not.” they just keep tapping and it will not move onto the next question* |
| 5 | Delgado-Herrera et al (2017) | Patients | Difficulty downloading and/or installing the application onto their mobile devices  Patients expressed confusion as to the meaning of the ‘total score’ in the summary  Difficulty in knowing which questionnaire section was referred to by the ‘Daily Symptom Diary’ and ‘Symptom Event Log’ mobile application buttons  ‘Back’ buttons on the mobile application screens were too small and did not work as intended when pressed  Font was difficult to read when on a gray background (as on a mobile application button) |
| 6 | Delgado-Herrera et al (2017) | Patients | On some screens the font size was small, making it difficult to read clearly |
| 7 | Mou et al (2021) | HCP | *For elderly patients or people that are visually impaired and [PROs] can be stressful for them to click through all of the screens because it’s a lot of questions* |
| 8 | Long et al (2021) | Caregiver | Platform design presented difficulties including difficulty opening PROMs, advancing to subsequent PROMs, advancing to prior or subsequent questions within the same PROM, submitting responses, and navigating among multiple  questionnaires.  *it’s frustrating when you have to go in and out, in and out, you think you’re finished and then you get an email saying you’re not finished*  Another male caregiver shared:  *she would answer the questions and hit done, and it kept saying that it was incomplete like she didn’t answer a question.* |
| 9 | Long et al (2021) | Patients | Patient noted one questionnaire “where it didn’t like my answer” and she  was unable to submit for an unknown reason  *if you didn’t pay attention then you wouldn’t have known to go to the next survey.* |
| 10 | Tolstrup et al (2020) | Patients | A majority of the patients reported that the alert reminding them to contact the department popped up too frequently.  *If I were to call every time it pops up, I would have to call very often* |
| 11 | Tolstrup et al (2020) | Patients | Some of the patients, particularly the elderly, had a hard time using the touchscreen function with their fingers because they either pressed too hard or for too long. However, when they were given a touchscreen pen, which is  more accurate than the fingertip, they did not have any problems |
| 12 | Yamada et al (2020) | Patients | Patients reported using drop downs cumbersome  *Well I found that if you just fill out the information in the field, rather than use the drop downs that it worked much easier* |
| 13 | Samuel et al (2020) | Patients | *I just think that y’all need to let people, whenever you give them the surveys, the scale, explain it to ‘em exactly what it’s supposed to show you* |
| 14 | Moradian et al (2018) | Patients | Patients reported the need for a more advanced and attractive design, with better functionality and features in ASyMS to better address the needs of end users:  *Finding where everything is, it’s not labeled, so it would be easier if every option was labeled...I think it needs a higher-level menu, which may have to be categorized, which allows me to navigate around through it easily.*  *...add a search button. Having a search button just kills so much time. You can access the entire database in like 2 seconds. Saves a lot of time...I wish it had a*  *search button*  *I have an iPhone, which the text isn’t very much bigger than that, right? Like the text is the same basically. But the screen colors, like this screen color*  *to me (shows iPhone) is a lot easier to read than this (shows the ASyMS’s handset)* |

**User interface for healthcare professionals**

**Finding #5:** Some healthcare professionals valued an easy-to-use interface that helped them access questionnaires on various devices and browsers, with a visually appealing layout that used colour to draw attention to areas of concern and/or changes over time, to engage in the electronic collection and use of PRMs. Some perceived that difficulties in accessing the electronic system, lack of clear labelling of questions, the need for many clicks to access key information and limited visual presentation of data were barriers to electronic collection and use of PRMs

| **#** | **Reference** | **Perspective of:** | **Extracted data relevant to finding** |
| --- | --- | --- | --- |
| **BARRIERS** | | | |
| 1 | Amini et al (2021) | HCP | Impeding factors discussed as access to the web-based platform in the implementation process |
| 2 | Mou et al (2021) | HCP | *The frustration of even finding the PROs is pretty high. [Once I find the data] I can't even tell which [PRO question] is which because they're just all like shoved down there into one big blob of text. I wish there were a way to just visually tell like a like a good dashboard in your car if it’s a red light that means there’s something wrong.*  *Its hard to read—very small, and it is hard to follow the line across from question to answer’*  *Far too dense, clustered, poorly formatted* |
| 3 | Sandhu et al (2020) | HCP | *If I have to click a lot of buttons to get to it, it’s going to fade because it’s gotta be easy.... If you have to navigate the bowels of Epic to get to it, it’s not going to be useful*  *We would very quickly—in our notes—just pull [ePRO data] in the template so that it would pull and especially if it was like ‘dot [ePRO] recent’ and so it was the last three visits, for example, and it gave the raw scores next to each other and maybe a graph of the most severe or maybe it’s the total [score] that’s graphed* |
| **FACILITATORS** | | | |
| 1 | Kaur et al (2019) | HCP | The flexibility of accessing REDCap via desktop or tablet computers and smartphones (Windows/Macintosh/Unix/Linux) from a variety of browsers without the need to download additional software and the ability to export data to Excel or SPSS for analysis were cited as the key advantages of the REDCap platform. |
| 2 | Spaulding et al (2019) | HCP | Visual display of trends allows physicians to show patients the outcomes of their treatment and how their medical conditions and perceptions have changed over time  *I think it has helped some of my patients recognize how they are doing by showing them the outcomes of where their numbers are from where they started … many times when people are depressed, and they might improve but they still continue to feel somewhat miserable* |
| 3 | Mou et al (2021) | HCP | *[The PRO] questions should be formatted and asked in a way so that at a glance you can see [the] problems.*  *Important things [should be] in bold or color red flag serious results highlight positive answers* |
| 4 | Schick-Makaroff and Molzahn, (2017) | HCP | The nurses emphasized that their use of ePRO data was impacted by how the data were visually displayed.  *colors “gave me a quick glance of things I should address.”*  *“it jumps out at you more 'cause of the highlighting.”*  Some requested that data be displayed in one column (instead of two), and others wished that the font had been larger. The visual display of the results was very important to their ability to interpret results quickly and integrate them into their clinical assessments and follow-up.  They wished that ePRO data could be displayed longitudinally, in graphs, to see trends over time. |
| 5 | Snyder et al (2013) | HCP | Clinicians strongly preferred the graphic score reports in PatientViewpoint |
| 6 | Baeksted et al (2017) | HCP | Oncologists reported graphic presentation was clear and provide a good overview, while one oncologist suggested adding more pictures and/or  graphs of the patients’ symptoms |
| 7 | Sandhu et al (2020) | HCP | *Either if the report could be filtered to just select on moderate and severe versus all or somehow make less prominent the lower scores because you know I’m not sure that any score of 2 out of 10 is clinically meaningful*  *You could basically show the pre-op, immediate post-op, and long-term post-op symptom changes. That’s actually useful because one thing you’d like to do is you like to have a documentation of worsening or improving patient status* |

**Electronic device for PRMs completion**

**Finding #6:** Older adults generally were comfortable completing PRMs on portable electronic devices such as a laptop, tablet or smartphone, or on a desktop computer. Some older adults preferred using smartphones due to its screen colours and convenience to use the device, while some preferred a computer or tablet due to the larger screen size and reduced need for scrolling. A few older adults experienced difficulty in using a smartphone due to the side-to-side scrolls required.

| **#** | **Reference** | **Perspective of:** | **Extracted data relevant to finding** |
| --- | --- | --- | --- |
| **BARRIERS** | | | |
| 1 | Nielsen et al (2021) | Patients | There were some difficulties in achieving Digital Services that suits individual needs; one participant had tried to complete the questionnaire on a smartphone and did not find it suitable  *“I did actually sit down with my phone and did it. That’s a bad idea. It is actually quite difficult to read it then, because you all the time have to scroll the page from side to side to be able to read, I’ll say it is a good idea to be on the computer next”* |
| **FACILITATORS** | | | |
| 1 | Aiyegbusi et al (2018) | Patients | *Completing this was easy. On a regular basis it will be convenient to use a smartphone* |
| 2 | Kaur et al (2019) | Patients | The participants preferred completing the PROM on portable laptop, tablet, or smartphone or desktop computer |
| 3 | Long et al (2021) | Patients | Several patients noted a **preference for tablets** over other devices owing to the **ease of navigating PROMs via tablet touchscreen** even in light of UE limitations or with their non-dominant hand  Several patients noted a **preference for tablets or desktop computers** over smartphones **owing to larger screens** that make it easier to see |
| 4 | Tolstrup et al (2020) | Patients | The majority of patients were pleased with the tablet. |
| 5 | Yamada et al (2020) | Patients | Most participants stated that they would complete the questionnaire on their phone if they were given that option  Some would have preferred to complete the questionnaire on a larger device (eg, computer, iPad)  *I’d probably do it like 2 weeks before, considering I have access to my smartphone*  *I’m pretty competent on the internet and maybe on an iPad like on a larger device it might have been more free flowing* |
| 6 | Baeksted et al (2017) | Patients | Patients found the tablet computers easy to use. |
| 7 | Moradian et al (2018) | Patients | *I have an iPhone, which the text isn’t very much bigger than that, right? Like the text is the same basically.* ***But the screen colors, like this screen color***  ***to me (shows iPhone) is a lot easier to read*** *than this (shows the ASyMS’s handset) – preference based on screen colour on device* |

**Technical challenges**

**Finding #7:** Most older adults and healthcare professionals reported that technical challenges such as connectivity problems, device outages (e.g., going dark, logging out or system crashes), slow response time and device incompatibilities were barriers to electronic completion and use of PRMs.

| **#** | **Reference** | **Perspective of:** | **Extracted data relevant to finding** |
| --- | --- | --- | --- |
| **BARRIERS** | | | |
| 1 | Amini et al (2021) | HCP | Response time of the IT platform reported as barrier |
| 2 | Kaur et al (2019) | Patients | Participants reasoned that electronic data are vulnerable to system crashes and power outage |
| 3 | Spaulding et al (2019) | HCP | The main challenge with the implementation was with iPad use and technical glitches. Participants noted that internet connectivity was sometimes lost when moving from room to room, which frustrated patients |
| 4 | Delgado-Herrera et al (2017) | Patients | Steps to download were too difficult, took too long, or were incompatible with their personal phones |
| 5 | Mou et al (2021) | HCP | *[Patients] get frustrated with [the PRO tablets] if they log themselves out. They have to enter the encounter ID again… [which] they don’t remember.*  *Patients feel that the iPAD is buggy and hard to use*  *iPad is too slow/freezes* |
| 6 | Long et al (2021) | Patients | Device’s screen turned dark after being inactive for a certain amount of time, patients had difficulty returning to the questionnaire. |
| 7 | Baeksted et al (2017) | Patients | Patients reported technical problems, such as the tablet computer shutting down and having to start over or a slow responding tablet computer and having to touch each key more than once |
| 8 | Samuel et al (2020) | Patients | Automated telephone ePRO users noted barriers to completing the survey, such as technical difficulties with their cell phone reception or the automated telephone system was offline when respondents tried to call  *There was some sort of technical difficulty at… their end of it. Because it just went blank on me… where the phone call is made, and the beginning of the survey is just—it was like there was something wrong technically at the other end, because the phone just went dead* |

**Privacy and security of personal data**

**Finding #8:** Not knowing who could access personal and health data and what the data is used for, and exposure of data to malware viruses and hackers were cited as barriers to electronic completion of PRMs by most older adults. However, some older adults had no concerns about privacy risks when using their own device.

Healthcare professionals were concerned about data privacy and governance (e.g., who owns the data) when using certain software programs for patient data collection.

| **#** | **Reference** | **Perspective of:** | **Extracted data relevant to finding** |
| --- | --- | --- | --- |
| **BARRIERS** | | | |
| 1 | Kaur et al (2019) | Patients | *I would want to know who had access to it (PROM data) and what it was being used for. Like I would want to know those things before I decided whether I was going to complete it. And I think like I would only want the surgeon and key staff in his office to have access to it, and I think it should only be used for improving your surgical care, like your results…*  *For me, my legal name is different than my preferred name. Also, if my insurance is paying for the surgery, will the data be shared with them? I would like to know, if the surgery would be covered less, I mean by 50% less, depending on what I write in the survey*  The concerns regarding access to personal and health data by a third party (eg, employers, insurance companies) were especially heightened for participants who choose to conceal personal health information for privacy purposes  Participants reasoned that electronic data are vulnerable to malware virus(es) and hackers |
| 2 | Kaur et al (2019) | HCP | The surgeons were also concerned regarding the ownership and privacy of the data when utilizing the TickiT platform. |
| 3 | Yamada et al (2020) | Patients | Several participants expressed concerns about web-based security of questionnaire data  *Medical information, security is a big deal* |
| **FACILITATORS** | | | |
| 1 | Kaur et al (2019) | Patients | None of patients expressed concerns when specifically asked about the privacy risks associated with their own devices, such as loss of the device, saved passwords, or data. |

**Thematic category: SUPPORT FROM SOCIAL CIRCLE**

**Finding #9:** Older adults cited that support from family, caregivers, peers, healthcare professionals and healthcare services received in the form of help to understand and complete PRMs, support to use digital technology to complete PRMs, encouragement and reminders facilitated electronic completion of PRMs.

Healthcare professionals believed support from family and peers, while administrative staff believed helping patients (e.g., translation services for patients with language barriers) during completion of PRMs facilitated older adults’ completion of PRMs electronically.

One caregiver reported helping the older adult complete PROMs due to the cognitive challenges experienced by the older adult.

| **#** | **Reference** | **Perspective of:** | **Extracted data relevant to finding** |
| --- | --- | --- | --- |
| **FACILITATORS** | | | |
| 1 | Kaur et al (2019) | Patients | Participants preferred electronic reminders (text message followed by email), preferably at least 2 reminders to ensure optimal compliance |
| 2 | Spaulding et al (2019) | HCP | HCPs shared that “older” patients were likely to have difficulty, be hesitant, and had higher refusal rates compared with their younger counterparts. However, once the system was explained, some of the initial concern was eliminated.  *Once my older patients get some help from the front desk and realize that it's going to be a tap they, they seem to do well* |
| 3 | Navarro-Millán et al (2019) | Patients | Patients expressed that working with another patient with RA could help overcome their lack of familiarity with computers and electronic devices and assist in completing questionnaires about disease activity online  Participants expressed the desire to have an initial interaction in a structured, facilitated meeting or face-to-face group to establish trust before engaging in online communication with peers  Initiating social connections in person could help overcome reservations for sharing RA data electronically, the process of sharing symptoms and entering disease activity data in a PRO or electronic format  *This app would give me a* ***reminder*** *time when to take my medications, it would give me a way to communicate with my doctor via email, it would be a tool that if I am going through something I can talk into the phone and store this information and go back at a later date and review it*  *We show you, you know, how to access it through your email,” or we take your phone and say, “This is how you find this app. This is how you do it.” Have someone, whether it’s a receptionist, or a nurse, or somebody from the IT department, say, “Okay, this is the person who’s going to help the people who aren’t tech savvy access this stuff.*  *I have family support*  *I’m all about apps and stuff like that. But for people who aren’t, have someone in the office to show them, walk them through it step by step and make sure that they’re okay with it before they leave.*  *Well I have adult children and they taught me the art of communication through text messaging, “If you want to ask me a question text me,” and I did* |
| 4 | Long et al (2021) | Administrative staff | To support patients who are non-English speaking that need to complete questionnaires in English, clinic staff administer these questionnaires with the assistance of a live phone interpreter, as seen during observations |
| 5 | Long et al (2021) | Patients | One patient noted that when she has difficulty **understanding health-related information** in surveys she seeks out help from her mother, who “had been in the medical field before |
| 6 | Long et al (2021) | Administrative staff | Staff recounted situations in which patients directly told them that they needed help completing the PROMs due to inability to read. Staff also speculated that for patients who ask for help but do not specify why,  difficulty reading is likely a reason for a subset of those patients |
| 7 | Long et al (2021) | Caregivers | One caregiver cited that one of the reasons that he accompanies the patient to appointments and helps him complete PROMs was that the patient experienced cognitive challenges |
| 8 | Schick-Makaroff and Molzahn, (2017) | HCPs | The nurses believed that the process of completing the ePROs provided support to patients. In the clinic waiting rooms, completion of the ePROs sparked conversations between patients, and between patients and family members  *“Sometimes you see the dynamic in the waiting room where the spouse will be making comments, looking over their shoulder saying, ‘No, I wouldn't put that!’”* |
| 9 | Brochmann et al (2016) | Patients | One participant mentioned her visual impairment and explained that her son read the questions aloud from the questionnaires. Then she gave her  answers and her son filled out the questionnaires online.  *My son fills out the questionnaires for me* |
| 10 | Tolstrup et al (2020) | Patients | One patient could not do it and asked his wife to do the reporting following his instructions  It was not possible to send a text message reminding the patient to fill out the questionnaire on the relevant days. Two patients mentioned that a reminder text message would have been advantageous. |
| 11 | Yamada et al (2020) | Patients | More than half of the participants felt that receiving a reminder from the doctor’s office via email, text, or phone before their appointment would ensure that the questionnaire would be completed  *I honestly think if a person has an appointment booked, and then you send them an email saying your appointment is on such and such date, please fill out this questionnaire to improve the efficiency of your appointment, I can’t imagine people wouldn’t do that*  *If it’s a friendly reminder from you guys to fill out the questionnaire, of course someone like me, I’d be receptive and I’ll be like okay I’m being reminded to fill out, better go do it*  *Yeah so I’d get the email, if I try to do it right away if I can; if not I would definitely put maybe a reminder like if the questionnaire says have it done before a week before I’d put it in like two or three days before have it due so that I have it entered by the time I have to have it done.”*  Patients reported support from family would encourage them to complete questionnaires  *Gosh if my family members knew of it they would certainly encourage me to or push me to fill it in* |
| 12 | Yamada et al (2020) | Patients | Most participants described benefits to completing a patient questionnaire before their medical appointment, including that their primary care physician would have a more thorough understanding of their condition and that this would translate into better health care and disease control  *I think from the patient’s perspective, it would give them more insight as to what’s happening because they actually have to think about what their symptoms are and what they’re doing to help the asthma, so therefore, they can bring that information to the doctor.*  Patients reported that HCP would influence them to complete questionnaires  *Who? Well I suppose the doctor’s office if they sent out a text to me or something. I can’t, I don’t think anyone else would influence me.* |
| 13 | Samuel et al (2020) | Patients | *When I first started out, I didn’t understand some of the questions. Then I asked the nurse and she told me what to do. Then I was good to go* |
| 14 | Lehmann et al (2021) | Patients | Some patients expressed the wish for a reminder (email or text message) before the next appointment to complete the questionnaires online. |

**Thematic category: KNOWLEDGE AND SKILLS**

**Digital knowledge and skills**

**Finding #10:** Some older adults cited that having adequate knowledge and skills in using digital technology facilitated completion of PRMs electronically.

As cited by most older adults, administrative staff and healthcare professionals, older adults lacking adequate digital technology knowledge and skills made older adults feel uncomfortable and less confident when completing PRMs electronically.

| **#** | **Reference** | **Perspective of:** | **Extracted data relevant to finding** |
| --- | --- | --- | --- |
| **BARRIERS** | | | |
| 1 | Spaulding et al (2019) | HCP | HCPs shared that “older” patients were likely to have difficulty, be hesitant, and had higher refusal rates compared with their younger counterparts. However, once the system was explained, some of the initial concern was eliminated.  *Once my older patients get some help from the front desk and realize that it's going to be a tap they, they seem to do well*  HCPs reported patient's refusal to use the iPad |
| 2 | Navarro-Millán et al (2019) | Patients | Unfamiliarity with digital technology was cited. Those who expressed difficulty with technology indicated that having formal instruction or someone to assist or engage them in the electronic communication could empower them to consider this avenue |
| 3 | Long et al (2021) | Patients | Patients cited devices on which PROMs are administered (tablets, smartphones, laptops, or desktop computers) presented challenges for some patients  *When I first got the tablet in my hand, a little anxiety came over me because I’m not much into computers […] when you’re doing something you’re not familiar with, I get overwhelmed a little.*  One patient noted that because he had had a phone for < 1 year and did not feel comfortable with phone-based applications, he did not want to use it for PROM completion |
| 4 | Long et al (2021) | Administrative staff | Staff reported that elderly patients more often had difficulty completing PROMs due to low computer and/or technology literacy |
| 5 | Baeksted et al (2017) | Patients | Patients needed help at least for the first time they used the tablet computer |
| 6 | Nielsen et al (2021) | Patients | The one participant who did not use digital PROs reported that he did not know how to use computers |
| 7 | Grossman et al (2018) | Patients | Some patients reported concerns about adapting to or learning to use the interface  *I have a tablet at home, but I don’t use it right now…If I knew how to use it [the tablet] I could use [the PRO survey]* |
| 8 | Moradian et al (2018) | Patients | Patients did not receive any tutorial before and felt insecure about their actions and asked for assistance and approval before performing  tasks. Most participants mentioned that they would need some time to learn and get familiar with the ASyMS device before they could start to use it regularly  *If people use this a few times they will be able to (use and) navigate it easily* |
| 9 | Sandhu et al (2020) | HCP | *.. they just don’t have that technology available. And even if they do, like a smartphone or a computer, they don’t feel comfortable. Just filling out a little survey with a mouse is hard* |
| **FACILITATORS** | | | |
| 1 | Navarro-Millán et al (2019) | Patients | Patients cited familiarity with computers or smartphones as a facilitator  Working with another patient with RA could help overcome their lack of familiarity with computers and electronic devices and assist in completing questionnaires about disease activity online |
| 2 | Yamada et al (2020) | Patients | *No I don’t think so. It’s pretty straight-forward, you know we’re pretty computer tech savvy now*  *I’m pretty competent on the internet and maybe on an iPad like on a larger device it might have been more free flowing* |
| 3 | Nielsen et al (2021) | Patients | None of the participants who were assigned to use digital PROs reported any difficulties in using the digital system, indicating high ability to actively engage with digital services. |

**Health knowledge and literacy in general**

**Finding #11:** Older adults and healthcare professionals cited that older adults lacking adequate health knowledge found it difficult to understand medical terminology and health information in questionnaires, that made it challenging to electronically complete PRMs. Healthcare professionals cited that some of these older adults found difficulty in discerning between different health conditions they experienced.

Caregivers experienced difficulty in understanding medical terminology, which acted as a barrier to electronic completion of PRMs on behalf of older adults.

Administrative staff cited that low literacy levels in older adults limited their reading, hindering electronic completion of PRMs.

| **#** | **Reference** | **Perspective of:** | **Extracted data relevant to finding** |
| --- | --- | --- | --- |
| **BARRIERS** | | | |
| 1 | Spaulding et al (2019) | HCP | Patient's inability to comprehend the questions and content of the questionnaire was reported |
| 2 | Long et al (2021) | Patients | *Some people don’t know how to pronounce some of the words, so do they know what the meanings of them are? […] they don’t know exactly what it is […] or can’t read it*  Patients indicated difficulty with understanding “some of the health issues that may be listed” |
| 3 | Long et al (2021) | Administrative staff | *reading is likely a reason for a subset of those patients: “that is some of the main reason that [patients] don’t want to do [the questionnaires], because their reading is limited.*  Some patients can read but read slow suggests that it is not only illiterate patients who cannot complete PROMs, but also that there may be a low literacy segment who have varying levels of difficulty |
| 4 | Long et al (2021) | Caregivers | Caregivers indicated difficulty with understanding “medical terminology” |
| 5 | Baeksted et al (2017) | HCP | One oncologist stated that patients could find it difficult to separate the  gradings ‘severity’ and ‘influence on daily activity’  *Too many similar questions. But the right thing to do—for both patients and doctors.* |
| 6 | Nielsen et al (2021) | Patients | Participants in this study found it difficult to rate their own pain levels, particularly over time  *I do not think it is easy… but I do answer of course. Everything is… It does not hurt as much so you’re not able to walk…. And then you think… how do you modulate that… I’m able to go to work, I’m able to walk… It is not like that it affects me in a way where I’m not able to work. I imagine others feeling that way… so if I have to modulate it, then my score is rather low. Also compared to how you can feel…. When I was sick.* |
| 7 | Nielsen et al (2021) | Patients | The PRO system included a free text field, which challenged patients who found it difficult to articulate information about their health in writing, reflecting a deficit in understanding of health concepts and language  *I would prefer talking to a doctor. Most likely. Because there is always something, some questions, some thoughts, that, when you sit in front in a consultation, will appear. I think it is like that, if you have a comment or something in the end of the questionnaire, you do not always get it written, and how are you supposed to write something like that. It is difficult to express feelings. It is easier to look someone in the eye.* |
| 8 | Grossman et al (2018) | HCP | *Some patients don’t recognize that abdominal bloating is fluid retention, or that nocturnal cough is orthopnea. They don’t link their diet to gaining weight. When people put those together they can better self-manage* |
| 9 | Sandhu et al (2020) | HCP | *Some of the terms like fatigue versus tired, fatigue versus dyspnea, shortness of breath can be difficult for patients to discern between. So, if you walked up four flights of stairs and at the end are you tired or short of breath? Some people are both* |

**PRMs interpretation knowledge and skills**

**Finding #12:** Most healthcare professionals reported the lack of knowledge and skills to interpret PRMs responses and address problem areas hindered electronic collection and use of PRMs.

| **#** | **Reference** | **Perspective of:** | **Extracted data relevant to finding** |
| --- | --- | --- | --- |
| **BARRIERS** | | | |
| 1 | Kaur et al (2019) | HCP | The surgeons also identified lack of knowledge about identifying an appropriate, validated PROM for a clinical condition, scoring, and interpretation of the data. The surgeons identified gaps in understanding how to collect and analyse data in a methodologically rigorous manner |
| 2 | Mou et al (2021) | HCP | *“When [PROs] started there was not a great… education of the primary care community in terms of what they were going to be and the best workflow… to use them. The worst thing is occasionally I get a message in my [EHR] in-basket that one of my patients answered, [indicating] that they wanted to kill themselves… I don't believe primary care has been trained enough in terms of what to do with those answers.* |
| 3 | Schick-Makaroff and Molzahn, (2017) | HCP | The nurses’ lack of familiarity with the KDQOL-36 necessitated that  they spend more time with it, and sometimes that time wasn’t available.  *If I had two things to look at, I'm going to choose the one that I'm familiar with because it takes me more time to figure that [KDQOL-36] out.* |

**Regular exposure enhancing health knowledge**

**Finding #13:** Older adults and healthcare professionals perceived that completing PRMs electronically helped improve older adults’ health knowledge and awareness of their own health condition in the long term.

| **#** | **Reference** | **Perspective of:** | **Extracted data relevant to finding** |
| --- | --- | --- | --- |
| **FACILITATORS** | | | |
| 1 | Schick-Makaroff and Molzahn, (2017) | HCP | *I think it brings an awareness to them in that moment and makes them think about each individual thing so it's like right at the front of their brain when they go in to talk to the nurse.*  *Well I think it’s always beneficial for the patients to be aware of their health and what they can do and make changes to improve it. I mean having them involved and seeing them, writing down a score, I think it’s another (pause) visual so they can say, I feel that - I think that helps them understand. I mean it’s like getting your lab work, you know, you see the numbers.”* |
| 2 | Tolstrup et al (2020) | Patients | The majority reported that their attention to side effects was heightened due to the intervention (eg, “*Your focus is increased because you have to remember to write it”*  More of the patients also found that filling out the questionnaire made it easier to remember symptoms when they came to the clinic. |
| 3 | Tolstrup et al (2020) | HCP | The clinicians agreed that the patients were better prepared when they came to the outpatient clinic, and that the patients had increased focus on their symptoms and were more alert:  *I think it is an advantage that the patients become more aware of the side effects that can occur* |
| 4 | Baeksted et al (2017) | Patients | *You become more aware of what happens to you. You get an opportunity to*  *follow the changes in the symptoms* |
| 5 | Baeksted et al (2017) | HCP | *The patients are better prepared and have thought about the symptoms they have experienced since the last visit* |
| 6 | Samuel et al (2020) | Patients | Participants described several benefits to completing the survey, including gaining knowledge about their disease and better awareness of their symptoms |
| 7 | Nielsen et al (2021) | Patients | Digital PROs enhanced their knowledge about their disease and their understanding of health concepts, which again affected their engagement with their health and healthcare  *Some of the questions asked in the questionnaire have I never received before through ten years of consultations. […] I just thought; that is how it is. Until that questionnaire came. It is… something about… I think it was sore spots on the skin or something. Like when you touch me, you don’t have bruises, but it*  *just hurts those places, that is also the bowel disease, that I have never thought, I just thought it is because I am a little weak.”* |
| 8 | Grossman et al (2018) | Patients | *It made me broaden my thinking, and it kind of brought together what [my doctors, nurses, and nutritionist] keep telling me. It gave a different perspective*  Connects symptoms with disease  *Symptoms come up that, before you saw them, you didn’t realized they were connected with [your disease]* |

**Rationale for PRMs collection and use**

**Finding #14:** Older adults and healthcare professionals reported that older adults feeling obliged to complete PRMs for healthcare professionals without understanding the rationale for PRMs collection was a barrier to electronic completion of PRMs.

| **#** | **Reference** | **Perspective of:** | **Extracted data relevant to finding** |
| --- | --- | --- | --- |
| **BARRIERS** | | | |
| 1 | Kaur et al (2019) | HCP | *Patients won’t necessarily understand the value of gaining information in any environment, because of cosmetic surgery, just like everything else we should be following their outcomes... A lot of cosmetic patients, I know, however, think well don’t learn on me. So, they kind of feel like they are paying you money and they do not have to be involved in the unpleasant academic or questioning field* |
| 2 | Mou et al (2021) | HCP | *I've had many, many patients tell me ‘Doc why am I filling this out?’. I’ve had a couple tell me they feel like I'm giving them an exam like a like a school exam*  *and it makes them nervous and anxious and they don't know why I'm asking these questions. [They say] ‘Doc all I'm here for is sinus infection but you're asking me all these questions about how my depression’… It gets in the way of the patient really feeling like they're engaging with me.* |
| 3 | Yamada et al (2020) | Patients | Patients reported if they are being forced to complete the questionnaire, they will, but believed it is not their responsibility  *No I don’t think it’s my responsibility…. if it’s a must than I will, you know, will be forced to do that but I don’t think it’s my responsibility* |
| 4 | Duman-Lubberding et al (2017) | HCP | The value of repeated use is not clear to patients |

**Thematic category: MOTIVATION AND INCENTIVES FOR CAPTURE AND USE OF PRMs**

**Older adults’ motivation and incentives**

**Finding #15:** Older adults reported the following factors as motivators for electronic completion of PRMs:

PRMs utilisation:

- informing health data to healthcare professionals for care planning
- helping advance health research, and help other patients improve health outcomes through research advancements
- healthcare professionals reviewing, discussing and using PRMs responses during consultations to improve care
- self-monitoring of condition based on PRMs responses

Owning responsibility:

- owning responsibility to improve one’s own health and awareness of their health

Trust in healthcare service:

- trust in healthcare provider (e.g., hospital asked to use digital PRM system)

| **#** | **Reference** | **Perspective of:** | **Extracted data relevant to finding** |
| --- | --- | --- | --- |
| **FACILITATORS** | | | |
| 1 | Kaur et al (2019) | Patients | Participants reported that completing e-PROMs before clinic visits would help prepare for the visit by identifying meaningful and important appearance or HRQOL issues |
| 2 | Navarro-Millán et al (2019) | Patients | Patients expressed motivation to collect data if they were used to manage symptoms or obtain support from their physician  Patients expressed interest in the data collection platform allowing them to learn about RA and RA medications including side effects, and learn about nonmedical (i.e. self-management) options for treating RA.  *I think it’s great because you get to see other people and – and talk to them and hear how they dealt with theirs and let for instance me a year and a half I’ve had it and known about it. And I don’t really have anybody to talk to. So I don’t know what they’re doing and what they have done. I know of a few people that – that have it and when I first was diagnosed I did call one girl that I know, but I don’t see her regularly and interact with her*  Participants were interested in providing data to physicians |
| 3 | Snyder et al (2013) | Patients | *I think that quality of life is a very important issue and having doctors bring these issues up at appointments is helpful* |
| 4 | Tolstrup et al (2020) | Patients | Many of the patients explained that a strong motivation for entering the study was that they would be able to help future patients. Of course, they believed that they themselves would benefit, but being able to help others was also important |
| 5 | Yamada et al (2020) | Patients | Older adults’ owning responsibility to improve own health:  Completion of the questionnaire was viewed by participants as important and a priority  *I feel it is [my responsibility] because the way I look it, the more information my doctor has about what’s going on with me, the better he or she is able to help me manage my symptoms and cope*  A small number of participants felt that completing the questionnaire would not help them to understand their asthma nor lead to better asthma control  *The fact that the information is helping the doctor work with me to create a treatment plan that reduces or eliminates my asthma condition or symptoms and overall affect on me. That would definitely influence me to continue to answer these questions*  *If the system sees the need for me to do this in order to get the healthcare that I need, and therefore, I would do it* |
|  | Yamada et al (2020) | Patients | Most participants described benefits to completing a patient questionnaire before their medical appointment, including that their primary care physician would have a more thorough understanding of their condition and that this would translate into better health care and disease control  *I think from the patient’s perspective, it would give them more insight as to what’s happening because they actually have to think about what their symptoms are and what they’re doing to help the asthma, so therefore, they can bring that information to the doctor.*  Patients reported that completing the questionnaire may lead to better asthma management/asthma control  *Oh, I think definitely. I know if there was something around like this years ago when my asthma was out of control, I think it would have really helped to hone in on where, what’s the problem*  Patients reported that completing the questionnaire will help to inform the asthma action plan  *Well I think this one would probably be right at the top because if it’s information that my doctor needs in order to let’s say help put my asthma action plan together, or if he wants to sit down and have a detailed discussion with me as to what should be in my asthma action plan and all that. Once the doctor has all that information in front of him or her, I feel that whatever answers are in that questionnaire could be properly used to set up an asthma action plan*  *The fact that the information is helping the doctor work with me to create a treatment plan that reduces or eliminates my asthma condition or symptoms and overall affect on me. That would definitely influence me to continue to answer these questions* |
| 6 | Baeksted et al (2017) | Patients | *The dialogue with the oncologist is more efficient. The questions are about relevant problems. You can see where symptoms differ from the last treatment, such as new side effects*  *You are the one who knows best and feels the side effects. You can give a more complete picture.* |
| 7 | Samuel et al (2020) | Patients | *I would choose to [continue completing it] because … It’s gonna help science* |
| 8 | Nielsen et al (2021) | Patients | All of the participants who received digital PROs as a replacement for consultations responded that they believed that the primary purpose was to save money by having fewer physical consultations at the hospital. Most  of them perceived this to be a good thing, as they thought the hospital should direct their resources either to other patients who needed them more, or to themselves when they felt worse  *To save time, I guess […] It is ok with me […] if it can save some money, then they can use the money when I come in all sick*  Digital PROs enhanced their knowledge about their disease and their understanding of health concepts, which again affected their engagement with their health and healthcare  *Some of the questions asked in the questionnaire have I never received before through ten years of consultations. […] I just thought; that is how it is. Until that questionnaire came. It is… something about… I think it was sore spots on the skin or something. Like when you touch me, you don’t have bruises, but it*  *just hurts those places, that is also the bowel disease, that I have never thought, I just thought it is because I am a little weak.”*  *It is not something I just do. I do take it seriously, and I make sure that I’m in a closed environment, that I’m able to have 100% focus, because it is after*  *all my gut. It is not just for some sort of statistic…”* |
| 9 | Nielsen et al (2021) | Patients | Most participants reported that their primary motivation for using the digital PRO system was that the hospital had asked them to. Participants reported a high degree of trust in, and a good relationship with, the clinic  *You got to trust it. I think I do that, and it is only because I hope… this department has always been good… I hope it is still really good*  Patients trusted that clinicians read their PRO data before a consultation, reflecting that participants felt safe and in control of their data  *I do not experience that they ask about the things I wrote […] But that I assume. I don’t know, of course, I don’t ask if they have read it, but that I assume*  *I guess it is to get the information, to be prepared and to get a holistic view—also backwards to see, if there is anything to see, when you get enough questionnaires filled out* |
| 10 | Grossman et al (2018) | Patients | *“I’d like to use this system at home … to keep track of my symptoms.”* |

**Finding #16:** As cited by some older adults and healthcare professionals, older adults were demotivated to complete PRMs electronically if they believed it did not influence their health and the care they received, and when their healthcare professional did not review, discuss and use PRMs responses during their consultations.

Some older adults reported that a negative reaction to the PRMs responses by their healthcare professional discouraged them from completing PRMs electronically.

| **#** | **Reference** | **Perspective of:** | **Extracted data relevant to finding** |
| --- | --- | --- | --- |
| **BARRIERS** | | | |
| 1 | Kaur et al (2019) | HCP | Surgeons discussed most patients who attend outpatient cosmetic surgery clinics either pay out-of-pocket or have third-party insurance. This may result in attitudes that hinder compliance with the e-PROMs. Patients who pay out-of-pocket for procedures are under no obligation to provide official names or contact information and may feel less obligated to participate in research or quality improvement initiatives  *Patients won’t necessarily understand the value of gaining information in any environment, because of cosmetic surgery, just like everything else we should be following their outcomes... A lot of cosmetic patients, I know, however, think well don’t learn on me. So, they kind of feel like they are paying you money and they do not have to be involved in the unpleasant academic or questioning field* |
| 2 | Navarro-Millán et al (2019) | Patients | Participants were interested in providing data to physicians, but they also expressed discouragement when physicians did not attend to the information they provided at the point of care. Participants emphasized that if they shared information with the provider, whether at home or at the point of care, they wanted physicians to act on this information. If physicians did not incorporate the provided data, patients were far less interested in completing questionnaires at home |
| 3 | Snyder et al (2013) | Patients | Patients reported negative feedback included questioning whether their provider looked at the results, noting that the intervention could be impersonal, and indicating that the score reports only identified issues that were already known.  *I don’t even know if my doctor looked at my surveys so I am not sure if it helped or not. It didn’t bring up anything new for me.*  *The results seemed to highlight things that were already known to be an issue. It didn’t say anything new.* |
| 4 | Tolstrup et al (2020) | Patients | A minority did not know if their reports had been seen by the clinician:  *I think they have seen it (the report), but it is not something we have discussed*  A few believed that the clinician had in fact not seen it at all, which was of course frustrating due to the fact that they had spent time filling out the questionnaire |
| 5 | Yamada et al (2020) | Patients | *I don’t see it as something that’s, you know useful, you know for the doctor or myself cause he would already have that on his profile about me*  Patients were unsure whether the results would be reliable to be useful  *I think for average people, I’m not sure that the results would be reliable to be so very useful. I imagine that regardless of what’s on the survey, that the doctor would verify all of it with me again anyway* |
| 6 | Samuel et al (2020) | Patients | *It just didn’t seem like it was an advantage or a disadvantage. Just another form to fill out but it didn’t have much impact on my life, to be honest* |
| 7 | Nielsen et al (2021) | Patients | Some patients were unsure how this additional information was used  *I did think, maybe they are starting to take these issues seriously. A whole picture of the well-being of the person they actually start to be aware of. And*  *then I think it is positive. But if they do not intend to use it for anything, then of course I think it is not worth much*  *“I do not think I gain anything from it [talking to the healthcare professionals]. I do not think they can tell me anything […]. It [the disease] is something I live with, and I will never get over it, so of course it is to my benefit to know as much as possible.* |
| 8 | Nielsen et al (2021) | Patients | Some participants were reluctant to use the questionnaire as a self-service, because they knew that nurses would check their answers and react on them  *If you could go in and pull out some charts. You can’t use it like that. As soon as you go in and fill out a questionnaire, then a larger process is starting*  *where someone has to sit and read the questionnaire and assess it… if it is one thing or another. But I do like charts.* |
| 9 | Lehmann et al (2021) | Patients | Patients also explicitly reported a decline in motivation toward the end of the study, caused by a lack of sufficient feedback from physicians who did not discuss their PRO results during the consultations. |
| 10 | Duman-Lubberding et al (2017) | HCP | A lack of feedback from the surgeon to the patient on the results of OncoQuest may lead to a decrease in patient’s motivation to use OncoQuest |
| 11 | Grossman et al (2018) | Patients | *I’ll do [mi.Symptoms] if you promise to read it. That’s the problem, [the doctors] don’t read. You tell one doctor one thing and then the next set of doctors come and you have to start all over again.”* |

**Healthcare professionals’ motivation and incentives**

**Finding #17:** Healthcare professionals reported the following factors as motivators to electronic collection and use of PRMs:

PRMs utilisation:

- involvement of patients to voice their perception of health through PRMs completion
- PRMs responses informing quality improvements and research advancements
- collaboration with multi-disciplinary care teams to improve care based on PRMs responses
- shared decision making and tailored care as a result of PRMs use

PRMs data completeness:

- PRMs response data completeness

Enhanced clinical documentation:

- enhanced documentation based on PRMs responses

Regulatory directives:

- regulatory directives (e.g., PRMs data as part of data registries) mandating PRMs data collection

Access to PRMs responses:

- immediate access to PRMs responses and meaningful presentation of PRMs responses for clinical decision-making

| **#** | **Reference** | **Perspective of:** | **Extracted data relevant to finding** |
| --- | --- | --- | --- |
| **FACILITATORS** | | | |
| 1 | Amini et al (2021) | HCP | PRMs utilisation:  Collaboration with multiple disciplines on every level of care was discussed as an encouraging factor  Engaging patients in their own care process seems to incentivise healthcare professionals to implement PROMs in clinical care, which subsequently may lead to a more individualized health service delivery and an improvement in the quality of care  Involvement of patients in own care and tailored care as a result of PROMs use were reported as facilitators |
| 2 | Kaur et al (2019) | HCP | PRMs utilisation:  For research or specific practice-related questions, the cosmetic surgeons unanimously preferred REDCap because it is easy to design the survey, collects data anonymously, and provides the ability to conduct unlimited surveys.  *…I will tell you there’s one possibility is that you find out you’re not as good as you think you are. And you’re getting worse outcomes. I suppose we might prefer calling it the “ostrich approach.” I do not want to know, because maybe*  *I am not as good as I think I am. Ideally, if we learned that we are not as good as we think we are, then we would respond by trying to improve our results. Until we have this [PROs in practice] like if I have no quality improvement*  *analysis at all, I am not going to know how my outcomes are. So, I must admit I am working in a void right now. It would, of course, it would be useful...* |
| 3 | Spaulding et al (2019) | HCP | Enhanced clinical documentation:  They found that the new ePSRM creates uniformity in how notes are generated  PRMs utilisation:  It's more of a shared decision‐making process as opposed to just one‐way street …. I used to draw pictures for the patient that there's like graphs and diagrams; I don't have to do that anymore  PRMs utilisation:  Physicians also reported that the ePSRM has increased direction of clinical evaluation and care, thus it provides them with a more focused assessment of the patients' needs during visit  *I think from a psychiatric perspective, it has made psychiatric review of systems more elaborate. The second is it has helped focus the interview more on the problems that patient has identified* |
| 4 | Mou et al (2021) | HCP | Enhanced clinical documentation:  *My favorite is the annual wellness visit because all the pertinent questions can get pre-populated into the notes. That’s incredibly helpful*  PRMs utilisation:  *Sometimes the patient will point out something [on PROs] that they normally wouldn't have just brought it up… so there has been occasion where a headache will come up or abdominal pain… That does add to the visit experience*  *I briefly look at [the PROs to make sure any outstanding questions like any metrics are not grossly abnormal like the GAD 7 or PHQ9… if those scores*  *are very high, I will perhaps dig in a little harder.*  *I'll say [to my patient], ‘hey you mentioned you had joint pain and some weight changes, can you tell me about that?’ So, I will absolutely bring that up in the in the discussion, based on what I see [in the PRO data].* |
| 5 | Krawczyk et al (2019) | HCP | PRMs utilisation:  *I think [formally assessing quality of life and care experiences] is super useful in our setting*  *Well, just the ultimate of better patient/family care.*  *Satisfaction, job satisfaction. To know that you assessed your patient properly and were able to act on it.*  *And maybe to see patient outcomes that you’ve implemented a certain type of treatment—oh my goodness, it’s working.*  *Patient’s feeling better.*  *For those who want another way to voice their experiences, it’s fantastic because a lot of people . . . you know, by the time they kind of come to us within their journey of health care and transitioning through the disease process, a lot of people don’t feel like they’ve been listened to.*  *Because we’re using this data to help us do our job better to help our patients and families, but it’s also going in another direction for possible research, audit and all these other things* |
| 6 | Schick-Makaroff and Molzahn, (2017) | HCP | PRMs data completeness:  *When they’re sitting in the waiting room, filling in [the ePROs], they can be a bit more honest with themselves and then that is very revealing to the healthcare providers*. |
| 7 | Schick-Makaroff and Molzahn, (2017) | HCP | Regulatory directives:  The nurses admitted that they spent more time focusing on the ESAS-r:Renal (rather than the KDQOL-36) because they were familiar with it and it was required to be part of the health authority data registry.  Access to PRMs responses:  Many nurses said that they looked at the ePRO data **immediately** so that they could focus on the priorities or concerns of the patient. Nurse 5 said, “I look at it [ePROs] before I start the interview. I focus on it first -  those are the things that are important to the patient, rather than focus on what's important to me.” Keeping the patient “in the middle of the visit” was a way for nurses to “be patient-centred.”  PRMs utilisation:  The nurses saw the ePROs as “tools” that helped them focus their nursing assessment.  *The ePRO results “trigger us, as nurses, to dig into it a bit more, ask more questions, and maybe investigate a little bit more.”*  As the nurses narrowed their clinical assessment, the ePRO data helped them identify areas that otherwise might have been missed. *“I might not ever ask them about something that was rated really high on the survey…if I hadn't seen that, I probably would not have focused in on that one item.”*  Access to PRMs responses:  The nurse clinicians explained that their use of the ePRO data was impacted by whether or not the data was integrated with their workflow. They wished that ePRO data could be displayed longitudinally, in graphs, to see trends over time. Instead, they found themselves reading through charts to gain historical perspective. “*You kind of like to have a general long-term picture, not just like*  *one, 20-minute interview”* |
| 8 | Snyder et al (2013) | HCP | PRMs utilisation:  Clinicians reported that the intervention helped them identify and address issues that might have otherwise gone unnoticed, made patients more engaged in their care, and enabled standardized tracking of patients’ PROs.  *…it showed me that she was having more symptoms of depression than she had been reporting to me during her visits.*  *It contributed to my communication with the patient about BODY ISSUES she’s having that I wouldn’t have known to ask about but they were flagged in her results.*  *I felt that he was more engaged in the treatment by taking the surveys.*  *we adjusted her treatment schedule and dosing to address the issues that she raised.*  Access to PRMs responses:  HCPs preferred the ease of access of the plain-text score reports within the EHR |
| 9 | Baeksted et al (2017) | HCP | PRMs utilisation:  *You get a better impression of the patient’s overall situation—symptoms*  *and side effects*  *It is positive that the patients rate themselves—sometimes we underestimate. But the dialogue is important for specifying the [symptom and severity]* |
| 10 | Duman-Lubberding et al (2017) | HCP | PRMs utilisation:   - HCPs reported an enhanced insight into patients’ HRQOL - Attention for a wide range of symptoms and concerns (including psychosocial issues) - The possibility to improve (timely) referral to supportive care tailored to individual patients’ needs - The opportunity to offer increased (profound) attention to patients - Offering patients the opportunity to have their story heard |
| 11 | Sandhu et al (2020) | HCP | PRMs data completeness:  Oncologists reported data completeness across most or all patients was a facilitator to use PROMs data  Access to PRMs responses:  *Either if the report could be filtered to just select on moderate and severe versus all or somehow make less prominent the lower scores because you know I’m not sure that any score of 2 out of 10 is clinically meaningful*  *You could basically show the pre-op, immediate post-op, and long-term post-op symptom changes. That’s actually useful because one thing you’d like to do is you like to have a documentation of worsening or improving patient status*  *It would be most helpful to know what’s the key thing that needs to be addressed with the patient that day. Many patients have a slew of complaints, and what are the ones that you really need to focus in on before you go in* |
| 12 | Sandhu et al (2020) | HCP | Enhanced clinical documentation:  *When I go to bill, I can bill for severe fatigue, pain, and all these other things. So, I think that helps overall.* ***It helps the quality of the documentation because it translates a score into a diagnosis*** *and it also helps the billing because it translates, ‘I paid attention to something’ into ‘We’re getting reimbursed for it.’”*  *An expandable list of symptoms with highlighted positive responses. So, that you could go from the complete list and then you could compact that to the positives. Because the phrase you would like to use is ‘All 14 review of systems have been requested and the pertinent positives include the following* |

**Finding #18:** Healthcare professionals reported that the following factors demotivated them to electronically collect and use PRMs:

PRMs utilisation:

- believing that PRMs did not influence the care they provided
- non-discipline specific PRMs collection and use
- believing PRMs collection and use disrupts patient care duties

Discrepancies in patient’s assessment of health:

- discrepancies in health assessment between the patient and themselves

PRMs data completeness:

- PRMs responses providing insufficient information
- non-completion of PRMs questions and low response rates

Regulatory directives:

- Regulatory directive to collect and use PRMs, in absence of clear messaging of rationale and benefits for collection and use of PRMs

Healthcare professional’s age and length of service:

- healthcare professional characteristics associated with older age, higher length of service and not reviewing PRMs regularly

Access to PRMs responses:

- lack of immediate access to and meaningful presentation of PRMs responses for clinical decision-making

| **#** | **Reference** | **Perspective of:** | **Extracted data relevant to finding** |
| --- | --- | --- | --- |
| **BARRIERS** | | | |
| 1 | Amini et al (2021) | HCP | PRMs data completeness:  Insufficient information and inappropriate organisation of care reported as barriers |
| 2 | Kaur et al (2019) | HCP | Healthcare professional’s age and length of service:  *We are past the stage of high engagement with the electronic stuff that younger people will have..* |
| 3 | Spaulding et al (2019) | HCP | Healthcare professional’s age and length of service:  Adoption amongst physicians was also dependent on their level of professional experience.  *My guess is most younger physicians are probably going to be comfortable, but people who are towards the, end of their career probably won't be very open to making a whole lot of changes* |
| 4 | Mou et al (2021) | HCP | PRMs utilisation:  *Maybe if… we got into the habit of [reviewing] our patients’ questionnaires… we would use them more*  *[PROs] quite rarely truly impact [my patients’] clinical care… it’s not a huge factor in how I'm going to manage them.*  *My suspicion that most providers don't even look at [PROs]… they [take] time away that they could have been doing something else.* |
| 5 | Mou et al (2021) | HCP | Regulatory directives:  *A lot of these screening questions we have to ask anyway because it’s a regulatory requirement… Can you imagine if you only have eight min with the doctor and the doctor has to do [all] this? I know you're here for a sinus infection but let me ask you this: Have you ever had thoughts of suicide?* |
| 6 | Mou et al (2021) | HCP | PRMs utilisation:  *We are screening for things for which we have nothing to offer. We don't have*  *resources to help with education, housing, safety and so on*  *We need to really think about data we can do something with!* |
| 7 | Snyder et al (2013) | HCP | Access to PRMs responses:  *I tried to look but when I looked in EPR only her older scores were there. I didn’t have time to then go to try to look on the website before meeting with her.*  *I don’t think that the EPR information was helpful. It has no context.* |
| 8 | Tolstrup et al (2020) | HCP | Discrepancies in patient’s assessment of health:  There was some discrepancy between how the patient and the clinician graded a given symptom. In some cases, the clinician did not find the symptom to be as severe as the patient. In other instances, the clinician felt that the patient had in fact neglected a symptom that they believed should have been reported:  *“sometimes there’s a discrepancy between what you find out when you talk to the patient and what has been reported … the two things supplement each other*” |
| 9 | Baeksted et al (2017) | HCP | Discrepancies in patient’s assessment of health:  Oncologists reported the discrepancies in rating of symptoms by patients  *The patient’s self-reporting gives a picture of the patient’s overall condition, but the patient’s answers were not necessarily related to the chemotherapy*  *Patients rate their symptoms differently than we [the oncologists – ed.] would do, and patients often underestimate or overestimate the severity of their*  *symptoms.* |
| 10 | Duman-Lubberding et al (2017) | HCP | Access to PRMs responses:  The availability of OncoQuest outcomes during consultation is hampered due to difficulties to trace OncoQuest results at their computer screen |
| 11 | Sandhu et al (2020) | HCP | PRMs data completeness:  Oncologists expressed concerns that patients would not complete the ePRO survey before their visit. The two participants who previously used ePROs reported low response rates from their own personal experience, which deterred them from viewing PROs as part of their workflow, even for patients who completed the survey |
| 12 | Sandhu et al (2020) | HCP | PRMs utilisation:  *Give you a global sense of how [patients] are doing and—for what I [a radiation oncologist] do—there’s not a lot of actionable items. I think it might be very different for [a medical oncologist] who’s giving chemo. We approach our field a little bit more like in a proceduralist type of way where we focus on a discrete problem. So, for me, if it’s not addressing that problem, it’s ancillary data*  *Doing surgery is far more billable than writing notes.... So I suspect that surgeons’ response about PRO documentation will be very different in internal medicine or clinical oncology doctors* |

**Acceptance of intervention by older adults**

**Finding #19:** Administrative staff cited that acceptance of electronic completion of PRMs by older adults motivated them to support the process of collecting PRMs electronically.

| **#** | **Reference** | **Perspective of:** | **Extracted data relevant to finding** |
| --- | --- | --- | --- |
| **FACILITATORS** | | | |
| 1 | Spaulding et al (2019) | Front desk staff | Most front desk staff noted that adoption depended upon acceptance from all parties involved, patients, staff, and providers, being comfortable with the system. Staff adoption was also dependent upon orientation that getting patients to be satisfied is the ultimate goal. Once patients are satisfied, staff satisfaction will increase.  *Really just encouraging them that if the patient is satisfied with this program then, patient satisfaction equals our satisfaction I believe* |

**Location of PRMs administration**

**Finding #20:** Older adults found completing PRMs questions and reviewing responses from home or remote location (outside the clinic) was convenient and facilitated electronic completion of PRMs.

The need to spend extra time in the clinic to complete PRMs, incurring transport and parking costs to be physically present in the clinic, hygiene and data privacy risks associated with using shared devices and feeling rushed were barriers to completing PRMs electronically in the clinic, as reported by most older adults.

Incomplete questionnaires due to limited time for older adults to complete PRMs in the clinic was reported as a barrier to electronic collection and use of PRMs by some healthcare professionals. Some healthcare professionals perceived having an in-clinic option to complete PRMs may facilitate older adults’ PRMs completion rates

| **#** | **Reference** | **Perspective of:** | **Extracted data relevant to finding** |
| --- | --- | --- | --- |
| **BARRIERS** | | | |
| 1 | Kaur et al (2019) | Patients | Utilising the computer at the clinic was susceptible to infection or hygiene risks and unprofessional behavior by other patients (eg, access to inappropriate content or accidentally accessing data from the previous user) |
| 2 | Mou et al (2021) | HCP | *[patients] don't arrive more than a few minutes early to their appointments, so*  *[PROMs] actually can harm [clinic] flow*  *The average wait time for a patient between walking in the door and being [placed in the exam room] is about 90 seconds… So, what ends up happening is a lot of the time [is that] I'll look in the room and the questions are only half done… I end up going through some of the questions myself.* |
| 3 | Long et al (2021) | Patients | Patients who completed PROMs in clinic felt that they had insufficient time. One patient noted that she may have answered the questions differently if she had completed the PROMs at home, where she would not feel rushed  *I’m not as fast as some… before they called me, I was running a little behind time, so I had to catch up, so the process got speeded up a little*  Related to physical functioning, this patient also reported needing more time because her dominant hand was injured, so she had to use her non-dominant hand: *“I’m not used to using my left hand so it just takes me longer to do.”*  One patient said that at home she can “*really read through the questions”*  and “*really understand what you’re answering*,” and that “*sometimes at the clinic your name might be called before you finish the process, and you might feel like you’re being rushed”* |
| 4 | Samuel et al (2020) | Patients | Patients reported following barriers to consider when designing an ePRO system:  *I live two hours’ drive. I can’t get no transportation* |
| **FACILITATORS** | | | |
| 1 | Aiyegbusi et al (2018) | Patients | Study suggests that patients with advanced CKD may find the Renal ePROM system easy to use and acceptable for reporting their symptoms remotely |
| 2 | Kaur et al (2019) | Patients | *Yeah, I think I would do it at home. I think because of the convenience factor. I could do it anytime I want. When completing at the clinic, for example, will my doctor be present when I’m doing this? So again, not that I would skim over it, but my responses might be a little bit more positive or negative depending on how emotionally charged I am. Because there’s always going to be some sort of emotional connotation with these kinds of questions. So, I think at home you’re more likely to be your true self* |
| 3 | Brochmann et al (2016) | Patients | There was agreement among the participants that the opportunity to complete the questionnaires at home ensured time and calmness to consider what to answer |
| 4 | Snyder et al (2013) | Patients | *“I think this is a good idea especially for people who tend to forget in between appointments what was going on and what they want to tell the doctor when they see him. This kind of takes care of the remembering for you. Being able to leave comments for the doctor from home is good too, especially since Dr. X told me that he saw them! That was nice.”* |
| 5 | Yamada et al (2020) | Patients | *I think it’s more relaxed which makes the patient feel easier and there’s no rush. Like okay, you’re doing the questionnaire and somebody comes to the door or you can just go answer the door, and then go back to the questionnaire, or be doing the questionnaire at 3 o’clock in the morning if you want* |
| 6 | Samuel et al (2020) | Patients | Some preferred completing ePRO surveys at home because there would be **fewer interruptions and more time to focus on the survey**.  *It was probably more convenient at home to do it on your time, plus you could stop, come back, finish if somethin’ came up or somethin’ you had to do*  Other preferred completing ePRO surveys in clinic because they could do so **during their appointment wait time and/or ask staff questions in person**.  *…I liked it better when I was in the clinic ’cause I already had that time to do it*. |
| 7 | Nielsen et al (2021) | Patients | Some participants felt that avoiding hospital appointments (for example when asymptomatic) was the main benefit of the digital PRO system  *It fits me well that I do not have to drive 100 km back and forth to go to the hospital. That being as long as everything is as it is right now* |
| 8 | Grossman et al (2018) | Patients | Two participants, unprompted, asked for mi.Symptoms access at home to aid self-tracking of symptoms - Facilitates personal tracking and monitoring  *I’d like to use this system at home … to keep track of my symptoms.* |
| 9 | Sandhu et al (2020) | HCP | Respondents recommended having an in-clinic backup option electronically with tablets or kiosks to increase response rate |

**Timing and frequency of electronic PRMs collection**

**Finding #21:** Some older adults reported that completing PRMs at a regular frequency (e.g., once a week or once a month) and close to the day of consultation facilitated electronic completion of PRMs. Infrequent completion of PRMs that led to no changes in health, and that reduced familiarity with the electronic system for PRMs completion were cited as barriers to electronic completion of PRMs by some older adults.

Healthcare professionals cited that the lack of PRMs completion closer to the day of consultation was a barrier to electronic collection and use of PRMs.

| **#** | **Reference** | **Perspective of:** | **Extracted data relevant to finding** |
| --- | --- | --- | --- |
| **BARRIERS** | | | |
| 1 | Yamada et al (2020) | Patients | Patients reported that completing the questionnaire when visiting the HCP once a year to manage the condition does not make a big difference in the management of symptoms  *Not sure I see why that would be case because the choice of going once a year to see Dr. [X], he knows what I’m taking, he asks pertinent questions and then adjusts the medications as he feels is appropriate*  *If I have the choice not to fill it, I will not fill it out, you know. I would maybe fill it out one time, that one time that my doctor should have that on his profile* |
| 2 | Lehmann et al (2021) | Patients | Infrequent appointment schedule and completion of PROMs electronically was a barrier to patients remembering to use the portal |
| 3 | Duman-Lubberding et al (2017) | HCP | Patients’ usage of OncoQuest **after the follow-up visit t**o the surgeon (due to logistic reasons) leads to a lack of (accurate) feedback from the surgeon to the patient and to a possible delay in referral to supportive care |
| 4 | Sandhu et al (2020) | HCP | *If you have somebody fill it out seven days ahead of time and they’re acutely depressed and we don’t know it until we see it, you could be legally liable for not acting upon that*  *That patient population under treatment, things change from day to day, hour to hour, their side effect profile. So, them filling out a screener tool a week before I see them, everything could have changed within a week* |
| **FACILITATORS** | | | |
| 1 | Brochmann et al (2016) | Patients | Participants assessed it as acceptable to fill out the questionnaires once  a month |
| 2 | Tolstrup et al (2020) | Patients | It was not possible to send a text message reminding the patient to fill out the  questionnaire on the relevant days. However, this did not constitute a problem for the patients, who found it easy to remember because they were doing it on a fixed weekday - *regular frequency made it easier for patients to remember to do this*  The patients reported that the number of items and the length of the questionnaire were appropriate and that reporting on a weekly basis was fitting |
| 3 | Yamada et al (2020) | Patients | Patients reported they are more willing to complete questionnaires close to day of appointment  *if my appointment is in a month, maybe I would do it. Maybe if my appointment was in 10 months, I wouldn’t do it*  *I would try hard to do it the day before the appointment.”*  *I’d probably do it like 2 weeks before, considering I have access to my smartphone* |

**Access to additional resources based on PRMs responses**

**Finding #22:** Most older adults and healthcare professionals reported that access to additional medical or non-medical resources (e.g., self-management guidance or social support) based on PRMs responses encouraged older adults to electronically complete PRMs.

Some healthcare professionals reporting lack of resources to offer (e.g., education, social services), and some older adults reporting inability to access resources (e.g., getting questions answered from healthcare professional or personalised information) based on PRMs responses were barriers to electronic collection and use of PRMs

| **#** | **Reference** | **Perspective of:** | **Extracted data relevant to finding** |
| --- | --- | --- | --- |
| **BARRIERS** | | | |
| 1 | Mou et al (2021) | HCP | *We are screening for things for which we have nothing to offer. We don't have*  *resources to help with education, housing, safety and so on*  *We need to really think about data we can do something with!* |
| 2 | Samuel et al (2020) | Patients | *I think any of those things, especially barriers to getting symptoms diagnosed. Barriers to getting questions answered. Getting help from the provider. Those are probably the most important.* |
| 3 | Nielsen et al (2021) | Patients | *first I thought, well, you know your life line sort of disappears, but then again, I know the phone is there.”*  Participants perceived their ‘life-line’ to be direct support from their healthcare provider. Even when in stable remission, participants talked about their fear of not having the support in case of flare-ups |
| 4 | Nielsen et al (2021) | Patients | While few participants had trouble with the digital aspect of the digital PRO system, others would have liked it to be even more advanced in order to quantify their disease status and serve their individual needs  *If I compare with my stepfather, he got diabetes, one tool he got is that he can pierce himself in his finger and then he takes this glucose test, and it helps him to adjust his medicine, and it gives him an idea of how he is feeling, if he cannot feel it otherwise. I can’t do that. I wish I could. Then I all the time have to feel. I’m bad at that* |
| 5 | Moradian et al (2018) | Patients | *It would help if you could customize to the individual...the specific therapy they are receiving...There is one major thing, that is the food. One of the problems in my experience, I went through two different regimens of chemotherapy, different elements of the regimens have different food requirements, some for example do not allow caffeine or alcohol or meat products or spices. None of that is here, that would be helpful if me and my wife are about to contemplate dinner and it tells us can I eat such and such, those answers would be helpful in*  *terms of my chemotherapy. This app tells us about chemotherapy in general, whereas different patients have different regimens of chemotherapy...* |
| **FACILITATORS** | | | |
| 1 | Kaur et al (2019) | Patients | Participants hypothesized that completing PROM scales about psychosocial quality of life may help in verbalizing feelings, resulting in increased access to additional resources |
| 2 | Mou et al (2021) | HCP | *I find I always review [PROs] and use it as a jumping off point [for questions]… especially for the Social Determinants of Health to make sure that we’re identifying patients that could benefit from extra resources.*  *I think the social determinants of health is very useful. We now have social workers and different programs to support patients.* |
| 3 | Navarro-Millán et al (2019) | Patients | Patients expressed interest in accessing educational resources for medical and nonmedical symptom management  Several participants were interested in learning from others with RA about available online resources, how to better use electronic or online resources available for patients with RA, and the best ways to communicate with their doctors  Several participants identified a need for resources for accurate and tailored medication information, provided in lay terms, that includes evidence of long-term effects of RA medication and potential drug interactions  Participants expressed interest in learning about symptom and medication management through platforms similar to those with which they communicated data to their providers. They expressed that having this information would be a motivator for them to enter responses to electronic questionnaires through platforms, more so if their provider also requested that they complete the questionnaires  Participants expressed that resources on RA management can help them overcome their feelings of isolation, which at the same time could serve as a motivation for them to engage in electronic data collection as well |
| 4 | Tolstrup et al (2020) | HCP | HCPs reported there was a better chance that the patients would react appropriately by contacting the department in time instead of waiting for the next scheduled consultation, which might be days or weeks ahead.  *It may be precisely the group of patients who are not good at self-care or at least some of them…the weakest patients who…will benefit most from self-reporting by being guided into becoming more aware of when to react to symptoms* |
| 5 | Lehmann et al (2021) | Patients | Patients provided positive feedback on the self-management tools |
| 6 | Duman-Lubberding et al (2017) | HCP | The possibility to improve (timely) referral to supportive care tailored to individual patients’ needs |
| 7 | Grossman et al (2018) | Patients | Facilitates personal tracking and monitoring  *I’d like to use this system at home … to keep track of my symptoms.* |

**Thematic category: EMOTIONAL EXPERIENCE**

**Finding #23:** Some older adults reported that answering questions about their health conditions made them emotionally elevated, encouraging them to complete PRMs electronically. Some healthcare professionals believed that older adults may feel less embarrassed to electronically complete PRMs especially related to personal and sensitive topics (e.g., related to mental health, sex life and social determinants) rather than talk about them initially, which may facilitate older adults’ completion of PRMs electronically.

Some older adults and healthcare professionals cited that emotional distress caused by having to respond to questions on health conditions, and personal and sensitive topics were barriers to older adults’ completion of PRMs electronically. Anxiety caused by the limited time available to complete PRMs in the clinic was reported as a barrier to electronically completing PRMs by some older adults.

| **#** | **Reference** | **Perspective of:** | **Extracted data relevant to finding** |
| --- | --- | --- | --- |
| **BARRIERS** | | | |
| 1 | Aiyegbusi et al (2018) | Patients | Study discussed how patients accompanied by their partner, paused significantly when answering questions on burden to family, sex life (KDQOL-36) and feelings of depression |
| 2 | Mou et al (2021) | HCP | *[Regarding SDOH questions], some of the people are upset about it because they think they're being profiled as someone who might have food insecurity.* |
| 3 | Long et al (2021) | Patients | Feeling rushed in clinic to complete PRMs in a limited time caused anxiety for some patients |
| 4 | Snyder et al (2013) | Patients | *It made me feel bad answering [sexual function] every time when the situation won’t ever change while I am on these medications so why keep asking about it? It just reminded me of that loss and made me feel bad every time* |
| 5 | Tolstrup et al (2020) | Patients | One patient reported that she was reminded of her disease every time she  responded |
| 6 | Nielsen et al (2021) | Patients | Some participants found that their anxiety increased when they were asked about symptoms they did not have, but might expect to get  *Well… there are some symptoms I have never had. They just keep popping up again and again in the questionnaire. But then again it is not targeted me,*  *it is targeted for a group of patients. Then the answer is simple, I guess, but it is that thing about, when you have never had that symptom before, then maybe you have had without knowing. But I don’t think I have, but you… it makes you… That’s the hard part, well, now I say the hard part, it is also where you are grateful, that there are symptoms that you do not have* |
| 7 | Duman-Lubberding et al (2017) | HCP | OncoQuest may address unsolvable problems (e.g. dry mouth, swallowing problems) possibly leading to a decrease in patients’ motivation to use OncoQuest |
| **FACILITATORS** | | | |
| 1 | Schick-Makaroff and Molzahn, (2017) | HCP | The nurses believed that it may have been easier for the patients to initially complete the ePROs rather than talk about the items, especially for challenging topics such as depression, anxiety, sex, or pain.  “*maybe they're embarrassed a little bit or it's easier to put it on a screen maybe than talk about it?”* |
| 2 | Grossman et al (2018) | Patients | *I think it would be really good have [mi.Symptoms] as a conversation starter, because a lot of times, the doctor will ask you many questions,* ***and…when the doctor asks them some people feel intimidated***  *Answering these questions about my symptoms lifts me up, and I feel like I’m not alone* |

**Thematic category: OLDER ADULTS’ AUTONOMY**

**Finding #24:** As cited by some older adults and healthcare professionals, the influence from family, caregivers, administrative staff or healthcare professionals sometimes led to the suppression of the older adults’ voice, discouraging older adults to complete PRMs electronically.

| **#** | **Reference** | **Perspective of:** | **Extracted data relevant to finding** |
| --- | --- | --- | --- |
| **BARRIERS** | | | |
| 1 | Aiyegbusi et al (2018) | Patients | The noticeable hesitation by a participant during their test session, which was attended by their partner, raises the issue of external influences on the information patients may provide especially if completing the Renal ePROM at home |
| 2 | Spaulding et al (2019) | HCP | *I can think of two patients that had some problems with it, and I think one patient was already sort of extremely unhappy about being referred for psychiatric evaluation* |
| 3 | Krawczyk et al (2019) | HCP | HCPs articulated that use could further person-centered palliative care by minimising clinical assumptions about patients, including family members in the circle of care  *I can’t remember how many times we come into rounds and we’re making an assumption about how a person is perceiving themselves, in rounds, we do that a lot .. and family, I think that’s a huge piece too* |
| 4 | Brochmann et al (2016) | Patients | One participant mentioned that her son filled out the questionnaires due to impaired vision. This demonstrates a dilemma; it is valuable that patients who are unable to fill out PROs themselves are supported by their relatives, but PROs filled out by relatives may be influenced by the relatives’ opinions. |
| 5 | Yamada et al (2020) | Patients | Patients reported if they are being forced to complete the questionnaire, they will, but believed it is not their responsibility  *No I don’t think it’s my responsibility….if it’s a must than I will, you know, will be forced to do that but I don’t think it’s my responsibility* |

**Thematic category: PATIENT – HEALTHCARE PROFESSIONAL COMMUNICATION**

**Finding #25:** Structured communication during consultations (e.g., discussion of results focusing on problem areas and changes over time), enhanced patient – healthcare professional interactions and patient involvement in voicing their perception of health were reported as facilitators to electronic collection and use of PRMs by some older adults and healthcare professionals.

Some older adults and healthcare professionals reported that PRMs completion made the patient – healthcare professional interactions impersonal, discouraging the electronic completion of PRMs. Some older adults feared that relying on electronic systems to communicate about health may lead to the replacement of face-to-face consultations, and lack of support from healthcare professionals and the healthcare service.

| **#** | **Reference** | **Perspective of:** | **Extracted data relevant to finding** |
| --- | --- | --- | --- |
| **BARRIERS** | | | |
| 1 | Mou et al (2021) | HCP | *I've had many, many patients tell me ‘Doc why am I filling this out?’. I’ve had a couple tell me they feel like I'm giving them an exam like a like a school exam and it makes them nervous and anxious and they don't know why I'm asking these questions. [They say] ‘Doc all I'm here for is sinus infection but you're asking me all these questions about how my depression’… It gets in the way of the patient really feeling like they're engaging with me.* |
| 2 | Krawczyk et al (2019) | HCP | *I think it [quality of life and experience of care assessments] should be done maybe by either social worker or spiritual care coordinator, people that can sit down and talk about that, because I would feel that I’m not compassionate enough . . . Sometimes you ask one patient or one family member one question that requires a yes or no answer and they would go into like 10 minute conversation, which also pushes you back from what’s waiting for you out there, the bells are ringing and stuff. I cannot relax in knowing that my other people are having pain and I’m sitting here listening to a life story that has nothing to do with a patient. And I’ve learned, it’s actually a learned skill, to kind of interrupt the conversation and say, “Oh, so sorry. I just need to run.” But some of them are really hard. If somebody’s really upset, struggling with like husband or father of three young kids dying, then . . . I just rather not put myself into those shoes that I ask the question and then I can’t fulfill that, so I’d rather not even initiate that conversation, because I know if I do, and maybe it’s not the best practice, but I feel if I start, I need to finish, and I can’t, and then I feel really bad . . . [and] when I leave, I have to put that smile back on and go to next room and pretend that nothing happened in the next room*  *I do think that in nursing in general that we tend to rely too much on tools and protocols and not enough on personal communication and just quality assessments. And in my heart I think although these are validated tools, I think they’re validated under certain conditions. Like for the ESAS for instance, a quality tool that’s definitely been validated, but I think that [we use it] in a way that is not validated, for instance, and accreditation would be one reason . . . I don’t think that any tool should be something that’s a mandatory thing . . . And I think actually a lot of these tools are robbing nursing of the art of nursing . . . I can explore these things in a conversation that is far less clinical, probably more inviting to the person—although at times I think probably they would like the more anonymity of just interacting with a piece of paper or a tablet—but I find that I can do an assessment myself, talking to a patient, seeing their facial cues, you know, their body language and things like that, and get more out of it than somebody simply circling a scale of 0 to 10* |
| 3 | Snyder et al (2013) | Patients | Patients reported negative feedback included questioning whether their provider looked at the results, **noting that the intervention could be impersonal**, and indicating that the score reports only identified issues that were already known.  *Overall I don’t feel it was very helpful. It feels impersonal, just felt like extra paperwork that I had to do. I don’t know how useful the questions are. They only seemed to ask about things that we talk about during the appointments anyway* |
| 4 | Tolstrup et al (2020) | Patients | One third had the feeling that they contacted the department more as a result of the reporting. Thus, the majority of patients did not think that they were more in touch with the hospital due to the reporting |
| 5 | Samuel et al (2020) | Patients | *I think any of those things, especially barriers to getting symptoms diagnosed.* ***Barriers to getting questions answered. Getting help from the provider.*** *Those are probably the most important.* |
| 6 | Nielsen et al (2021) | Patients | Some participants still preferred physically attending the clinic for personal interaction  *Maybe it is very troublesome to drive in there, but I prefer the personal conversation with real people*  It was important to participants that digital PROs were not used as a replacement for synchronous or face-to-face consultations if needed. A need  for supplementary support from healthcare providers was expressed:  *first I thought, well, you know your life line sort of disappears, but then again, I know the phone is there.*  Some participants had concerns about being lost in the system after being assigned to the digital PRO system, therefore perceiving the digital PROs as a barrier to interaction with their healthcare providers  *It took a while before I got one [a PRO questionnaire]. It is almost… I think it is a year after we discussed it, that I got one. Why, I don’t know. But then again, they had not promised that it would be fast. But it took a long time. I did come to think I was forgotten*  Patients raised concerns about technology making disease management and communication more difficult |
| **FACILITATORS** | | | |
| 1 | Amini et al (2021) | HCP | A more structured patient-provider communication about PROM results was another reported facilitating factor  Better preparedness/ more information on how patients are doing prior to consultation  Involvement of patients in their own care was reported as a facilitator |
| 2 | Kaur et al (2019) | Patients | The surgeons felt that adding PROMs did not entirely replace interactions with patients. However, it targeted the consultations to patient concerns and helped track patient outcomes over time |
| 3 | Spaulding et al (2019) | HCP | Participants reported increased engagement between patients and care team, which was viewed positively  HCPs discussed that the electronic PRM format lends itself to more interactive conversational sessions with patients. For example, physicians are able to view and review patients' responses before meeting with patients that enhances their interaction, spend less time on interview, and spend more time for comprehensive discussions on treatment plans with patients. It also improved feedback to patients.  *It's more of a shared decision‐making process as opposed to just one‐way street … I used to draw pictures for the patient that there's like graphs and diagrams; I don't have to do that anymore*  Visual display of trends allows physicians to show patients the outcomes of their treatment and how their medical conditions and perceptions have changed over time  *I think it has helped some of my patients recognize how they are doing by showing them the outcomes of where their numbers are from where they started … many times when people are depressed, and they might improve but they still continue to feel somewhat miserable*  Physicians also reported that the ePSRM has increased direction of clinical evaluation and care, thus it provides them with a more focused assessment of the patients' needs during visit  *I think from a psychiatric perspective, it has made psychiatric review of systems more elaborate. The second is it has helped focus the interview more on the problems that patient has identified*  *I can just turn the screen towards the patient and they can just look*  *at it and physician and the patient both can clearly identify most pertinent symptoms. I say to my patients ‘Alright, all these red symptoms that are displayed as such seems like are your main concern. Is that so?’*  *I'm not a different physician today than I was 2 years ago. I really didn't change that much; my practice style didn't change much … but my patient satisfaction has improved dramatically* |
| 4 | Mou et al (2021) | HCP | *Sometimes the patient will point out something [on PROs] that they normally wouldn't have just brought it up… so there has been occasion where a headache will come up or abdominal pain… That does add to the visit experience* |
| 5 | Krawczyk et al (2019) | HCP | *For those who want another way to voice their experiences, it’s fantastic because a lot of people . . . you know, by the time they kind of come to us within their journey of health care and transitioning through the disease process, a lot of people don’t feel like they’ve been listened to.* |
| 6 | Navarro-Millán et al (2019) | Patients | Participants expressed a great deal of interest in augmented communication with RA health care providers in real time or soon after symptoms arise. Most participants preferred phone or email communication, with few reporting use of a patient portal or electronic medical record (EMR) messaging. Common reasons for reaching out to providers were flare symptoms, medication refills, questions about medication, or requesting an appointment sooner  *I want to be able to communicate with him but not just drive him crazy … I am going to tell him the most important things and give him time to make the arrangements to try and help me because I’m not the only patient*  All participants expressed the belief that their health care provider was their most trusted source of health information and treatment recommendations, and they preferred to learn about RA from their provider |
| 7 | Schick-Makaroff and Molzahn, (2017) | HCP | *I think it’s improved probably the interactions that we have in our clinic when*  *these patients are, you know, filling out their surveys and are getting these visual tools …you can’t help but look them over and over and address things.* |
| 8 | Snyder et al (2013) | HCP | Clinicians reported that the intervention helped them identify and address issues that might have otherwise gone unnoticed, made patients more engaged in their care, and enabled standardized tracking of patients’ PROs.  *…it showed me that she was having more symptoms of depression than she had been reporting to me during her visits.*  *It contributed to my communication with the patient about BODY ISSUES she’s having that I wouldn’t have known to ask about but they were flagged in her results.*  *I felt that he was more engaged in the treatment by taking the surveys.* |
| 9 | Snyder et al (2013) | Patients | *It was a place where you had a chance to talk about things that the doctors don’t usually talk to you about. I remember that my mood answers changed a while back… it was a good place for me to mention … since it would not have come up during the appointment otherwise.*  *I think this is a good idea especially for people who tend to forget in between appointments what was going on and what they want to tell the doctor when they see him. This kind of takes care of the remembering for you. Being able to leave comments for the doctor from home is good too, especially since Dr. X told me that he saw them! That was nice.*  *I had never thought to bring up the BODY IMAGE issues with my doctor because I didn’t really think that they were “medical” things. I didn’t think it was anything he would want to know about. If he hadn’t asked about it, we would never have talked about it. I am glad we did though. It was reassuring.* |
| 10 | Tolstrup et al (2020) | Patients | When the patients came to the outpatient clinic, two out of three of the patients who were interviewed felt that the health care professionals had in fact seen their reports and included them in their consultation:  *“It is like having an agenda for a meeting”*  “*It makes you feel as if you are not just a number in the system”*  Overall, the reporting made the patients feel more involved in their treatment and care:  *“It is nice that we have something common to talk about”* |
| 11 | Yamada et al (2020) | Patients | Most participants described benefits to completing a patient questionnaire before their medical appointment, including that their primary care physician would have a more thorough understanding of their condition and that this would translate into better health care and disease control  *I think from the patient’s perspective, it would give them more insight as to what’s happening because they actually have to think about what their symptoms are and what they’re doing to help the asthma, so therefore, they can bring that information to the doctor.* |
| 12 | Baeksted et al (2017) | Patients | *The dialogue with the oncologist is more efficient. The questions are about relevant problems. You can see where symptoms differ from the last treatment, such as new side effects*  *You remember things to talk about with the doctor* |
| 13 | Baeksted et al (2017) | HCP | *You get a better impression of the patient’s overall situation—symptoms*  *and side effects.*  *The patients are better prepared and have thought about the symptoms they have experienced since the last visit* |
| 14 | Samuel et al (2020) | Patients | *I think it probably made me think more concisely about symptoms. When I did talk to the doctor, I could express what was going on better.*  *It just brought up some talk points that maybe I wouldn’t have thought of in there—you’re emotional about having cancer and answering the questions, and I probably would have forgot some of those things, the talk topics* |
| 15 | Nielsen et al (2021) | Patients | Most participants identified that using digital PROs informed and enhanced their face-to-face consultations  *I guess it is to get the information, to be prepared and to get a holistic view—also backwards to see, if there is anything to see, when you get enough questionnaires filled out*  The participants felt motivated to engage with digital services, due to these different ways in which the digital technology could be used as a means to interact with healthcare providers |
| 16 | Duman-Lubberding et al (2017) | HCP | The possibility to improve (timely) referral to supportive care tailored to individual patients’ needs  The opportunity to offer increased (profound) attention to patients  Offering patients the opportunity to have their story heard |
| 17 | Grossman et al (2018) | Patients | Prepares patient for interaction with provider  *mi.Symptoms reminds the person what they want to talk about [with the physician]: in the 10 minutes we have together, let’s make sure that we’ve addressed these things.*  *I think it would be really good have [mi.Symptoms] as a conversation starter, because a lot of times, the doctor will ask you many questions, and…when the doctor asks them some people feel intimidated* |

**Thematic category: WORKFLOW**

**Efficiencies, time constraints and changes to work routines**

**Finding #26:** Facilitators to electronic collection and use of PRMs reported by some healthcare professionals were improved clinic efficiency through the use of electronic systems to screen PRMs responses and determine patient needs prior to the consultation, reduced clerical time due to patients independently completing PRMs electronically, enhanced EHR documentation based on PRMs responses and automatic distribution of questionnaire, and time saved through focussed patient assessments based on PRMs responses.

Some older adults and administrative staff reported that knowing healthcare professionals save time using the electronic system to access and use PRMs encouraged engagement with electronic completion of PRMs.

Barriers to electronic collection and use of PRMs cited by many healthcare professionals were time constraints and new routines associated with incorporation of PRMs in care and use of electronic systems to access PRMs responses, inundation of PRMs response data resulting in having too many things to discuss with the patient, delays to clinic workflows associated with challenges in PRMs administration (e.g., questionnaires not working or patients not arriving early to complete PRMs in time before the appointment), challenges with seamless data access and additional work created by having to cooperate with other multi-disciplinary healthcare professionals.

| **#** | **Reference** | **Perspective of:** | **Extracted data relevant to finding** |
| --- | --- | --- | --- |
| **BARRIERS** | | | |
| 1 | Amini et al (2021) | HCP | Time constraints of care providers in combination with the labour-intensive nature of the incorporation of PROMs in care were frequently reported as barriers  Learning new work routines with PROMs |
| 2 | Kaur et al (2019) | HCP | *.. I am sort of also looking at there’s extra time on me, there’s extra money and at the end, have we improved outcomes? I know there’s a noble drive for it, for any given patient to give them the best outcome they can have. That sort of more along an ethical call for us. But in practice, that’s against the realities of trying to churn people through your office, because there’s a lot of work to be done.* |
| 3 | Spaulding et al (2019) | HCP | The major unintended consequence included physicians having too many items to discuss with patients, especially if they responded positively to all items on the ePSRM  *Now they have answered about these 20 to 30 items, so I have to ask them, “Alright, so what do you mean by when you say that I have too much stuff lying around the house? And I've collected so much that it has become a problem”, so I have to screen them for hoarding Before, that‐ that may not have happened … So, yes, so there is something to be said about too much information.”* |
| 4 | Mou et al (2021) | HCP | *A lot of these screening questions we have to ask anyway because it’s a regulatory requirement… Can you imagine if you only have eight min with the doctor and the doctor has to do [all] this? I know you're here for a sinus infection but let me ask you this: Have you ever had thoughts of suicide?*  *I could say 25% of the time [the PROs] don't work. I know my front desk staff hates them [because it delays clinic flow]. My front desk staff would argue that it less efficient.*  *The average wait time for a patient between walking in the door and being [placed in the exam room] is about 90 seconds… So, what ends up happening is a lot of the time [is that] I'll look in the room and the questions are only half done… I end up going through some of the questions myself.*  *[patients] don't arrive more than a few minutes early to their appointments, so [PROMs] actually can harm [clinic] flow* |
| 5 | Krawczyk et al (2019) | HCP | HCPs cited increasing workloads, a lack of resources for efficiently sharing results and addressing patient and family member concerns once identified, discomfort in dealing with emotional distress, and/or belief that these issues are primarily the professional domains of social work and/or spiritual care  *I think it [quality of life and experience of care assessments] should be done maybe by either social worker or spiritual care coordinator, people that can sit down and talk about that, because I would feel that I’m not compassionate enough . . . Sometimes you ask one patient or one family member one question that requires a yes or no answer and they would go into like 10 minute conversation, which also pushes you back from what’s waiting for you out there, the bells are ringing and stuff. I cannot relax in knowing that my other people are having pain and I’m sitting here listening to a life story that has nothing to do with a patient. And I’ve learned, it’s actually a learned skill, to kind of interrupt the conversation and say, “Oh, so sorry. I just need to run.” But some of them are really hard. If somebody’s really upset, struggling with like husband or father of three young kids dying, then . . . I just rather not put myself into those shoes that I ask the question and then I can’t fulfill that, so I’d rather not even initiate that conversation, because I know if I do, and maybe it’s not the best practice, but I feel if I start, I need to finish, and I can’t, and then I feel really bad . . . [and] when I leave, I have to put that smile back on and go to next room and pretend that nothing happened in the next room.* |
| 6 | Schick-Makaroff and Molzahn, (2017) | HCP | The nurses’ lack of familiarity with the KDQOL-36 necessitated that  they spend more time with it, and sometimes that time wasn’t available.  *If I had two things to look at, I'm going to choose the one that I'm familiar with because it takes me more time to figure that [KDQOL-36] out.* |
| 7 | Snyder et al (2013) | HCP | *No, I didn’t log onto the website. It is too much trouble. I forgot that I could find the results in EPR.* |
| 8 | Tolstrup et al (2020) | HCP | HCPs reported that the inclusion of patient reporting was seen as being more time-consuming than a typical consultation due to the fact that the clinicians had to log into another system to see the report. |
| 9 | Baeksted et al (2017) | HCP | *It is a barrier that you have to log in to another system [AmbuFlex] if it does not substitute other tasks* |
| 10 | Grossman et al (2018) | HCP | Two providers reported that PROs might reduce their cognitive load  *Some patients, regardless of their literacy, are just not good at communicating what’s going on. They want to tell you a million different things. You have to be the interpreter and say: ‘From all these things you want to tell me, it really seems like everything has to do with fatigue.’ I think [mi. Symptoms] may help with this.* |
| 11 | Sandhu et al (2020) | HCP | *A patient might rank everything 10 out of 10, and if that’s the case, I don’t have time to address every symptom*  *We’re totally inundated with patient data that may or may not be meaningful, so having yet another electronic something that doesn’t actually enhance our interactions with patients for patient care—nobody wants that* |
| **FACILITATORS** | | | |
| 1 | Spaulding et al (2019) | HCPs and front desk staff | HCPs described the new process and ePSRM tool as faster, more efficient, and saved time |
| 2 | Spaulding et al (2019) | HCP | *It's cut our reports by about 55..We type our notes and it used to take me for a complicated patient sometimes up to 45 minutes or so to write that lengthy report. Now it takes me about 18 minutes … all of that time that is saved is then used to actually talk to the patient*  They found that the new ePSRM creates uniformity in how notes are generated  *In many ways, I look at this as a burn‐out prevention tool because interaction becomes more meaningful, it's a lot more enjoyable, and you finish on time*  *It's cut it down by 10%, 15%, 20% so that just gives you a little bit more breathing room. And time to be able to, respond back to other questions from patients that are calling you, or emailing you or whatever else, and so you get out a little earlier* |
| 3 | Spaulding et al (2019) | Front desk staff | *the providers are able to do what they need to do quicker and get the patient back, versus waiting on them to fill out the paper, going over it, and then calling the patient back* |
| 4 | Mou et al (2021) | HCP | *I love the fact that I'm not actually having to ask all these [PRO] questions… [I] would have to spend an hour and a half with someone if [I] were doing all these little questions one by one*  *[From a] data entry perspective [PROs are] really helpful… think about what it’s like to be a patient in a doctor’s office when your doctor is sitting there staring at the computer just asking you hundreds of questions*  *My favorite is the annual wellness visit because all the pertinent questions can get pre-populated into the notes. That’s incredibly helpful* |
| 5 | Schick-Makaroff and Molzahn, (2017) | HCP | Nurses believed that by focusing their assessments, they saved time and altered how they did their work. And as Nurse 8 commented, “has it changed your practice? “*I think it does.”* |
| 6 | Snyder et al (2013) | HCP | *It was less painful than I thought it would be.* |
| 7 | Yamada et al (2020) | Patients | *It would save a lot of time when you’re in seeing the doctor. All of the questions would be right there in front of them, make it a lot easier for the doctor to assess* |
| 8 | Baeksted et al (2017) | HCP | Oncologists found the AmbuFlex software easy to use and did not spend more time than usual on the consultation when using the software |
| 9 | Sandhu et al (2020) | HCP | *The next step in efficiency is to be able to screen before clinic starts and much more anticipate who’s going to need what and how quickly patients can go through the system. . . . You could then begin to use that to triage how you got people in the rooms, who you knew you could get through quickly*  Respondents described examples of facilitators such as creating ePRO documentation templates, automatic distribution to patients, and easy-to-remember dot phrases (eg, “.PRO”) that pull structured ePRO data from the EHR into their free text note. |

**Additional work with PRMs completion**

**Finding #27:** Older adults and healthcare professionals cited that older adults perceived PRMs completion was additional work and use of electronic systems to complete PRMs was time consuming, hindering older adults from electronically completing PRMs.

| **#** | **Reference** | **Perspective of:** | **Extracted data relevant to finding** |
| --- | --- | --- | --- |
| **BARRIERS** | | | |
| 1 | Snyder et al (2013) | Patients | *It really was just one more thing to do. I didn’t feel that good a lot of the time so I really didn’t feel like doing one more thing. But I did it because I had to.* |
| 2 | Yamada et al (2020) | Patients | Several participants mentioned that competing priorities might influence whether they would complete the questionnaire in advance of their medical appointment  *I have my two personal emails. I have 4 different work emails that I have to go through every day. Like it’s swamped with these things*  *You may forget to fill it out. I mean, with society pressure, you know. With everything, with work, with, you know just your family, with just everything and you being sick maybe most of the time, I can speak of myself, you know, I think I would forget that I have to fill it out* |
| 3 | Samuel et al (2020) | Patients | *It just didn’t seem like it was an advantage or a disadvantage. Just another form to fill out but it didn’t have much impact on my life, to be honest* |
| 4 | Duman-Lubberding et al (2017) | HCP | Using OncoQuest takes too much time according to their patients |

**Thematic category: ORGANISATIONAL FACTORS**

**Team culture and collaboration (*)**

**Finding #28:** Multi-disciplinary team communication, positive team culture and peer to peer support were cited as facilitators to electronic collection and use of PRMs by some healthcare professionals, while some reported that challenges in multi-disciplinary collaboration and different levels of motivation within the team to collect and use PRMs were barriers electronic collection and use of PRMs.

| **#** | **Reference** | **Perspective of:** | **Extracted data relevant to finding** |
| --- | --- | --- | --- |
| **BARRIERS** | | | |
| 1 | Amini et al (2021) | HCP | Challenges in multidisciplinary collaboration due to conflicting interests, schedules, and task division was reported as barrier  Different motivation levels within the disease team to use PROMs |
| 2 | Krawczyk et al (2019) | HCP | *Well, it’s always nice to have a resource, like to back up whatever you are saying you need to do. But we have been [doing this] a long time, so we know how the team works. [But] for me, it’s always nice to repeat . . .* |
| **FACILITATORS** | | | |
| 1 | Amini et al (2021) | HCP | HCPs discussed that motivation/enthusiasm/interest within the disease team is needed for successfully implementing PROMs  Collaboration with multiple disciplines on every level of care was discussed as an encouraging factor  The past experience of other colleagues was reported as a facilitator |
| 2 | Kaur et al (2019) | HCP | *I will tell you there’s one possibility is that you find out you’re not as good as you think you are. And you’re getting worse outcomes. I suppose we might prefer calling it the “ostrich approach.” I do not want to know, because maybe I am not as good as I think I am. Ideally, if we learned that we are not as good as we think we are, then we would respond by trying to improve our results. Until we have this [PROs in practice] like if I have no quality improvement analysis at all, I am not going to know how my outcomes are. So, I must admit I am working in a void right now. It would, of course, it would be useful.*  The surgeons recognised the value of routine collection of PROMs in clinical practice regarding benchmarking of outcomes against other plastic surgeons |
| 3 | Spaulding et al (2019) | HCP | HCPs discussed having others who were familiar with the program helped them with their transition  *Make sure that it is not going to slow the practice, for that was one of my concerns. How much time was I going to spend familiarizing myself with the whole process, but it went quite well*  Peer to peer teaching/learning was also reported as beneficial to the success of the intervention  *We, we got it very quickly because there were two of us, right across the hall and we could learn from each other* |
| 4 | Krawczyk et al (2019) | HCP | *Yeah, and even newer employee nurses on our unit wouldn’t need it for long.*  *And plus, the mentorship on the unit is pretty stellar in terms of new people coming to the unit. They’re never left to just kind of figure it out, right? And even if somebody doesn’t know how to do something, we often consult each other as to what to do next or, you know, “I tried this and this, but I can’t think of any other options. What do you do?”*  *And if we didn’t know, we would bring it up in rounds.* |
| 5 | Schick-Makaroff and Molzahn, (2017) | HCP | Nurses discussed how they highlighted patients’ priorities to their colleagues for interdisciplinary follow-up  *“I'll give a verbal report with the patient too because they're part of that discussion and things that we have highlighted.”*  *“because it's a team thing we need - everybody has to be on the same page so we can all help the patient work through the particular concerns that they're having.”* |

**Leadership and champions (*)**

**Finding #29:** Healthcare professionals reported that supportive leadership and having champions facilitated electronic collection and use of PRMs. They valued the mentorship they received and buy-in from their leadership.

| **#** | **Reference** | **Perspective of:** | **Extracted data relevant to finding** |
| --- | --- | --- | --- |
| **FACILITATORS** | | | |
| 1 | Amini et al (2021) | HCP | Presence of a champion for PROMs |
| 2 | Spaulding et al (2019) | HCP | HCPs in this study noted that having leadership buy in, support staff, and the right space to experiment with how the new tool worked, created an environment that made it easy for the technology to be adopted into practice  *The main thing was the buy‐in from people in leadership. I mean because none of these things can happen without that at various levels. And I think that the biggest advantage that we had was that we were able to create a safe space to experiment* |
| 3 | Krawczyk et al (2019) | HCP | *And plus, the mentorship on the unit is pretty stellar in terms of new people coming to the unit. They’re never left to just kind of figure it out, right? And even if somebody doesn’t know how to do something, we often consult each other as to what to do next or, you know, “I tried this and this, but I can’t think of any other options. What do you do?”* |

**Change management**

**Finding #30:** Some healthcare professionals cited that the flexibility provided to rapidly iterate the electronic system for better acceptance and having a clearly communicated operational process facilitate the electronic collection and use of PRMs. Lack of uniformity in the implementation process and lack of established operational workflows (e.g., training and accessing PRMs electronically) were reported as barriers to electronic collection and use of PRMs by some healthcare professionals.

Older adults and administrative staff believed training and technical support workflows (e.g., trouble shoot technical problems) facilitated the electronic collection of PRMs.

| **#** | **Reference** | **Perspective of:** | **Extracted data relevant to finding** |
| --- | --- | --- | --- |
| **BARRIERS** | | | |
| 1 | Amini et al (2021) | HCP | Change management workflows:  Lack of uniformity in implementation process  Excessive duration of the implementation process |
| 2 | Spaulding et al (2019) | HCP | Establishing operations workflows:  There were concerns of adoption of tool into practise within the department and how the implementation might change the workflow  *If it's going to make life more difficult, it's not going to happen. So that's the key thing* |
| 3 | Mou et al (2021) | HCP | Training workflows:  *When [PROs] started there was not a great… education of the primary care community in terms of what they were going to be and the best workflow… to use them. The worst thing is occasionally I get a message in my [EHR] in-basket that one of my patients answered, [indicating] that they wanted to kill themselves. I don't believe primary care has been trained enough in terms of what to do with those answers.* |
| 4 | Snyder et al (2013) | HCP | Establishing operations workflows:  *No, I didn’t log onto the website. It is too much trouble. I forgot that I could find the results in EPR. Indicates no established operational workflow* |
| 5 | Baeksted et al (2017) | HCP | Training workflows:  One oncologist reported being given the introduction to the system  after he had started using it, indicating lack of operational processes for good training programs for new staff:  *I came to the department after the project had started. I had some questions about how to use the system* |
| 6 | Sandhu et al (2020) | HCP | Establishing operational workflows:  Oncologists reported uncertainty about which clinicians are responsible for viewing the ePRO results in multidisciplinary clinics, when patients might see a radiation oncologist, medical oncologist, and surgical oncologist during the same day |
| **FACILITATORS** | | | |
| 1 | Spaulding et al (2019) | HCP | Flexibility in electronic system development:  Participants were quick to acknowledge the flexibility in the implementation was helpful in the adoption of this implementation.  *The other thing was the inherent flexibility … we could go through various iterations very quickly and that allowed us to get to a point where we were not afraid of experimenting* |
| 2 | Spaulding et al (2019) | Front desk staff | Training workflows:  One continued maintenance aspect expressed by multiple participants focused on continued training.  *Consistent training on it so that we [are] always kept up to speed on what we have to do. Training new staff if they come in would maintain consistency across the board I believe* |
| 3 | Spaulding et al (2019) | HCP | Establishing operational workflows:  Additionally, organisational mapping to enhance the workflow and the process for the use of the iPad or having a system that maps how ePSRM information is sent to all physicians is important.  *Or some process where as soon as the nurse finishes her part, it can be printed out or something and left for the provider … one thing we need to push going forward is to figure out that piece* |
| 4 | Tolstrup et al (2020) | Patients | Technical support workflows:  Patients reported the possibility to contact the department in case of technical problems encountered with reporting PRMs electronically |
| 5 | Samuel et al (2020) | Patients | Training workflows:  Participants described positive experiences using the ePRO survey, including satisfaction with the training processes |

**Resources to manage collection and use of PRMs**

**Finding #31:** Some older adults reported that receiving adequate technical support and education would facilitate completion of PRMs electronically. The technical support available to resolve any technical problems was cited as a facilitator to electronic completion of PRMs by some older adults

Healthcare professionals reported that adequate education, support staff, support in the form of reminders during consultation hours prompting them to review PRMs responses, digital technology infrastructure that support seamless data integration, and government funding to cover digital technology infrastructure costs were facilitators to the electronic collection and use of PRMs. Barriers to electronic collection and use of PRMs cited by healthcare professionals were lack of digital technology infrastructure to support integration of PRMs in EHRs or other platforms clinicians use, education (e.g., to use electronic systems and interpret PRMs responses), high costs and lack of support staff. Healthcare professionals believed adequate education, digital technology infrastructure that allowed for integration of PRMs with EHRs, funding and support staff facilitated electronic collection and use of PRMs. A few healthcare professionals reported that receiving a guideline for interpretation of PRMs responses facilitated electronic collection and use of PRMs.

Administrative staff particularly felt that access to interpreter services, technical support staff and adequate education facilitated electronic collection of PRMs.

| **#** | **Reference** | **Perspective of:** | **Extracted data relevant to finding** |
| --- | --- | --- | --- |
| **BARRIERS** | | | |
| 1 | Amini et al (2021) | HCP | IT infrastructure:  Issues with IT infrastructure related to integration of PROMs into the EHR and visualisation of dashboard discussed  Insufficient staff for support and insufficient staff for coordination were reported as barriers |
| 2 | Kaur et al (2019) | HCP | IT infrastructure and funding:  TickiT could not export data at the time of the study and has upfront and ongoing costs associated with the software (ie, e-PROM customization costs, subscription costs)  *We are past the stage of high engagement with the electronic stuff that younger people will have. But I am sort of also looking at there’s extra time on me, there’s* ***extra money*** *and at the end, have we improved outcomes? I know there’s a noble drive for it, for any given patient to give them the best outcome they can have. That sort of more along an ethical call for us. But in practice, that’s against the realities of trying to churn people through your office, because there’s a lot of work to be done.* |
| 3 | Mou et al (2021) | HCP | Education:  *When [PROs] started there was not a great… education of the primary care community in terms of what they were going to be and the best workflow… to use them. The worst thing is occasionally I get a message in my [EHR] in-basket that one of my patients answered, [indicating] that they wanted to kill themselves… I don't believe primary care has been trained enough in terms of what to do with those answers.*  *I'd love to see… somebody [sitting] elbow to elbow with [PCPs] and watched [to see] what their workflow is to figure out how to how to make [PROs work].* |
| 4 | Krawczyk et al (2019) | HCP | Support staff:  HCPs cited increasing workloads, a lack of resources for efficiently sharing results and addressing patient and family member concerns once identified, discomfort in dealing with emotional distress, and/or belief that these issues are primarily the professional domains of social work and/or spiritual care.  *I think it [quality of life and experience of care assessments] should be done maybe by either social worker or spiritual care coordinator, people that can sit down and talk about that, because I would feel that I’m not compassionate enough . . . Sometimes you ask one patient or one family member one question that requires a yes or no answer and they would go into like 10 minute conversation, which also pushes you back from what’s waiting for you out there, the bells are ringing and stuff. I cannot relax in knowing that my other people are having pain and I’m sitting here listening to a life story that has nothing to do with a patient. And I’ve learned, it’s actually a learned skill, to kind of interrupt the conversation and say, “Oh, so sorry. I just need to run.” But some of them are really hard. If somebody’s really upset, struggling with like husband or father of three young kids dying, then . . . I just rather not put myself into those shoes that I ask the question and then I can’t fulfill that, so I’d rather not even initiate that conversation, because I know if I do, and maybe it’s not the best practice, but I feel if I start, I need to finish, and I can’t, and then I feel really bad . . . [and] when I leave, I have to put that smile back on and go to next room and pretend that nothing happened in the next room.* |
| 5 | Baeksted et al (2017) | HCP | Education:  One oncologist reported being given the introduction to the system  after he had started using it:  *I came to the department after the project had started. I had some questions about how to use the system*  IT infrastructure:  One oncologist reported the need to log into another system indicating lack of seamless integration of systems  *It is a barrier that you have to log in to another system [AmbuFlex] if it does not substitute other tasks.* |
| 6 | Duman-Lubberding et al (2017) | HCP | IT infrastructure:  The availability of OncoQuest outcomes during consultation is hampered due to difficulties to trace OncoQuest results at their computer screen |
| **FACILITATORS** | | | |
| 1 | Amini et al (2021) | HCP | Support staff:  HCPs discussed sufficient support from the central VBHC team promotes the implementation  IT infrastructure:  HCPs reported well-organized IT as a facilitating factor  Education:  The provision of a guideline for interpreting PROMs, team building around one clinical condition, sufficient resources, and the presence of a nurse practitioner were discussed as facilitators  Guideline for PROM interpretation reported as facilitator |
| 2 | Kaur et al (2019) | HCP | Support staff:  For successful implementation of a routine collection of PROMs, the surgeons identified a need for organizational change consisting of additional staff hours, role-specific training for clinic staff (administrative, nursing, residents/fellows), revised clinic logistics, and infrastructure.  Funding:  Financial support extended to cover the cost of the e-PROM platform by the Ministry of Health was proposed as a facilitator. |
| 3 | Spaulding et al (2019) | Front desk staff (Adm) | Support staff (technical):  One of the major factors that enhanced the implementation of the new ePSRM tool was having a technical support person available at all times  *We have great help if we don't know what to do or how to do it. Someone's there to help us, so we know where to, who to reach out to*  Education:  One continued maintenance aspect expressed by multiple participants focused on continued training  *Consistent training on it so that we [are] always kept up to speed on what we have to do. Training new staff if they come in would maintain consistency across the board I believe* |
| 4 | Navarro-Millán et al (2019) | Patients | Education:  *We show you, you know, how to access it through your email,” or we take your phone and say, “This is how you find this app. This is how you do it.” Have someone, whether it’s a receptionist, or a nurse, or somebody from the IT department, say, “Okay, this is the person who’s going to help the people who aren’t tech savvy access this stuff.*  Support staff (technical):  *I’m all about apps and stuff like that. But for people who aren’t, have someone in the office to show them, walk them through it step by step and make sure that they’re okay with it before they leave.*  Education and Support staff (technical):  Patients that expressed difficulty with technology indicated that having formal instruction or someone to assist or engage them in the electronic communication could empower them to consider this avenue |
| 5 | Snyder et al (2013) | HCP | Education:  Clinicians wanted more explanation about the PRO item content and score meaning |
| 6 | Tolstrup et al (2020) | HCP | IT infrastructure:  Having the reports integrated in the electronic health records (EHRs) was stated not only to save time but also make it much easier to remember to include them in the consultation |
| 7 | Tolstrup et al (2020) | Patients | Support staff (technical):  There were only minor technical challenges, and the patients were very compliant and contacted the department in case of any technical problems. |
| 8 | Yamada et al (2020) | Patients | Support staff (technical):  *I know that there’s no time in the doctor’s office to put you through it but I do think it would help people that aren’t computer savvy to have someone run through it with them once* |
| 9 | Duman-Lubberding et al (2017) | HCP | IT infrastructure:  Provision of feedback to the physician within the electronic patient file (instead of via a standalone application) |
| 10 | Moradian et al (2018) | Patients | Support staff (technical):  As participants were not familiar with the system prior to the usability session and no tutorial of the ASyMS and its functionalities was given, they often felt insecure about their actions and asked for assistance and approval before performing tasks. |

**Thematic category: PRMs QUESTIONNAIRE SELECTION AND DESIGN**

**Questionnaire length and complexity of questions**

**Finding #32****:** Some older adults cited that having simple, easy-to-understand questions facilitated electronic completion of PRMs. Some older adults and healthcare professionals cited that lengthy questionnaires containing hard to comprehend questions and those that took a long period of time to complete were barriers to electronic completion of PRMs.

Some healthcare professionals believed that questions should be asked in a way that would highlight the patient’s problems and that the overlapping number of questions asked repeatedly during each clinic visit may be reduced by sharing PRMs responses between clinicians.

| **#** | **Reference** | **Perspective of:** | **Extracted data relevant to finding** |
| --- | --- | --- | --- |
| **BARRIERS** | | | |
| 1 | Spaulding et al (2019) | HCP | HCPs discussed patient’s inability to comprehend the questions and content of the questionnaire |
| 2 | Mou et al (2021) | HCP | *[The PRO] questions should be formatted and asked in a way so that at a glance you can see [the] problems.*  *[survey] takes too long, [patients] don’t like to have to repeat at every visit* |
| 3 | Brochmann et al (2016) | Patients | One participant rated the number of questions as *‘‘many’”*  *It is important to answer the questions, I think. Some questions seem alike* |
| 4 | Baeksted et al (2017) | HCP | One oncologist stated that patients could find it difficult to separate the  gradings ‘severity’ and ‘influence on daily activity’  *Too many similar questions. But the right thing to do—for both patients and doctors.* |
| 5 | Samuel et al (2020) | Patients | *When I first started out, I didn’t understand some of the questions. Then I asked the nurse and she told me what to do. Then I was good to go* |
| 6 | Nielsen et al (2021) | Patients | Participants in this study found it difficult to rate their own pain levels, particularly over time  *I do not think it is easy… but I do answer of course. Everything is… It does not hurt as much so you’re not able to walk…. And then you think… how do you modulate that… I’m able to go to work, I’m able to walk… It is not like that it affects me in a way where I’m not able to work. I imagine others feeling that way… so if I have to modulate it, then my score is rather low. Also compared to how you can feel…. When I was sick.* |
| 7 | Grossman et al (2018) | Patients | Patients reported trouble understanding PRO surveys - difficulty comprehending the PRO survey questions and answer choices  *The way the questions were worded was not straightforward. Could the questions just be more clear* |
| 8 | Sandhu et al (2020) | HCP | *Some of the terms like fatigue versus tired, fatigue versus dyspnea, shortness of breath can be difficult for patients to discern between. So, if you walked up four flights of stairs and at the end are you tired or short of breath? Some people are both* |
| **FACILITATORS** | | | |
| 1 | Aiyegbusi et al (2018) | Patients | *Clear and easy to understand. It didn't appear to have any trick questions*  *The questions were straightforward* |
| 2 | Schick-Makaroff and Molzahn, (2017) | HCP | The nurses also pointed out that there was a lot of “overlapping” between the mandated ESAS-r:Renal, their nursing assessment checklists, and unit charting documents. Nurse participants were hopeful that when ePRO results were shared between clinicians, then they could “reduce the number of questions” that were repeatedly asked to each patients during  each clinic visit |
| 3 | Brochmann et al (2016) | Patients | Participants assessed the total amount of questions in the questionnaires as acceptable  *I think it is just the right number of questions* |
| 4 | Tolstrup et al (2020) | Patients | The patients reported that the number of items and the length of the questionnaire were appropriate and that reporting on a weekly basis was fitting |
| 5 | Yamada et al (2020) | Patients | Patients reported the questionnaire was easy, straight forward and concise to complete  *It was easy. It was pretty straight-forward. I could understand everything* |

**Questions relevant to patient’s health**

**Finding #33:** Some older adults and healthcare professionals reported that having questions specific to the patient’s health (e.g., behavioural activation, mental health and sexual function) facilitated electronic collection and use of PRMs. Some older adults and healthcare professionals cited that the lack of specific questions addressing patient’s health (e.g., co-morbid conditions and specific disease) hindered electronic collection and use of PRMs.

| **#** | **Reference** | **Perspective of:** | **Extracted data relevant to finding** |
| --- | --- | --- | --- |
| **BARRIERS** | | | |
| 1 | Snyder et al (2013) | HCP | *Until you address the scaling issues it isn’t very useful…think about the subscales and whether or not they are really relevant. Perhaps use different measures depending upon the state of the disease for that patient.* |
| 2 | Samuel et al (2020) | Patients | Participants suggested additional health or symptom-related items to include in the ePRO system (e.g., about comorbid conditions)  *I’m being treated for prostate cancer, but I also have diabetes…. The two are really hard sometimes to take care of both at one time. Somebody [on] the survey [study team] needs to realize that people are being treated for multiple situations* |
| 3 | Grossman et al (2018) | Patients | *If you have a bunch of things that you can’t really keep track of, they won’t come up until you’re asked the specific [mi.Symptoms] questions. If you’re not asked the specific questions, you’re not going to remember all the symptoms that you’re having* |
| 4 | Sandhu et al (2020) | HCP | *I’m not that worried about their global score. I want to know the specific problems that they’re having that we need to address. . . . What would be more helpful for me is something that is specific for lung cancer*  *Give you a global sense of how [patients] are doing and—for what I [a radiation oncologist] do—there’s not a lot of actionable items. I think it might be very different for [a medical oncologist] who’s giving chemo. We approach our field a little bit more like in a proceduralist type of way where we focus on a discrete problem. So, for me, if it’s not addressing that problem, it’s ancillary data* |
| **FACILITATORS** | | | |
| 1 | Spaulding et al (2019) | HCP | Two physicians emphasized that in the past, they asked patients questions on behavioural activation and had to use their own judgement about physical activity, but they do not have to do that any longer with the validated tools to assess physical activity. In fact, these validated tools ensure screening is formalized and consistent |
| 2 | Mou et al (2021) | HCP | *[include] detailed smoking history, sexual activity, menstrual history, family history*  *Family history should be outsourced to the patients*  *[include] Sleep Exercise/activity habits Commuting time Caffeine intake* |
| 3 | Snyder et al (2013) | Patients | Patients recommended improving the intervention to include tailoring questions to be applicable to the individual |
| 4 | Nielsen et al (2021) | Patients | Some of the patients who used the digital PRO system for follow-up and replacement of consultations also found beneficial the inclusion of questions on issues like mental health, fatigue and sexual function  *many of the consultations you encounter are based on blood samples, and then you just talk about that and not so much about me, who is sitting over here. Then it can be… then these questions come… but ok, they actually do ask this in these questionnaires. But it will never be sufficient to see the entire me. Right?* |
| 5 | Grossman et al (2018) | HCP | Two providers felt mi.Symptoms might identify missed opportunities for medical intervention, by prompting patients to thoroughly report symptoms. One provider described how mi.Symptoms might enhance symptom review  *[Some heart failure patients] get this upper gastrointestinal gas buildup. A lot of them have complained to me: ‘Nobody ever asked me about that. Nobody ever does anything about it.’ That’s something I would definitely ask on [mi.Symptoms]* |

**PRMs response capture option**

**Finding #34:** Some older adults found a free-text field to add comments to qualify the numerical rating of their health facilitated electronic completion of PRMs. The lack of a free text field to capture patient’s health was cited as a barrier by some older adults and healthcare professionals. Some older adults reported they could not use the free text field due to difficulties in information articulation or if they experienced dyslexia, hindering electronic completion of PRMs.

Most healthcare professionals found the numerical rating of patient health as more useful, while some healthcare professionals found free text description of patient health as more useful.

| **#** | **Reference** | **Perspective of:** | **Extracted data relevant to finding** |
| --- | --- | --- | --- |
| **BARRIERS** | | | |
| 1 | Mou et al (2021) | HCP | *all black and white answers and patients can't explain or qualify answers*  *Yes/No binary answer doesn't give them enough subtly to express concerns* |
| 2 | Brochmann et al (2016) | Patients | There was agreement that it would have been appropriate to have the opportunity to write a free text for healthcare professionals if the questionnaires did not sufficiently cover current symptoms and health difficulties  *I miss a box, where I can write symptoms, you do not ask for in the questionnaires* |
| 3 | Nielsen et al (2021) | Patients | The PRO system included a free text field, which challenged patients who found it **difficult to articulate information about their health in writing, reflecting a deficit in understanding of health concepts and language**  *I would prefer talking to a doctor. Most likely. Because there is always something, some questions, some thoughts, that, when you sit in front in a consultation, will appear. I think it is like that, if you have a comment or something in the end of the questionnaire, you do not always get it written, and how are you supposed to write something like that. It is difficult to express feelings. It is easier to look someone in the eye.*  Two participants reported they were **dyslexic**, which meant that they found it more difficult to use a free text field  *I’m not good at… I’m dyslexic, I’m not good at formulating thing in writing. I just like to check boxes, and that’s really fine, that you are supposed to do*  *that in the questionnaire*  Most of the other participants also reported that **they did not use the free text field often** |
| 4 | Grossman et al (2018) | Patients | Patients reported lack of unstructured communication - Participants wanted to use messaging to ask their physician questions or add comments to their survey results  *At the end, if you want make [a] comment you should be able to add one.* |
| **FACILITATORS** | | | |
| 1 | Krawczyk et al (2019) | HCP | *It’s [quality of life or experiences of care] being turned into a numerical digit that . . . that allows us to track in a potentially more accurate way . . . but sometimes [results] can be misleading as well. But I think that’s where it’s useful is over time because we know [a patient] might be feeling very distressed because she just had an argument with her husband [an hour] ago, and that*  *can be an anomaly, seeing it over a week, that a certain thing is poor over a whole week, then that gives us the [bigger picture]* |
| 2 | Schick-Makaroff and Molzahn, (2017) | HCP | The nurses thought that the numeric scores offered “safety” to the patient to express concerns in numbers and not words.  *“It's easier for them (pause) on a scale like than to say, ‘like this is really bothering me’ because sometimes when they use descriptive words it doesn't sound as (pause) bothersome as when you put in a number.”* |
| 3 | Snyder et al (2013) | HCP | *I found the free text areas most helpful. That got my attention more*  *than the numbers did.* |
| 4 | Tolstrup et al (2020) | Patients | A few of the patients would have liked a free text field where they could write a comment or elaborate if the questionnaire did not adequately cover existing symptoms  *It is as if you (health care professionals) don’t get enough information* |
