# Supplementary material for: Barriers and facilitators to the uptake of electronic collection and use of patient-reported measures in routine care of older adults: a systematic review with qualitative evidence synthesis
Source: JAMIA Open. 2024 Aug 2;7(3):ooae068. doi: 10.1093/jamiaopen/ooae068 (PMC11296862; doi:10.1093/jamiaopen/ooae068)
Supplement: ooae068_Supplementary_Data [file ooae068_supplementary_data.zip › ooae068_Supplementary_Data/Appendix 7_Confidence assessment_refs revised.docx]

| Supplemental appendix 7 – Confidence assessment of findings | | | | | | | |
| --- | --- | --- | --- | --- | --- | --- | --- |
| # | **Summary of finding** | **Studies contributing to finding** | **Methodological limitations** | **Relevance** | **Coherence** | **Adequacy** | **CERQual assessment - confidence in the evidence** |
| Thematic category: Older adults’ characteristics | | | | | | | |
| *Clinical conditions and socio-cultural factors* | | | | | | | |
| 1 | Older adults with physical disability, visual and cognitive impairment, and communication difficulties (e.g., PRMs administered in non-native language) reported that electronic completion of PRMs could be challenging.  Caregivers perceived that older adults experienced problems in concentrating for a long period of time when completing PRMs.  Healthcare professionals cited that older adults completing PRMs in a non-native language and those with cognitive disabilities experienced difficulty in comprehending questions, while visually impaired older adults found switching between multiple screens on an electronic device difficult.  Administrative staff also cited that older adults completing PRMs administered in a non-native language were less likely to understand questions | [10, 33, 34, 36, 38, 40, 42, 46, 48, 51, 52] | 11 studies varied in methodological limitations  Strengths included clear aims (n = 11), appropriate methods (n = 11), ethics (n = 11) and clear statement of findings (n = 11)  Weaknesses related to justification of design (no: n = 2), recruitment strategy (no: n = 2), data collection (no: n = 1), discussing relationship between researcher and participant (no: n = 6) and data analysis (no: n = 4)  8 of the 11 studies have minor or no methodological limitations  *Minor concerns* | Majority of studies conducted recently and contribute relevant data  Majority of studies conducted in hospital outpatient setting. Limited coverage of other healthcare settings and multi-disciplinary care.  Limited information provided on proportion of older adults above 65 years involved in study or being treated. 5 studies indicate mean or median age of patients close to or above 65 years  *Moderate concerns* | Good fit between data and finding  *No or very minor concerns* | Data from 11 of 22 studies contributed to this finding.  Studies cover views of older adults (n = 8), caregivers (n = 1), administrative staff (n = 1) and healthcare professionals (n = 3).  Minor concerns about richness of data in some studies (n = 6)  *Moderate concerns* | **Moderate confidence**  Data was coherent. Minor concerns about methodological limitation. Moderate concerns about relevance and adequacy |
| *Access to digital technology* | | | | | | | |
| 2 | As cited by some older adults and healthcare professionals, the lack of access to necessary digital technology (e.g., electronic devices and e-mail) hindered older adults’ capability to electronically complete PRMs. Similarly, older adults and healthcare professionals cited that having access to necessary digital technology (e.g., electronic devices and internet) facilitated older adults’ completion of PRMs electronically | [10, 35, 40, 50, 53] | 5 studies varied in methodological limitations  Strengths included clear aims (n = 5), appropriate methods (n = 5) and ethics (n = 5)  Weaknesses related to justification of design (no: n = 1), recruitment strategy (no: n = 2), data collection (no: n = 2), discussing relationship between researcher and participant (no: n = 3), data analysis (no: n = 1) and clear statement of findings (no: n = 1)    3 of the 5 studies have minor or no methodological limitations  *Minor concerns* | Majority of studies conducted recently and contribute relevant data  All studies conducted in hospital outpatient setting. Limited coverage of other healthcare settings and multi-disciplinary care.  Limited information provided on proportion of older adults above 65 years involved in study or being treated. No studies indicate mean or median age of patients close to or above 65 years  *Moderate concerns* | Good fit between data and finding  *No or very minor concerns* | Data from 5 of 22 studies contributed to this finding.  Studies cover views of older adults (n = 3) and healthcare professionals (n = 2).  Minor concerns about richness of data in some studies (n = 4)  *Moderate concerns* | **Moderate confidence**  Data was coherent. Minor concerns about methodological limitation. Moderate concerns about relevance and adequacy |
| Thematic category: Digital Technology | | | | | | | |
| *User interface for older adults* | | | | | | | |
| 3 | Older adults reported that having an easy-to-use, intuitive interface with the following features facilitated electronic completion of PRMs:   - large font and screen size - visually appealing with colour, images and graphs - clear labelling of response options and data - indication of questionnaire progress (e.g., progress bar) - ability to download or bookmark link to PROMs software on various electronic devices - multiple questions per page   Healthcare professionals believed that an easy-to-use and visually appealing interface enables older adults to complete PRMs electronically.  Older adults not having to view all the questions at once when completing PRMs electronically was cited as a facilitator by healthcare professionals and administrative staff.  Some older adults and healthcare professionals believed that a touchscreen option facilitated older adults’ completion of PRMs electronically | [10, 33, 35, 36, 37, 43, 44, 45, 47, 49, 52] | 11 studies varied in methodological limitations  Strengths included clear aims (n = 11), appropriate methods (n = 11) and ethics (n = 11)  Weaknesses related to justification of design (no: n = 1), recruitment strategy (no: n = 3), data collection (no: n = 2), discussing relationship between researcher and participant (no: n = 6), data analysis (no: n = 3) and clear statement of findings (no: n = 3)    8 of the 11 studies have minor or no methodological limitations  *Minor concerns* | Majority of studies conducted recently and contribute relevant data  Majority of studies conducted in hospital outpatient setting. Limited coverage of other healthcare settings and multi-disciplinary care.  Limited information provided on proportion of older adults above 65 years involved in study or being treated. 5 studies indicate mean or median age of patients close to or above 65 years  *Moderate concerns* | Good fit between data and finding  *No or very minor concerns* | Data from 11 of 22 studies contributed to this finding.  Studies cover views of older adults (n = 10), administrative staff (n = 1) and healthcare professionals (n = 2).  Minor concerns about richness of data in some studies (n = 6)  *Minor concerns* | **Moderate confidence**  Data was coherent. Minor concerns about methodological limitation. Moderate concerns about relevance and minor concerns about adequacy |
| 4 | Older adults reported that the following interface features hindered their ability to complete PRMs electronically:   - small font and screen size - many scrolls to navigate through questionnaire - use of certain display graphics (e.g., use of multiple colours, checkered boxes, circle options and smiley faces) - unresponsive option and progress buttons - unlabelled response options - unnecessary pop-up of alerts - mobile application downloading features - drop downs were cumbersome   Some healthcare professionals cited that it was stressful for visually impaired older adults to click through multiple screens to complete PRMs electronically.  Caregivers and older adults experienced frustration when they could not progress through the questionnaire. Some healthcare professionals also cited that older adults experienced frustration with challenges in questionnaire progression.  Some older adults had difficulty using a touchscreen in absence of a touchscreen pen, due to the need to firmly press or for long periods with their fingers | [10, 33, 35, 36, 37, 38, 44, 45, 47, 52] | 10 studies varied in methodological limitations  Strengths included clear aims (n = 10), appropriate methods (n = 10) and ethics (n = 10)  Weaknesses related to justification of design (no: n = 1), recruitment strategy (no: n = 4), data collection (no: n = 2), discussing relationship between researcher and participant (no: n = 6), data analysis (no: n = 1) and clear statement of findings (no: n = 1)    8 of the 10 studies have minor or no methodological limitations  *Minor concerns* | Majority of studies conducted recently and contribute relevant data  Majority of studies conducted in hospital outpatient setting. Limited coverage of other healthcare settings and multi-disciplinary care.  Limited information provided on proportion of older adults above 65 years involved in study or being treated. 5 studies indicate mean or median age of patients close to or above 65 years  *Moderate concerns* | Good fit between data and finding  *No or very minor concerns* | Data from 10 of 22 studies contributed to this finding.  Studies cover views of older adults (n = 8), caregivers (n = 1), and healthcare professionals (n = 2).  Minor concerns about richness of data in some studies (n = 5)  *Minor concerns* | **Moderate confidence**  Data was coherent. Minor concerns about methodological limitation. Moderate concerns about relevance and minor concerns about adequacy |
| *User interface for healthcare professionals* | | | | | | | |
| 5 | Some healthcare professionals valued an easy-to-use interface that helped them access questionnaires on various devices and browsers, with a visually appealing layout that used colour to draw attention to areas of concern and/or changes over time, to engage in the electronic collection and use of PRMs. Some perceived that difficulties in accessing the electronic system, lack of clear labelling of questions, the need for many clicks to access key information and limited visual presentation of data were barriers to electronic collection and use of PRMs | [34, 35, 36, 38, 41, 43, 46, 53] | 8 studies varied in methodological limitations  Strengths included clear aims (n = 8), appropriate methods (n = 8), justification of design (n = 8) and ethics (n = 8)  Weaknesses related to recruitment strategy (no: n = 3), data collection (no: n = 1), discussing relationship between researcher and participant (no: n = 5), data analysis (no: n = 3) and clear statement of findings (no: n = 2)    5 of the 8 studies have minor or no methodological limitations  *Minor concerns* | Majority of studies conducted recently and contribute relevant data  All studies conducted in hospital outpatient setting. Limited coverage of other healthcare settings and multi-disciplinary care.  Limited information provided on proportion of older adults being treated. 2 studies indicate mean or median age of patients close to or above 65 years  *Minor concerns* | Good fit between data and finding  *No or very minor concerns* | Data from 8 of 22 studies contributed to this finding.  Studies cover views of healthcare professionals (n = 8).  Minor concerns about richness of data in some studies (n = 2)  *Minor concerns* | **High confidence**  Data was coherent. Minor concerns about methodological limitation, relevance and adequacy |
| *Electronic device for PRMs completion* | | | | | | | |
| 6 | Older adults generally were comfortable completing PRMs on portable electronic devices such as a laptop, tablet or smartphone, or on a desktop computer. Some older adults preferred using smartphones due to its screen colours and convenience to use the device, while some preferred a computer or tablet due to the larger screen size and reduced need for scrolling. A few older adults experienced difficulty in using a smartphone due to the side-to-side scrolls required. | [10, 33, 35, 44, 45, 46, 48, 52] | 8 studies varied in methodological limitations  Strengths included clear aims (n = 8), appropriate methods (n = 8) and ethics (n = 8)  Weaknesses related to justification of design (no: n = 1), recruitment strategy (no: n = 1), data collection (no: n = 2), discussing relationship between researcher and participant (no: n = 2), data analysis (no: n = 2) and clear statement of findings (no: n = 1)    6 of the 8 studies have minor or no methodological limitations  *Minor concerns* | Majority of studies conducted recently and contribute relevant data  All studies conducted in hospital outpatient setting. Limited coverage of other healthcare settings and multi-disciplinary care.  Limited information provided on proportion of older adults above 65 years involved in study or being treated. 5 studies indicate mean or median age of patients close to or above 65 years  *Moderate concerns* | Good fit between data and finding  *No or very minor concerns* | Data from 8 of 22 studies contributed to this finding.  Studies cover views of older adults (n = 8)  Minor concerns about richness of data in some studies (n = 2)  *Minor concerns* | **Moderate confidence**  Data was coherent. Minor concerns about methodological limitation. Moderate concerns about relevance and minor concerns about adequacy |
| *Technical challenges* | | | | | | | |
| 7 | Most older adults and healthcare professionals reported that technical challenges such as connectivity problems, device outages (e.g., going dark, logging out or system crashes), slow response time and device incompatibilities were barriers to electronic completion and use of PRMs. The support available to resolve any technical problems was cited as a facilitator to electronic completion of PRMs by some older adults | [10, 34, 35, 36, 37, 38, 46, 47] | 8 studies varied in methodological limitations  Strengths included clear aims (n = 8), appropriate methods (n = 8), justification of design (n = 8) and ethics (n = 8)  Weaknesses related to recruitment strategy (no: n = 5), data collection (no: n = 1), discussing relationship between researcher and participant (no: n = 6), data analysis (no: n = 2) and clear statement of findings (no: n = 1)    6 of the 8 studies have minor or no methodological limitations  *Minor concerns* | Majority of studies conducted recently and contribute relevant data  Majority of studies conducted in hospital outpatient setting. Limited coverage of other healthcare settings and multi-disciplinary care.  Limited information provided on proportion of older adults above 65 years involved in study or being treated. 3 studies indicate mean or median age of patients close to or above 65 years  *Moderate concerns* | Good fit between data and finding  *No or very minor concerns* | Data from 8 of 22 studies contributed to this finding.  Studies cover views of older adults (n = 5) and healthcare professionals (n = 4)  Minor concerns about richness of data in some studies (n = 3)  *Minor concerns* | **Moderate confidence**  Data was coherent. Minor concerns about methodological limitation. Moderate concerns about relevance and minor concerns about adequacy |
| *Privacy and security of personal data* | | | | | | | |
| 8 | Not knowing who could access personal and health data and what the data is used for, and exposure of data to malware viruses and hackers were cited as barriers to electronic completion of PRMs by most older adults. However, some older adults had no concerns about privacy risks when using their own device.  Healthcare professionals were concerned about data privacy and governance (e.g., who owns the data) when using certain software programs for patient data collection | [35, 45] | 2 studies varied in methodological limitations  Strengths included clear aims (n = 2), appropriate methods (n = 2), justification of design (n = 2), ethics (n = 2) and data analysis (n = 2)  Weaknesses related to recruitment strategy (no: n = 1), data collection (no: n = 1), discussing relationship between researcher and participant (no: n = 1) and clear statement of findings (no: n = 1)    1 of the 2 studies have no methodological limitations  *Moderate concerns* | All studies conducted recently and contribute relevant data  All studies conducted in hospital outpatient setting. Limited coverage of other healthcare settings and multi-disciplinary care.  Limited information provided on proportion of older adults above 65 years involved in study or being treated. No studies indicate mean or median age of patients close to or above 65 years  *Moderate concerns* | Good fit between data and finding  *No or very minor concerns* | Data from 2 of 22 studies contributed to this finding.  Studies cover views of older adults (n = 2) and healthcare professionals (n = 1)  Moderate concerns about richness of data in studies (n = 2)  *Moderate concerns* | **Low confidence**  Data was coherent. Moderate concerns about methodological limitation. Moderate concerns about relevance and adequacy |
| Thematic category: Support from social circle | | | | | | | |
| 9 | Older adults cited that support from family, caregivers, peers, healthcare professionals and healthcare services received in the form of help to understand and complete PRMs, support to use digital technology to complete PRMs, encouragement and reminders facilitated electronic completion of PRMs.  Healthcare professionals believed support from family and peers, while administrative staff believed helping patients (e.g., translation services for patients with language barriers) during completion of PRMs facilitated older adults’ completion of PRMs electronically.  One caregiver reported helping the older adult complete PROMs due to the cognitive challenges experienced by the older adult. | [10, 35, 36, 40, 41, 42, 44, 45, 47, 49] | 10 studies varied in methodological limitations  Strengths included clear aims (n = 10), appropriate methods (n = 10) and ethics (n = 10)  Weaknesses related to justification of design (no: n = 1), recruitment strategy (no: n = 2), data collection (no: n = 1), discussing relationship between researcher and participant (no: n = 5), data analysis (no: n = 2) and clear statement of findings (no: n = 2)    8 of the 10 studies have minor or no methodological limitations  *Minor concerns* | Majority of studies conducted recently and contribute relevant data  Majority of studies conducted in hospital outpatient setting. Limited coverage of other healthcare settings and multi-disciplinary care.  Limited information provided on proportion of older adults above 65 years involved in study or being treated. 4 studies indicate mean or median age of patients close to or above 65 years  *Moderate concerns* | Good fit between data and finding  *No or very minor concerns* | Data from 10 of 22 studies contributed to this finding.  Studies cover views of older adults (n = 8), administrative staff (n = 3), healthcare professionals (n = 4) and caregivers (n = 1)  Minor concerns about richness of data in some studies (n = 4)  *Minor concerns* | **Moderate confidence**  Data was coherent. Minor concerns about methodological limitation and adequacy. Moderate concerns about relevance |
| Thematic category: Knowledge and skills | | | | | | | |
| *Digital knowledge and skills* | | | | | | | |
| 10 | Some older adults cited that having adequate knowledge and skills in using digital technology facilitated completion of PRMs electronically.  As cited by most older adults, administrative staff and healthcare professionals, older adults lacking adequate digital technology knowledge and skills made older adults feel uncomfortable and less confident when completing PRMs electronically. | [10, 36, 40, 45, 46, 48, 51, 52, 53] | 9 studies varied in methodological limitations  Strengths included clear aims (n = 9), appropriate methods (n = 9), justification of design (n = 9), recruitment strategy (n = 9), data collection (n = 9), ethics (n = 9) and clear statement of findings (n = 9)  Weaknesses related to discussing relationship between researcher and participant (no: n = 3) and data analysis (no: n = 1)    All studies have either minor or no methodological limitations  *Minor concerns* | Majority of studies conducted recently and contribute relevant data  Majority of studies conducted in hospital outpatient setting. Limited coverage of other healthcare settings and multi-disciplinary care.  Limited information provided on proportion of older adults above 65 years involved in study or being treated. 3 studies indicate mean or median age of patients close to or above 65 years  *Moderate concerns* | Good fit between data and finding  *No or very minor concerns* | Data from 9 of 22 studies contributed to this finding.  Studies cover views of older adults (n = 7), administrative staff (n = 1) and healthcare professionals (n = 2)  Minor concerns about richness of data in some studies (n = 5)  *Minor concerns* | **Moderate confidence**  Data was coherent. Minor concerns about methodological limitation and adequacy. Moderate concerns about relevance |
| *Health knowledge and literacy in general* | | | | | | | |
| 11 | Older adults and healthcare professionals cited that older adults lacking adequate health knowledge found it difficult to understand medical terminology and health information in questionnaires, that made it challenging to electronically complete PRMs. Healthcare professionals cited that some of these older adults found difficulty in discerning between different health conditions they experienced.  Caregivers experienced difficulty in understanding medical terminology, which acted as a barrier to electronic completion of PRMs on behalf of older adults.  Administrative staff cited that low literacy levels in older adults limited their reading, hindering electronic completion of PRMs. | [10, 36, 46, 48, 51, 53] | 6 studies varied in methodological limitations  Strengths included clear aims (n = 6), appropriate methods (n = 6), justification of design (n = 6), recruitment strategy (n = 6), data collection (n = 6), ethics (n = 6) and clear statement of findings (n = 6)  Weaknesses related to discussing relationship between researcher and participant (no: n = 1) and data analysis (no: n = 1)    All studies have either minor or no methodological limitations  *Minor concerns* | Majority of studies conducted recently and contribute relevant data  Majority of studies conducted in hospital outpatient setting. Limited coverage of other healthcare settings and multi-disciplinary care.  Limited information provided on proportion of older adults above 65 years involved in study or being treated. 2 studies indicate mean or median age of patients close to or above 65 years  *Moderate concerns* | Good fit between data and finding  *No or very minor concerns* | Data from 6 of 22 studies contributed to this finding.  Studies cover views of older adults (n = 2), caregivers (n = 1), administrative staff (n = 1) and healthcare professionals (n = 4)  Minor concerns about richness of data in some studies (n = 3)  *Moderate concerns* | **Moderate confidence**  Data was coherent. Minor concerns about methodological limitation. Moderate concerns about relevance and adequacy |
| *PRMs interpretation knowledge and skills* | | | | | | | |
| 12 | A few healthcare professionals reported that receiving a guideline for interpretation of PRMs responses facilitated electronic collection and use of PRMs. Most healthcare professionals reported the lack of knowledge and skills to interpret PRMs responses and address problem areas hindered electronic collection and use of PRMs. | [35, 38, 41] | 3 studies varied in methodological limitations  Strengths included clear aims (n = 3), appropriate methods (n = 3), justification of design (n = 3), ethics (n = 3) and data analysis (n=3)  Weaknesses related to recruitment strategy (no: n = 2), data collection (no: n = 1), discussing relationship between researcher and participant (no: n = 2) and clear statement of findings (no: n = 1)    2 of the 3 studies have minor or no methodological limitations  *Minor concerns* | 2 of the 3 studies conducted recently and contribute relevant data  All studies conducted in hospital outpatient setting. Limited coverage of other healthcare settings and multi-disciplinary care.  Limited information provided on proportion of older adults above 65 years being treated. 1 study indicated mean or median age of patients close to or above 65 years  *Moderate concerns* | Good fit between data and finding  *No or very minor concerns* | Data from 3 of 22 studies contributed to this finding.  Studies cover views of healthcare professionals (n = 3)  Minor concerns about richness of data in some studies (n = 2)  *Moderate concerns* | **Low confidence**  Data was coherent. Minor concerns about methodological limitation and relevance. Moderate concerns about adequacy |
| *Regular exposure enhancing health knowledge* | | | | | | | |
| 13 | Older adults and healthcare professionals perceived that completing PRMs electronically helped improve older adults’ health knowledge and awareness of their own health condition in the long term. | [41, 44, 46, 47, 48, 51] | 6 studies varied in methodological limitations  Strengths included clear aims (n = 6), appropriate methods (n = 6), justification of design (n = 6), data collection (n = 6), ethics (n = 6) and clear statement of findings (n = 6)  Weaknesses related to recruitment strategy (no: n = 1), discussing relationship between researcher and participant (no: n = 1) and data analysis (no: n = 1)    All studies have either minor or no methodological limitations  *Minor concerns* | Majority of studies conducted recently and contribute relevant data  Majority of studies conducted in hospital outpatient setting. Limited coverage of other healthcare settings and multi-disciplinary care.  Limited information provided on proportion of older adults above 65 years involved in study or being treated. 5 studies indicate mean or median age of patients close to or above 65 years  *Minor concerns* | Good fit between data and finding  *No or very minor concerns* | Data from 6 of 22 studies contributed to this finding.  Studies cover views of older adults (n = 5) and healthcare professionals (n = 3)  Very minor concerns about richness of data in some studies (n = 2)  *Minor concerns* | **High confidence**  Data was coherent. Minor concerns about methodological limitation, relevance and adequacy |
| *Rationale for PRMs collection and use* | | | | | | | |
| 14 | Older adults and healthcare professionals reported that older adults feeling obliged to complete PRMs for healthcare professionals without understanding the rationale for PRMs collection was a barrier to electronic completion of PRMs. | [35, 38, 45, 50] | 4 studies varied in methodological limitations  Strengths included clear aims (n = 4), appropriate methods (n = 4) and ethics (n = 4)  Weaknesses related to justification of design (no: n = 1), recruitment strategy (no: n = 3), data collection (no: n = 2), discussing relationship between researcher and participant (no: n = 3), data analysis (no: n = 1) and clear statement of findings (no: n = 1)    2 of the 4 studies have minor or no methodological limitations  *Moderate concerns* | Majority of studies conducted recently and contribute relevant data  All studies conducted in hospital outpatient setting. Limited coverage of other healthcare settings and multi-disciplinary care.  Limited information provided on proportion of older adults above 65 years involved in study or being treated. No studies indicate mean or median age of patients close to or above 65 years  *Moderate concerns* | Good fit between data and finding  *No or very minor concerns* | Data from 4 of 22 studies contributed to this finding.  Studies cover views of older adults (n = 1) and healthcare professionals (n = 3)  Very minor concerns about richness of data in some studies (n = 1)  *Moderate concerns* | **Low confidence**  Data was coherent. Moderate concerns about methodological limitation, relevance and adequacy |
| Thematic category: Motivation and incentives for capture and use of PRMs | | | | | | | |
| *Older adults’ motivation and incentives* | | | | | | | |
| 15 | Older adults reported the following factors as motivators for electronic completion of PRMs:   - owning responsibility to improve one’s own health and awareness of their health - providing adequate information about their health to healthcare professionals for care planning - helping advance health research, and help other patients improve health outcomes through research advancements - healthcare professionals reviewing, discussing and using PRMs responses during consultations to improve care - trust in healthcare provider (e.g., hospital asked to use digital PRM system) - self-monitoring of condition based on PRMs responses | [35, 40, 43, 44, 45, 46, 47, 48, 51] | 9 studies varied in methodological limitations  Strengths included clear aims (n = 9), appropriate methods (n = 9), justification of design (n = 9) and ethics (n = 9)  Weaknesses related to recruitment strategy (no: n = 2), data collection (no: n = 1), discussing relationship between researcher and participant (no: n = 4), data analysis (no: n = 2) and clear statement of findings (no: n = 2)    7 of the 9 studies have minor or no methodological limitations  *Minor concerns* | Majority of the studies conducted recently and contribute relevant data  Majority of studies conducted in hospital outpatient setting. Limited coverage of other healthcare settings and multi-disciplinary care.  Limited information provided on proportion of older adults above 65 years involved in study. 4 studies indicate mean or median age of patients close to or above 65 years  *Moderate concerns* | Good fit between data and finding  *No or very minor concerns* | Data from 9 of 22 studies contributed to this finding.  Studies cover views of older adults (n = 9)  Minor concerns about richness of data in some studies (n = 2)  *Minor concerns* | **Moderate confidence**  Data was coherent. Minor concerns about methodological limitation and adequacy. Moderate concerns about relevance |
| 16 | As cited by some older adults and healthcare professionals, older adults were demotivated to complete PRMs electronically if they believed it did not influence their health and the care they received, and when their healthcare professional did not review, discuss and use PRMs responses during their consultations.  Some older adults reported that a negative reaction to the PRMs responses by their healthcare professional discouraged them from completing PRMs electronically. | [35, 40, 43, 44, 45, 47, 48, 49, 50, 51] | 10 studies varied in methodological limitations  Strengths included clear aims (n = 10), appropriate methods (n = 10) and ethics (n = 10)  Weaknesses related to justification of design (no: n = 1) recruitment strategy (no: n = 3), data collection (no: n = 2), discussing relationship between researcher and participant (no: n = 5), data analysis (no: n = 3) and clear statement of findings (no: n = 3)    7 of the 10 studies have minor or no methodological limitations  *Minor concerns* | Majority of the studies conducted recently and contribute relevant data  Majority of studies conducted in hospital outpatient setting. Limited coverage of other healthcare settings and multi-disciplinary care.  Limited information provided on proportion of older adults above 65 years involved in studies or being treated. 3 studies indicate mean or median age of patients close to or above 65 years  *Moderate concerns* | Good fit between data and finding  *No or very minor concerns* | Data from 10 of 22 studies contributed to this finding.  Studies cover views of older adults (n = 8) and healthcare professionals (n = 2)  Minor concerns about richness of data in some studies (n = 3)  *Minor concerns* | **Moderate confidence**  Data was coherent. Minor concerns about methodological limitation and adequacy. Moderate concerns about relevance |
| *Healthcare professionals’ motivation and incentives* | | | | | | | |
| 17 | Healthcare professionals reported the following factors as motivators to electronic collection and use of PRMs:   - involvement of patients to voice their perception of health through PRMs completion - PRMs response data completeness - PRMs responses informing quality improvements and research advancements - enhanced documentation based on PRMs responses - collaboration with multi-disciplinary care teams to improve care based on PRMs responses - regulatory directives (e.g., PRMs data as part of data registries) mandating PRMs data collection - shared decision making and tailored care as a result of PRMs use - immediate access to PRMs responses and meaningful presentation of PRMs responses for clinical decision-making | [34, 35, 36, 38, 39, 41, 43, 46, 50, 53] | 10 studies varied in methodological limitations  Strengths included clear aims (n = 10), appropriate methods (n = 10) and ethics (n = 10)  Weaknesses related to justification of design (no: n = 1), recruitment strategy (no: n = 5), data collection (no: n = 2), discussing relationship between researcher and participant (no: n = 7), data analysis (no: n = 4) and clear statement of findings (no: n = 2)    6 of the 10 studies have minor or no methodological limitations  *Minor concerns* | Majority of the studies conducted recently and contribute relevant data  Majority of studies conducted in hospital outpatient setting. Limited coverage of other healthcare settings and multi-disciplinary care.  Limited information provided on proportion of older adults above 65 years being treated. 2 studies indicate mean or median age of patients close to or above 65 years  *Moderate concerns* | Good fit between data and finding  *No or very minor concerns* | Data from 10 of 22 studies contributed to this finding.  Studies cover views of healthcare professionals (n = 10)  Minor concerns about richness of data in some studies (n = 3)  *Minor concerns* | **Moderate confidence**  Data was coherent. Minor concerns about methodological limitation and adequacy. Moderate concerns about relevance |
| 18 | Healthcare professionals reported that the following factors demotivated them to electronically collect and use PRMs:   - believing that PRMs did not influence the care they provided - discrepancies in health assessment between the patient and themselves - PRMs responses providing insufficient information - organisational or regulatory directive to collect and use PRMs, in absence of clear messaging of rationale and benefits for collection and use of PRMs - believing PRMs collection and use disrupts patient care duties - non-completion of PRMs questions and low response rates - non-discipline specific PRMs collection and use - healthcare professional characteristics associated with older age, higher length of service and not reviewing PRMs regularly - lack of immediate access to and meaningful presentation of PRMs responses for clinical decision-making | [34, 35, 36, 38, 43, 44, 46, 50, 53] | 9 studies varied in methodological limitations  Strengths included clear aims (n = 9), appropriate methods (n = 9) and ethics (n = 9)  Weaknesses related to justification of design (no: n = 1), recruitment strategy (no: n = 4), data collection (no: n = 2), discussing relationship between researcher and participant (no: n = 6), data analysis (no: n = 4) and clear statement of findings (no: n = 2)    5 of the 9 studies have minor or no methodological limitations  *Minor concerns* | Majority of studies conducted recently and contribute relevant data  Majority of studies conducted in hospital outpatient setting. Limited coverage of other healthcare settings and multi-disciplinary care.  Limited information provided on proportion of older adults above 65 years being treated. 2 studies indicate mean or median age of patients close to or above 65 years  *Moderate concerns* | Good fit between data and finding  *No or very minor concerns* | Data from 9 of 22 studies contributed to this finding.  Studies cover views of healthcare professionals (n = 8)  Very minor concerns about richness of data in some studies (n = 3)  *Minor concerns* | **Moderate confidence**  Data was coherent. Minor concerns about methodological limitation and adequacy. Moderate concerns about relevance |
| *Acceptance of intervention by older adults* | | | | | | | |
| 19 | Administrative staff cited that acceptance of electronic completion of PRMs by older adults motivated them to support the process of collecting PRMs electronically | [36] | Strengths included clear aims, appropriate methods, justification of design, recruitment strategy, data collection, ethics, data analysis and clear statement of findings  Weaknesses related to discussing relationship between researcher and participant    The study had minor methodological limitations  *Minor concerns* | Study conducted recently and contribute relevant data  Study conducted in hospital outpatient setting. Limited coverage of other healthcare settings and multi-disciplinary care.  Limited information provided on proportion of older adults above 65 years being treated. Study does not indicate mean or median age of patients close to or above 65 years  *Moderate concerns* | Good fit between data and finding  *No or very minor concerns* | Data from 1 of 22 studies contributed to this finding.  Studies cover views of administrative staff (n = 1)  Minor concerns about richness of data in the study  *Moderate concerns* | **Low confidence**  Data was coherent. Minor concerns about methodological limitation. Moderate concerns about relevance and adequacy  *(This finding has been downgraded twice as only one study supports this finding)* |
| *Location of electronic PRMs administration* | | | | | | | |
| 20 | Older adults found completing PRMs questions and reviewing responses from home or remote location (outside the clinic) was convenient and facilitated electronic completion of PRMs.  The need to spend extra time in the clinic to complete PRMs, incurring transport and parking costs to be physically present in the clinic, hygiene and data privacy risks associated with using shared devices and feeling rushed were barriers to completing PRMs electronically in the clinic, as reported by most older adults.  Incomplete questionnaires due to limited time for older adults to complete PRMs in the clinic was reported as a barrier to electronic collection and use of PRMs by some healthcare professionals. Some healthcare professionals perceived having an in-clinic option to complete PRMs may facilitate older adults’ PRMs completion rates | [10, 33, 35, 38, 42, 43, 45, 47, 48, 51, 53] | 11 studies varied in methodological limitations  Strengths included clear aims (n = 11), appropriate methods (n = 11) and ethics (n = 11)  Weaknesses related to justification of design (no: n = 2), recruitment strategy (no: n = 3), data collection (no: n = 2), discussing relationship between researcher and participant (no: n = 5), data analysis (no: n = 3) and clear statement of findings (no: n = 2)    7 of the 11 studies have minor or no methodological limitations  *Minor concerns* | Majority of studies conducted recently and contribute relevant data  Majority of studies conducted in hospital outpatient setting. Limited coverage of other healthcare settings and multi-disciplinary care.  Limited information provided on proportion of older adults above 65 years involved in studies or being treated. 4 studies indicate mean or median age of patients close to or above 65 years  *Moderate concerns* | Good fit between data and finding  *No or very minor concerns* | Data from 11 of 22 studies contributed to this finding.  Studies cover views of older adults (n = 9) and healthcare professionals (n = 2)  Minor concerns about richness of data in some studies (n = 3)  *Minor concerns* | **Moderate confidence**  Data was coherent. Minor concerns about methodological limitation and adequacy. Moderate concerns about relevance |
| *Timing and frequency of electronic PRMs collection* | | | | | | | |
| 21 | Some older adults reported that completing PRMs at a regular frequency (e.g., once a week or once a month) and close to the day of consultation facilitated electronic completion of PRMs. Infrequent completion of PRMs that led to no changes in health, and that reduced familiarity with the electronic system for PRMs completion were cited as barriers to electronic completion of PRMs by some older adults.  Healthcare professionals cited that the lack of PRMs completion closer to the day of consultation was a barrier to electronic collection and use of PRMs. | [42, 44, 45, 49, 50, 53] | 6 studies varied in methodological limitations  Strengths included clear aims (n = 6), appropriate methods (n = 6) and ethics (n = 6)  Weaknesses related to justification of design (no: n = 2), recruitment strategy (no: n = 1), data collection (no: n = 1), discussing relationship between researcher and participant (no: n = 2), data analysis (no: n = 3) and clear statement of findings (no: n = 1)    4 of the 6 studies have minor or no methodological limitations  *Minor concerns* | Majority of studies conducted recently and contribute relevant data  All studies conducted in hospital outpatient setting. Limited coverage of other healthcare settings and multi-disciplinary care.  Limited information provided on proportion of older adults above 65 years involved in studies or being treated. 2 studies indicate mean or median age of patients close to or above 65 years  *Moderate concerns* | Good fit between data and finding  *No or very minor concerns* | Data from 6 of 22 studies contributed to this finding.  Studies cover views of older adults (n = 4) and healthcare professionals (n = 2)  Minor concerns about richness of data in some studies (n = 3)  *Minor concerns* | **Moderate confidence**  Data was coherent. Minor concerns about methodological limitation and adequacy. Moderate concerns about relevance |
| *Access to additional resources based on PRMs responses* | | | | | | | |
| 22 | Most older adults and healthcare professionals reported that access to additional medical or non-medical resources (e.g., self-management guidance or social support) based on PRMs responses encouraged older adults to electronically complete PRMs.  Some healthcare professionals reporting lack of resources to offer (e.g., education, social services), and some older adults reporting inability to access resources (e.g., getting questions answered from healthcare professional or personalised information) based on PRMs responses were barriers to electronic collection and use of PRMs | [35, 38, 40, 44, 47, 48, 49, 50, 51, 52] | 10 studies varied in methodological limitations  Strengths included clear aims (n = 10), appropriate methods (n = 10) and ethics (n = 10)  Weaknesses related to justification of design (no: n = 1), recruitment strategy (no: n = 4), data collection (no: n = 2), discussing relationship between researcher and participant (no: n = 6), data analysis (no: n = 2) and clear statement of findings (no: n = 2)    8 of the 10 studies have minor or no methodological limitations  *Minor concerns* | Majority of studies conducted recently and contribute relevant data  Majority of studies conducted in hospital outpatient setting. Limited coverage of other healthcare settings and multi-disciplinary care.  Limited information provided on proportion of older adults above 65 years involved in studies or being treated. 4 studies indicate mean or median age of patients close to or above 65 years  *Moderate concerns* | Good fit between data and finding  *No or very minor concerns* | Data from 10 of 22 studies contributed to this finding.  Studies cover views of older adults (n = 8) and healthcare professionals (n = 3)  Minor concerns about richness of data in some studies (n = 4)  *Minor concerns* | **Moderate confidence**  Data was coherent. Minor concerns about methodological limitation and adequacy. Moderate concerns about relevance |
| Thematic category: Emotional experience | | | | | | | |
| 23 | Some older adults reported that answering questions about their health conditions made them emotionally elevated, encouraging them to complete PRMs electronically. Some healthcare professionals believed that older adults may feel less embarrassed to electronically complete PRMs especially related to personal and sensitive topics (e.g., related to mental health, sex life and social determinants) rather than talk about them initially, which may facilitate older adults’ completion of PRMs electronically.  Some older adults and healthcare professionals cited that emotional distress caused by having to respond to questions on health conditions, and personal and sensitive topics were barriers to older adults’ completion of PRMs electronically. Anxiety caused by the limited time available to complete PRMs in the clinic was reported as a barrier to electronically completing PRMs by some older adults. | [10, 33, 38, 41, 43, 44, 48, 50, 51] | 9 studies varied in methodological limitations  Strengths included clear aims (n = 9), appropriate methods (n = 9) and ethics (n = 9)  Weaknesses related to justification of design (no: n = 2), recruitment strategy (no: n = 2), data collection (no: n = 2), discussing relationship between researcher and participant (no: n = 3), data analysis (no: n = 3) and clear statement of findings (no: n = 1)    6 of the 9 studies have minor or no methodological limitations  *Minor concerns* | Majority of studies conducted recently and contribute relevant data  Majority of studies conducted in hospital outpatient setting. Limited coverage of other healthcare settings and multi-disciplinary care.  Limited information provided on proportion of older adults above 65 years involved in studies or being treated. 4 studies indicate mean or median age of patients close to or above 65 years  *Moderate concerns* | Good fit between data and finding  *No or very minor concerns* | Data from 9 of 22 studies contributed to this finding.  Studies cover views of older adults (n = 6) and healthcare professionals (n = 3)  Minor concerns about richness of data in some studies (n = 3)  *Minor concerns* | **Moderate confidence**  Data was coherent. Minor concerns about methodological limitation and adequacy. Moderate concerns about relevance |
| Thematic category: Older adults’ autonomy | | | | | | | |
| 24 | Some older adults reported that they knew best about their health and being guided by own choice to complete PRMs facilitated electronic completion of PRMs.  As cited by some older adults and healthcare professionals, the influence from family, caregivers, administrative staff or healthcare professionals sometimes led to the suppression of the older adults’ voice, discouraging older adults to complete PRMs electronically. | [33, 36, 39, 427, 45] | 5 studies varied in methodological limitations  Strengths included clear aims (n = 4), appropriate methods (n = 4), ethics (n = 4) and clear statement of findings (n = 4)  Weaknesses related to justification of design (no: n = 2), recruitment strategy (no: n = 1), data collection (no: n = 1), discussing relationship between researcher and participant (no: n = 3) and data analysis (no: n = 2)    3 of the 5 studies have minor or no methodological limitations  *Minor concerns* | Majority of studies conducted recently and contribute relevant data  Majority of studies conducted in hospital outpatient setting. Limited coverage of other healthcare settings and multi-disciplinary care.  Limited information provided on proportion of older adults above 65 years involved in studies or being treated. 2 studies indicate mean or median age of patients close to or above 65 years  *Moderate concerns* | Good fit between data and finding  *No or very minor concerns* | Data from 5 of 22 studies contributed to this finding.  Studies cover views of older adults (n = 3) and healthcare professionals (n = 2)  Very minor concerns about richness of data in some studies (n = 2)  *Minor concerns* | **Moderate confidence**  Data was coherent. Minor concerns about methodological limitation and adequacy. Moderate concerns about relevance |
| Thematic category: Patient – healthcare professional communication | | | | | | | |
| 25 | Structured communication during consultations (e.g., discussion of results focusing on problem areas and changes over time), enhanced patient – healthcare professional interactions and patient involvement in voicing their perception of health were reported as facilitators to electronic collection and use of PRMs by some older adults and healthcare professionals.  Some older adults and healthcare professionals reported that PRMs completion made the patient – healthcare professional interactions impersonal, discouraging the electronic completion of PRMs. Some older adults feared that relying on electronic systems to communicate about health may lead to the replacement of face-to-face consultations, and lack of support from healthcare professionals and the healthcare service. | [34, 35, 36, 38, 39, 40, 41, 43, 44, 45, 46, 47, 48, 50, 51] | 15 studies varied in methodological limitations  Strengths included clear aims (n = 15), appropriate methods (n = 15) and ethics (n = 15)  Weaknesses related to justification of design (no: n = 1), recruitment strategy (no: n = 6), data collection (no: n = 2), discussing relationship between researcher and participant (no: n = 9), data analysis (no: n = 4) and clear statement of findings (no: n = 2)    11 of the 15 studies have minor or no methodological limitations  *Minor concerns* | Majority of studies conducted recently and contribute relevant data  Majority of studies conducted in hospital outpatient setting. Limited coverage of other healthcare settings and multi-disciplinary care.  Limited information provided on proportion of older adults above 65 years involved in studies or being treated. 5 studies indicate mean or median age of patients close to or above 65 years  *Moderate concerns* | Good fit between data and finding  *No or very minor concerns* | Data from 15 of 22 studies contributed to this finding.  Studies cover views of older adults (n = 9) and healthcare professionals (n = 10)  Very minor concerns about richness of data in some studies (n = 3)  *Minor concerns* | **Moderate confidence**  Data was coherent. Minor concerns about methodological limitation and adequacy. Moderate concerns about relevance |
| Thematic category: Workflow | | | | | | | |
| *Efficiencies, time constraints and changes to work routines* | | | | | | | |
| 26 | Facilitators to electronic collection and use of PRMs reported by some healthcare professionals were improved clinic efficiency through the use of electronic systems to screen PRMs responses and determine patient needs prior to the consultation, reduced clerical time due to patients independently completing PRMs electronically, enhanced EHR documentation based on PRMs responses and automatic distribution of questionnaire, and time saved through focussed patient assessments based on PRMs responses.  Some older adults and administrative staff reported that knowing healthcare professionals save time using the electronic system to access and use PRMs encouraged engagement with electronic completion of PRMs.  Barriers to electronic collection and use of PRMs cited by many healthcare professionals were time constraints and new routines associated with incorporation of PRMs in care and use of electronic systems to access PRMs responses, inundation of PRMs response data resulting in having too many things to discuss with the patient, delays to clinic workflows associated with challenges in PRMs administration (e.g., questionnaires not working or patients not arriving early to complete PRMs in time before the appointment), challenges with seamless data access and additional work created by having to cooperate with other multi-disciplinary healthcare professionals. | [34, 35, 36, 38, 39, 41, 43, 44, 45, 46, 51, 53] | 12 studies varied in methodological limitations  Strengths included clear aims (n = 12), appropriate methods (n = 12), justification of design (n = 12) and ethics (n = 12)  Weaknesses related to recruitment strategy (no: n = 4), data collection (no: n = 1), discussing relationship between researcher and participant (no: n = 6), data analysis (no: n = 3) and clear statement of findings (no: n = 2)    10 of the 12 studies have minor or no methodological limitations  *Minor concerns* | Majority of studies conducted recently and contribute relevant data  Majority of studies conducted in hospital outpatient setting. Limited coverage of other healthcare settings and multi-disciplinary care.  Limited information provided on proportion of older adults above 65 years involved in studies or being treated. 3 studies indicate mean or median age of patients close to or above 65 years  *Moderate concerns* | Good fit between data and finding  *No or very minor concerns* | Data from 12 of 22 studies contributed to this finding.  Studies cover views of older adults (n = 1), administrative staff (n = 1) healthcare professionals (n = 11)  Minor concerns about richness of data in some studies (n = 5)  *Minor concerns* | **Moderate confidence**  Data was coherent. Minor concerns about methodological limitation and adequacy. Moderate concerns about relevance |
| *Additional work with PRMs completion* | | | | | | | |
| 27 | Some older adults and healthcare professionals cited that older adults perceived PRMs completion was additional work and use of electronic systems to complete PRMs was time consuming, hindering older adults from electronically completing PRMs | [43, 45, 47, 50] | 4 studies varied in methodological limitations  Strengths included clear aims (n = 4), appropriate methods (n = 4) and ethics (n = 4)  Weaknesses related to justification of design (no: n = 1), recruitment strategy (no: n = 2), data collection (no: n = 1), discussing relationship between researcher and participant (no: n = 3), data analysis (no: n = 2) and clear statement of findings (no: n = 1)    2 of the 4 studies have minor or no methodological limitations  *Moderate concerns* | Majority of studies conducted recently and contribute relevant data  All studies conducted in hospital outpatient setting. Limited coverage of other healthcare settings and multi-disciplinary care.  Limited information provided on proportion of older adults above 65 years involved in studies or being treated. 1 study indicates mean or median age of patients close to or above 65 years  *Moderate concerns* | Good fit between data and finding  *No or very minor concerns* | Data from 4 of 22 studies contributed to this finding.  Studies cover views of older adults (n = 3) and healthcare professionals (n = 1)  Minor concerns about richness of data in some studies (n = 2)  *Moderate concerns* | **Low confidence**  Data was coherent. Moderate concerns about methodological limitation, adequacy and relevance  *(Downgraded twice as there are 3 components with moderate concerns)* |
| Thematic category: Organisational factors | | | | | | | |
| *Team culture and collaboration* | | | | | | | |
| 28 | Multi-disciplinary team communication, positive team culture and peer to peer support were cited as facilitators to electronic collection and use of PRMs by some healthcare professionals, while some reported that challenges in multi-disciplinary collaboration and different levels of motivation within the team to collect and use PRMs were barriers electronic collection and use of PRMs. | [34, 35, 36, 39, 41] | 5 studies varied in methodological limitations  Strengths included clear aims (n = 5), appropriate methods (n = 5), justification of design (n = 5) and ethics (n = 5)  Weaknesses related to recruitment strategy (no: n = 3), data collection (no: n = 1), discussing relationship between researcher and participant (no: n = 4), data analysis (no: n = 1) and clear statement of findings (no: n = 1)    3 of the 5 studies have minor or no methodological limitations  *Minor concerns* | Majority of studies conducted recently and contribute relevant data  Majority of studies conducted in hospital outpatient setting. Limited coverage of other healthcare settings and multi-disciplinary care.  Limited information provided on proportion of older adults above 65 years involved in studies or being treated. 1 study indicates mean or median age of patients close to or above 65 years  *Moderate concerns* | Good fit between data and finding  *No or very minor concerns* | Data from 5 of 22 studies contributed to this finding.  Studies cover views of healthcare professionals (n = 5)  Very minor concerns about richness of data in all studies  *Moderate concerns* | **Moderate confidence**  Data was coherent. Minor concerns about methodological limitation. Moderate concerns about adequacy and relevance |
| *Leadership and champions* | | | | | | | |
| 29 | Healthcare professionals reported that supportive leadership and having champions facilitated electronic collection and use of PRMs. They valued the mentorship they received and buy-in from their leadership | [34, 36, 39] | 3 studies varied in methodological limitations  Strengths included clear aims (n = 3), appropriate methods (n = 3), justification of design (n = 3), data collection (n = 3), ethics (n = 3) and clear statement of findings (n = 3)  Weaknesses related to recruitment strategy (no: n = 2), discussing relationship between researcher and participant (no: n = 3) and data analysis (no: n = 1)    2 of the 3 studies have minor or no methodological limitations  *Minor concerns* | All studies conducted recently and contribute relevant data  Majority of studies conducted in hospital outpatient setting. Limited coverage of other healthcare settings and multi-disciplinary care.  Limited information provided on proportion of older adults above 65 being treated.  *Moderate concerns* | Good fit between data and finding  *No or very minor concerns* | Data from 3 of 22 studies contributed to this finding.  Studies cover views of healthcare professionals (n = 3)  Minor concerns about richness of data in all studies (n = 1)  *Moderate concerns* | **Moderate confidence**  Data was coherent. Minor concerns about methodological limitation. Moderate concerns about adequacy and relevance |
| *Change management* | | | | | | | |
| 30 | Some healthcare professionals cited that the flexibility provided to rapidly iterate the electronic system for better acceptance and having a clearly communicated operational process facilitate the electronic collection and use of PRMs. Lack of uniformity in the implementation process and lack of established operational workflows (e.g., training and accessing PRMs electronically) were reported as barriers to electronic collection and use of PRMs by some healthcare professionals.  Older adults and administrative staff believed training and support workflows (e.g., trouble shoot technical problems) facilitated the electronic collection of PRMs. | [34, 36, 38, 43, 44, 46, 47, 53] | 8 studies varied in methodological limitations  Strengths included clear aims (n = 8), appropriate methods (n = 8), justification of design (n = 8), data collection (n = 8) and ethics (n = 8)  Weaknesses related to recruitment strategy (no: n = 3), discussing relationship between researcher and participant (no: n = 5), data analysis (no: n = 3) and clear statement of findings (no: n = 1)    6 of the 8 studies have minor or no methodological limitations  *Minor concerns* | Majority of studies conducted recently and contribute relevant data  All studies conducted in hospital outpatient setting. Limited coverage of other healthcare settings and multi-disciplinary care.  Limited information provided on proportion of older adults above 65 being treated. 3 studies indicate mean or median age of patients close to or above 65 years  *Moderate concerns* | Good fit between data and finding  *No or very minor concerns* | Data from 8 of 22 studies contributed to this finding.  Studies cover views of older adults (n = 2), administrative staff (n = 1) and healthcare professionals (n = 6)  Minor concerns about richness of data in all studies (n = 3)  *Moderate concerns* | **Moderate confidence**  Data was coherent. Minor concerns about methodological limitation. Moderate concerns about adequacy and relevance |
| *Resources required to manage collection and use of PRMs* | | | | | | | |
| 31 | Some older adults reported that receiving adequate technical support and education would facilitate completion of PRMs electronically. The lack of technical support and necessary education to help with completing PRMs electronically were cited as barriers by older adults. The technical support available to resolve any technical problems was cited as a facilitator to electronic completion of PRMs by some older adults.  Healthcare professionals reported that adequate education, support staff, support in the form of reminders during consultation hours prompting them to review PRMs responses, digital technology infrastructure that support seamless data integration, and government funding to cover digital technology infrastructure costs were facilitators to the electronic collection and use of PRMs. Barriers to electronic collection and use of PRMs cited by healthcare professionals were lack of digital technology infrastructure to support integration of PRMs in EHRs or other platforms clinicians use, education (e.g., to use electronic systems and interpret PRMs responses), high costs and lack of support staff. Healthcare professionals believed adequate education, digital technology infrastructure that allowed for integration of PRMs with EHRs, funding and support staff facilitated electronic collection and use of PRMs.  Administrative staff particularly felt that access to interpreter services, technical support staff and adequate education facilitated electronic collection of PRMs. | [34, 35, 36, 38, 39, 40, 43, 44, 45, 46, 50, 52] | 12 studies varied in methodological limitations  Strengths included clear aims (n = 12), appropriate methods (n = 12) and ethics (n = 12)  Weaknesses related to justification of design (no: n = 1), recruitment strategy (no: n = 5), data collection (no: n = 2), discussing relationship between researcher and participant (no: n = 9), data analysis (no: n = 4) and clear statement of findings (no: n = 2)    8 of the 12 studies have minor or no methodological limitations  *Minor concerns* | Majority of studies conducted recently and contribute relevant data  Majority of studies conducted in hospital outpatient setting. Limited coverage of other healthcare settings and multi-disciplinary care.  Limited information provided on proportion of older adults above 65 years involved in studies or being treated. 3 studies indicate mean or median age of patients close to or above 65 years  *Moderate concerns* | Good fit between data and finding  *No or very minor concerns* | Data from 12 of 22 studies contributed to this finding.  Studies cover views of older adults (n = 4), administrative staff (n = 1) and healthcare professionals (n = 8)  Minor concerns about richness of data in all studies (n = 3)  *Moderate concerns* | **Moderate confidence**  Data was coherent. Minor concerns about methodological limitation. Moderate concerns about adequacy and relevance |
| Thematic category: PRMs Questionnaire selection and design | | | | | | | |
| *Questionnaire length and complexity of questions* | | | | | | | |
| 32 | Some older adults cited that having simple, easy-to-understand questions facilitated electronic completion of PRMs. Some older adults and healthcare professionals cited that lengthy questionnaires containing hard to comprehend questions and those that took a long period of time to complete were barriers to electronic completion of PRMs.  Some healthcare professionals believed that questions should be asked in a way that would highlight the patient’s problems and that the overlapping number of questions asked repeatedly during each clinic visit may be reduced by sharing PRMs responses between clinicians. | [33, 36, 38, 41, 42, 44, 45, 46, 47, 48, 51, 53] | 12 studies varied in methodological limitations  Strengths included clear aims (n = 12), appropriate methods (n = 12), ethics (n = 12) and clear statement of findings (n = 12)  Weaknesses related to justification of design (no: n = 2), recruitment strategy (no: n = 2), data collection (no: n = 1), discussing relationship between researcher and participant (no: n = 4) and data analysis (no: n = 3)  10 of the 12 studies have minor or no methodological limitations  *Minor concerns* | Majority of studies conducted recently and contribute relevant data  Majority of studies conducted in hospital outpatient setting. Limited coverage of other healthcare settings and multi-disciplinary care.  Limited information provided on proportion of older adults above 65 years involved in study or being treated. 7 studies indicate mean or median age of patients close to or above 65 years  *Minor concerns* | Good fit between data and finding  *No or very minor concerns* | Data from 12 of 22 studies contributed to this finding.  Studies cover views of older adults (n = 7) and healthcare professionals (n = 5)  Minor concerns about richness of data in some studies (n = 4)  *Minor concerns* | **High confidence**  Data was coherent. Minor concerns about methodological limitation, relevance and adequacy |
| *Questions relevant to patient’s health* | | | | | | | |
| 33 | Some older adults and healthcare professionals reported that having questions specific to the patient’s health (e.g., behavioural activation, mental health and sexual function) facilitated electronic collection and use of PRMs. Some older adults and healthcare professionals cited that the lack of specific questions addressing patient’s health (e.g., co-morbid conditions and specific disease) hindered electronic collection and use of PRMs. | [36, 38, 43, 47, 48, 51, 53] | 7 studies varied in methodological limitations  Strengths included clear aims (n = 7), appropriate methods (n = 7), justification of design (n = 7), data collection (n = 7) and ethics (n = 7)  Weaknesses related to recruitment strategy (no: n = 2), discussing relationship between researcher and participant (no: n = 4), data analysis (no: n = 1) and clear statement of findings (no: n = 1)    6 of the 7 studies have minor or no methodological limitations  *Minor concerns* | Majority of studies conducted recently and contribute relevant data  Majority of studies conducted in hospital outpatient setting. Limited coverage of other healthcare settings and multi-disciplinary care.  Limited information provided on proportion of older adults above 65 years involved in study or being treated. 2 studies indicate mean or median age of patients close to or above 65 years  *Moderate concerns* | Good fit between data and finding  *No or very minor concerns* | Data from 7 of 22 studies contributed to this finding.  Studies cover views of older adults (n = 4) and healthcare professionals (n = 5)  Minor concerns about richness of data in some studies (n = 1)  *Minor concerns* | **Moderate confidence**  Data was coherent. Minor concerns about methodological limitation and adequacy. Moderate concerns about relevance |
| *PRMs response capture option* | | | | | | | |
| 34 | Some older adults found a free-text field to add comments to qualify the numerical rating of their health facilitated electronic completion of PRMs. The lack of a free text field to capture patient’s health was cited as a barrier by some older adults and healthcare professionals. Some older adults reported they could not use the free text field due to difficulties in information articulation or if they experienced dyslexia, hindering electronic completion of PRMs.  Most healthcare professionals found the numerical rating of patient health as more useful, while some healthcare professionals found free text description of patient health as more useful. | [38, 39, 41, 42, 43, 44, 48, 51] | 8 studies varied in methodological limitations  Strengths included clear aims (n = 8), appropriate methods (n = 8), data collection (n = 8) and ethics (n = 8)  Weaknesses related to justification of design (n = 1), recruitment strategy (no: n = 2), discussing relationship between researcher and participant (no: n = 4), data analysis (no: n = 2) and clear statement of findings (no: n = 1)    6 of the 8 studies have minor or no methodological limitations  *Minor concerns* | Majority of studies conducted recently and contribute relevant data  Majority of studies conducted in hospital outpatient setting. Limited coverage of other healthcare settings and multi-disciplinary care.  Limited information provided on proportion of older adults above 65 years involved in study or being treated. 4 studies indicate mean or median age of patients close to or above 65 years  *Moderate concerns* | Good fit between data and finding  *No or very minor concerns* | Data from 8 of 22 studies contributed to this finding.  Studies cover views of older adults (n = 4) and healthcare professionals (n = 4)  Minor concerns about richness of data in some studies (n = 2)  *Minor concerns* | **Moderate confidence**  Data was coherent. Minor concerns about methodological limitation and adequacy. Moderate concerns about relevance |
